# Functional Characterization of Novel Sesquiterpene Synthases from Indian Sandalwood, *Santalum album*

**DOI:** 10.1038/srep10095

**Published:** 2015-05-15

**Authors:** Prabhakar Lal Srivastava, Pankaj P. Daramwar, Ramakrishnan Krithika, Avinash Pandreka, S. Shiva Shankar, Hirekodathakallu V. Thulasiram

**Affiliations:** 1 Chemical Biology Unit, Division of Organic Chemistry, CSIR- National Chemical Laboratory, Dr. Homi Bhabha Road, Pune. 411008; 2 CSIR-Institute of Genomics and Integrative Biology, Mall Road, New Delhi. 110007

## Abstract

Indian Sandalwood, *Santalum album* L. is highly valued for its fragrant heartwood oil and is dominated by a blend of sesquiterpenes. Sesquiterpenes are formed through cyclization of farnesyl diphosphate (FPP), catalyzed by metal dependent terpene cyclases. This report describes the cloning and functional characterization of five genes, which encode two sesquisabinene synthases (*SaSQS1*, *SaSQS2*), bisabolene synthase (*SaBS*), santalene synthase (*SaSS*) and farnesyl diphosphate synthase (*SaFDS*) using the transcriptome sequencing of *S. album*. Using Illumina next generation sequencing, 33.32 million high quality raw reads were generated, which were assembled into 84,094 unigenes with an average length of 494.17 bp. Based on the transcriptome sequencing, five sesquiterpene synthases *SaFDS*, *SaSQS1*, *SaSQS2*, *SaBS* and *SaSS* involved in the biosynthesis of FPP, sesquisabinene, β-bisabolene and santalenes, respectively, were cloned and functionally characterized. Novel sesquiterpene synthases (*SaSQS1* and *SaSQS2*) were characterized as isoforms of sesquisabinene synthase with varying kinetic parameters and expression levels. Furthermore, the feasibility of microbial production of sesquisabinene from both the unigenes, *SaSQS1* and *SaSQS2* in non-optimized bacterial cell for the preparative scale production of sesquisabinene has been demonstrated. These results may pave the way for *in vivo* production of sandalwood sesquiterpenes in genetically tractable heterologous systems.

Terpenoid or isoprenoid compounds are the most ancient and diverse collection of natural products and are found in all forms of life. Over 70,000 individual structures, containing a truly incredible array of carbon skeletons and functional groups have been reported^1^. These structurally and stereochemically distinct molecules play crucial roles in plants, including hormones^2^, photosynthetic pigments^3^, electron carriers^4^, structural components of membrane^5^,^6^, as well as in communication and defense^7^. Indeed, the structurally diverse collection of isoprenoid compounds is constructed from two simple five-carbon building blocks, isopentenyl diphosphate (IPP) and dimethylallyl diphosphate (DMAPP), which in-turn are synthesized through mevalonate (MVA) or methylerythritol phosphate (MEP) pathway. The allylic isoprenoid diphosphate (DMAPP) undergoes coupling with one or more IPP molecules to form 1′-4 linkage characteristic of head-to-tail condensation to form prenyl diphosphates, geranyl diphosphate (GPP), farnesyl diphosphate (FPP) and geranylgeranyl diphosphate (GGPP). These short chain prenyl diphosphates subsequently undergo cyclization reaction catalyzed by terpene cyclases and ultimately, the functional group modifications catalyzed by CYP450 mono-oxygenase systems to generate functionally active terpenoids^8^. Farnesyl diphosphate synthase (FDS), a key chain elongation enzyme in isoprenoid biosynthesis, catalyzes the electrophilic condensation of one or two molecules of IPP (C_5_) with the allylic carbocations generated from allylic diphosphates, GPP (C_10_) or DMAPP (C_5_), respectively, to produce FPP (C_15_) that lies at the juncture of various isoprenoid biosynthetic branches, including sesquiterpene biosynthesis^9^. Highly evolvable sesquiterpene synthases catalyze the multistep conversions of (*E*,*E*)-FPP into numerous acyclic and cyclic structures through a series of carbocationic rearrangements, alkylations, hydride shifts and cyclizations which are initiated by ionization of the diphosphate anion resulting in the generation of reactive farnesyl carbocation^10^,^11^. Till date, over 7000 sesquiterpene molecules with more than 300 stereo-chemically distinct hydrocarbon skeletons are reported^12^. Although, sesquiterpenes are traditionally used as flavors and fragrances, they are also known to possess various biological properties including anticancer^13^ and antimalarial^14^ activities. In recent years, sesquiterpenes of farnesene, bisabolene and sabinene skeletons have been recognized as replacements for petroleum-derived jet-engine fuels^15^,^16^.

The endangered Sandalwood or Chandan (*Santalum album* L.) is known worldwide for its fragrant oil content that has a pleasant and woody odour. The essential oil obtained from the well matured *S. album* tree is represented by a mixture of sesquiterpenes such as: (*Z*)-α-santalol, (*Z*)-β-santalol, (*Z*)-*epi*-β-santalol, (*Z*)-α-*trans*-bergamotol, α-bisabolol, (*Z*)-lanceol, sesquisabinene hydrate and (*E,E*)-farnesol, along with small amounts (up to 2%) of corresponding precursor sesquiterpene hydrocarbons^17^,^18^,^19^, where (*Z*)-α-santalol and (*Z*)-β-santalol constitute the major active components^20^,^21^. The essential oil of sandalwood, usually obtained by steam distillation of chips and billets cut from the heartwood, is widely used for several purposes such as in perfumery, cosmetics, aromatherapy, as an antidepressant, anti-inflammatory, antifungal, astringent, sedative, insecticide, antiseptic and in sacred unguents^22^,^23^,^24^. The first committed step in sandalwood sesquiterpenoid biosynthesis is the cyclization of farnesyl diphosphate (FPP) by sesquiterpene synthases to yield sesquiterpene hydrocarbons (Fig. 1), which are subsequently converted to corresponding sesquiterpene alcohols by cytochrome P450 mediated hydroxylation at *cis* allylic methyl group of the side chain^17^,^25^.

In this manuscript, we present the transcriptome sequencing, cloning and functional characterization of genes, which encode the enzymes involved in sesquiterpene biosynthesis in Indian Sandalwood *S. album*. Based on the transcriptome screening, cloning and functional characterization of one prenyltransferase (SaFDS) and four sesquiterpene synthases, including sesquisabinene synthases (SaSQS1 and SaSQS2), bisabolene synthase (SaBS) and santalene synthase (SaSS) has been carried out. Although, sesquisabinene and its hydroxy derivative, sesquisabinene hydrate have been reported to be present in various natural sources^26^,^27^, to the best of our knowledge, there is no report on the isolation and functional characterization of the gene which encodes sesquisabinene synthase. Furthermore, we have also evaluated the feasibility of microbial production of sesquisabinene from both the unigenes in non-optimized bacterial cells and demonstrated the feasibility of metabolic engineering of SaSQS1 and SaSQS2 for preparative scale production of sesquisabinene in metabolically tractable heterologous systems.

## Results and Discussion

### RNA sequencing and *de novo* transcriptome assembly

The cDNA library of *S. album* was constructed using mRNA purified from total RNA isolated from the interface of heartwood and sapwood. The resultant cDNA library was amplified by PCR to enrich the adaptor ligated fragments and sequenced on one lane of the flow cell using paired end sequencing on Illumina GAII Analyzer. A total of 33,323,756 raw reads were generated with a length of 150 bp corresponding to 10.08 GB of sequence data file. Adapter trimming and low quality trimming was performed throughout the sequence to get better quality reads. High quality reads (>20 phred score) were then used for *de novo* assembly with varying hash lengths from 51 to 113. The 26,730,799 raw reads (Gen–Bank Accession: SRR1725543) (80.22%) obtained were assembled into 58,221 contigs with optimized hash length of 59, having an average contig length of 571.67 bp and an N50 value of 863. These contigs were submitted as inputs for Oasis_0.2.01 to generate 90,478 transcripts having N50 value of 695 and an average transcript length of 474.18 bp. These transcripts were further subjected to cluster and assembly analysis using CD–HIT to remove the redundancy, which resulted in a total of 84,094 unique transcripts with an average size of 494.17 bp and an N50 value of 717 containing 24,912 transcripts (29.62%) with lengths greater than 500 bp and 9,136 transcripts (10.86%) with lengths greater than 1 kb (Table 1 and Supplementary Fig. S2).

### Functional annotation

Functional annotation of unigenes provides valuable insights into genes, which are involved in specific molecular functions and biological processes. Various approaches for functional annotation of the assembled transcripts have been used to identify the genes, which are involved in terpenoid biosynthesis in *S. album*. All the 84,094 putative unigenes were compared with manually curated KEGG (Kyoto Encyclopedia of Genes and Genomes) database of *Arabidopsis thaliana* (Thale cress) and *Oryza sativa japonica* (Japanese rice) for functional annotation of genes by bidirectional BLAST^28^. KEGG Orthology (KO) numbers were assigned to 4,244 unigenes, representing 298 KEGG pathways involved in majority of plant biochemical pathways including metabolism, cellular processes and genetic information processing. There were 30 unigenes mapped specifically on enzymes involved in terpenoid backbone biosynthesis (Supplementary Fig. S4).

All the unique transcripts (84,094) were submitted to Virtual Ribosome-V1.1 to predict ORF of maximum length for each unigene in all six frames. A total of 83,823 unigenes (99.6%) were identified as having an ORF starting at the ATG codon, from which 17,119 unigenes (20.35%) contained the ORF of ≥ 100 amino acids length. To identify protein domain architecture, these 17,119 unigenes were submitted for Pfam analysis against PfamA database. Of 17,119 unigenes, only 10,668 could be assigned with Pfam IDs. Eighteen unigenes containing Pfam ID: PF01397 (terpene synthase N-terminal domain), PF03936 (terpene synthase family metal binding domain), PF00432 (prenyltransferase and squalene oxidase repeat), PF13243 (prenyltransferase like) were selected for screening of terpene synthases. The 10,668 transcripts were also submitted to megablastx search against NCBI Nr-database, Swissprot/ Uniprot database with an E-value ≤ 10^−5^, which resulted in 18 unigenes related to terpene synthases, 72 unigenes for CYP450 monooxygenase and 3 unigenes representing CYP450 reductases.

### Screening and isolation of terpene synthases

From transcriptome screening, five unigenes such as *SaFDS* (Locus_19031_Transcript_1/1_Confidence_1.000_length_453 bp), *SaSQS1* (Locus_33105_Transcript_1/1_Confidence_1.000_length_761 bp), *SaSQS2* (Locus_8408_Transcript_1/1_Confidence_1.000_length_1016 bp), *SaBS* (Locus_5558_Transcript_1/1_Confidence_1.000_length_832 bp) and *SaSS* (Locus_1838_Transcript_1/1_Confidence_1.000_length_438 bp) were identified using BLAST analysis, based on their homology with known terpene synthases reported in the NCBI database. EST fragment of *SaFDS* was found to match with FPP synthase reported from *Panax quinquefolius* with 88% identity at the amino acid level, but lacked its 5’ and 3’ sequences. The full-length cDNA sequence of SaFDS was obtained by performing 5’ and 3’ RACE reactions. The full length ORF of *SaFDS* (Gen-Bank Accession: KF011939) composed of 1029 bp encoding a polypeptide of 342 amino acids with a calculated molecular weight of 39.4 kDa and pI of 5.26. The deduced amino acid sequence of *SaFDS* showed resemblance with that of earlier reports^25^. The analysis of *SaFDS* amino acid sequence revealed the presence of several highly conserved regions including two aspartate rich motifs, FARM (L, X_4_L**DD**xx**D**xxxxRRG) and SARM (GxxFQxx**DD**xx**D**….GK) involved in binding of both homoallylic (IPP) and allylic diphosphate substrates (GPP and DMAPP)^29^,^30^,^31^.

The EST fragments of *SaSQS1* and *SaSQS2* lacked their 3’ sequences. The full-length cDNA sequence of both the unigenes were obtained by performing 3’ RACE. The ORFs of *SaSQS1* (Gen-Bank Accession: KJ665776) and *SaSQS2* (Gen-Bank Accession: KJ665777) composed of 1701 bp encoding a polypeptide of 566 amino acids with a calculated molecular weight of 65.22 kDa (SaSQS1), 65.44 kDa (SaSQS2) and pI of 5.01 (SaSQS1) and 5.10 (SaSQS2), respectively. Both the sequences shared a high level of similarity to each other with 81.7% identity at the nucleotide level and 82.8% identity and 89.6% similarity at protein sequence level (Supplementary Fig. S26). The deduced amino acid sequences of *SaSQS1* and *SaSQS2* were found to match with β-bisabolene synthase from *S. austrocaledonicum* with 63% identity at the amino acid level.

The missing 3’ end sequences of the EST fragment of *SaBS* was obtained by performing 3’ RACE. The ORF of *SaBS* (Gen-Bank Accession: KJ665778) was found to be composed of 1731 bp encoding a polypeptide of 576 amino acids with a calculated molecular weight of 65.90 kDa and pI of 5.48. Amino acid sequence analysis of *SaBS* with reported terpene synthases showed resemblance to monoterpene synthase (*SamonoTPS*) from *S. album* (99% identity), which was reported to produce traces of β-bisabolene with FPP^32^. Furthermore, there was no detailed study on biochemical characterization of β-bisabolene synthase from *S. album*.

The EST fragment of *SaSS* lacked its 5’ and 3’ sequences. To obtain the full-length cDNA sequence of *SaSS*, 5’ and 3’ RACE were performed. The ORF of *SaSS* (Gen-Bank Accession: KF011938) composed of 1710 bp encoding a polypeptide of 569 amino acids with a calculated molecular weight of 65.16 kDa and pI 5.63. The deduced amino acid sequence of *SaSS* was similar to that previously reported from *S. album*^25^.

Predicted polypeptide sequences of *SaSQS1*, *SaSQS2*, *SaBS* and *SaSS* lacked N-terminal organelle targeting sequence, suggesting that these enzymes are directed to the cytoplasm. The deduced protein sequences of these sesquiterpene synthases shared highly conserved residues with known sesquiterpene synthases including DDXXD motif^33^,^34^,^35^ involved in substrate binding, (D/**N**)DXX(S/**T**)XXX**E** motif^33^,^36^ essential for metal binding and also RRX_8_W motif^12^,^37^, the characteristic feature of TPS-b subfamily.

### Heterologous expression and functional characterization of SaFDS, SaSQS1, SaSQS2, SaBS and SaSS

The open reading frames of *SaFDS*, *SaSQS1*, *SaSQS2*, *SaBS* and *SaSS* were cloned in suitable vector frames with N terminal His_6_ tag for affinity purification under the control of T7-RNA polymerase promoter for expression of soluble active protein in *E. coli* BL21 DE3 or Rosetta 2 DE3 cells (Supplementary Table S2). Recombinant His_6_-tagged proteins were purified to homogeneity by Ni^2+^-affinity chromatography with a yield of 10-30 mg/L of bacterial culture.

**Figure 2:**
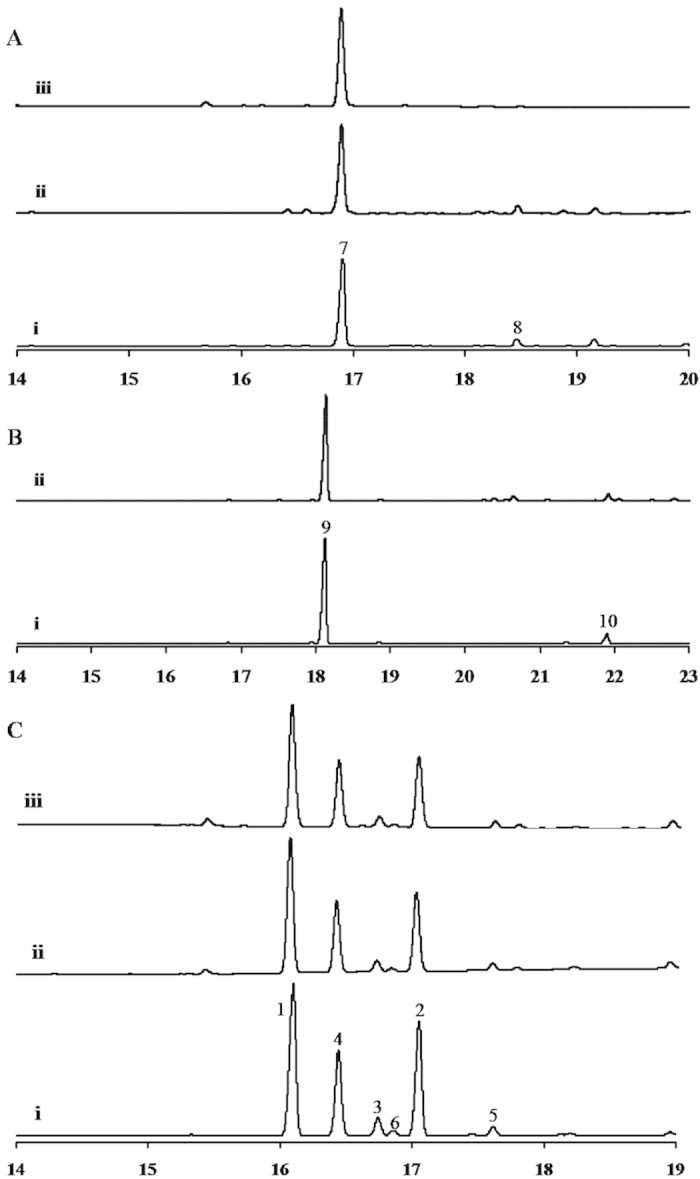
GC-FID chromatogram for the assay extracts using HP-5 capillary column (30m × 0.32 mm × 0.25 μm) (Supplemental Table S5 program 1): **A**) (i) Sesquisabinene synthase1 (SaSQS1) with (*E,E*)-FPP, (ii) Sesquisabinene synthase (SaSQS2) with (*E,E*)-FPP, (iii) Purified enzymatic product sesquisabinene (7). **B**) (i) Bisabolene synthase (SaBS) with FPP producing (S)-β-bisabolene (9), α-bisabolol (10), (ii) co-injection of (*S*)-β-bisabolene and assay product of SaBS with FPP. **C**) Santalene synthase (SaSS) with (i) FPP, (ii) GPP + IPP and farnesyl diphosphate synthase SaFDS, (iii) DMAPP + (2 × IPP) and SaFDS, α-santalene (**1**), β-santalene (****2****), *epi*-β-santalene (**3**), *exo*-α-bergamotene (**4**), *exo*-β-bergamotene (**5**), (E)-β-farnesene (**6**).

**Figure 3:**
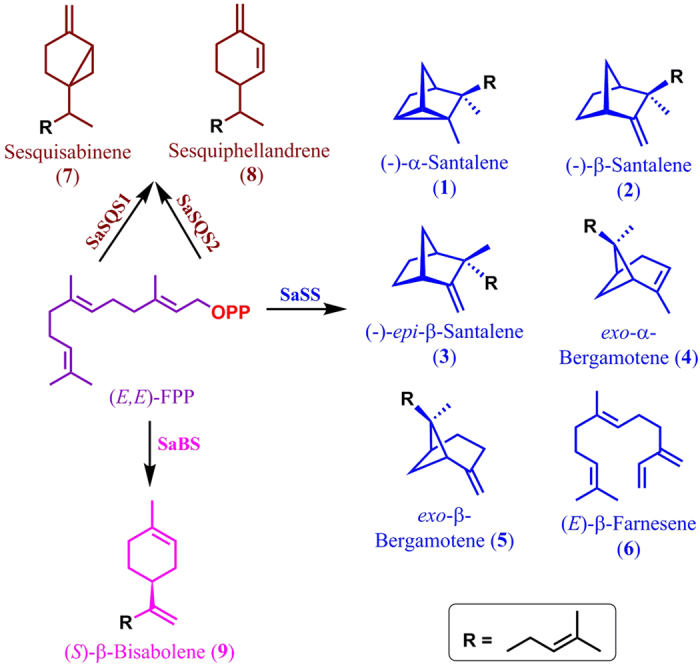
Products of sesquiterpene synthases, Santalene synthase (SaSS), Sesquisabinene synthase (SaSQS1 and SaSQS2) and bisabolene synthase (SaBS) with (*E*,*E*)-FPP as a substrate.

Incubation of recombinant SaFDS with equimolar concentrations of IPP and GPP or 1:2 molar ratio of DMAPP and IPP resulted in the formation of (*E,E*)-FPP, which on subsequent treatment with alkaline phosphatase yielded the hydrolyzed product (*E,E*)-farnesol. The product from both the reactions was characterized as (*E,E*)-farnesol by GC and GC-MS analyses and co-injection studies using standard (*E,E*)-farnesol (Supplementary Fig. S27). To assess the function of recombinant protein of SaSQS1 and SaSQS2, enzyme assays were performed using purified protein with (*E,E*)-FPP as substrate in the presence of divalent cation Mg^2+^. GC-MS analyses of the assay extracts indicated that both the enzymes were able to produce a sesquiterpene hydrocarbon (**7**) with *m/z* 204 as an exclusive (>93%) enzymatic product, with traces of β-sesquiphellandrene (**8**) (~5%) and an unidentified metabolite (~2%) (Fig. 2a, and Supplementary Figs. S35-S38). To characterize the enzymatic product (**7**), large scale assays were performed using 80 mg of purified SaSQS1 protein with 60 mg of (*E,E*)-FPP (Method S1.4). The hexane extract of the assay mixture was subjected to silica gel column chromatography to obtain the pure product. Based on the spectral data, the enzymatic product was identified as sesquisabinene (**7**) and the data was found to match well with that of its earlier report^38^. GC-MS analyses and GC co-injection studies using Astec CHIRAL DEX^TM^ B-DA chiral capillary column (Supplementary Table S4 program2) clearly indicated that both SaSQS1 and SaSQS2 enzymes catalyzed the cyclization of (*E*,*E*)-FPP to sesquisabinene in presence of Mg^2+^(Fig. 3 and Supplementary Fig. S28). Incubation of recombinant SaBS with FPP produced β-bisabolene (**9**) as a major product (92%) with the solvated product α-bisabolol (**10**) contributing to the rest of the assay product (8%). The enzymatic products were confirmed by GC-MS fragmentation analysis and GC co-injection studies with authentic standards (Fig. 2b and Supplementary Figs. S46-S49 and S66). GC analysis using HP-chiral (20% β-cyclodextrin) capillary column^39^ clearly indicated the enzymatic product to be a single enantiomer, which was characterized as (*S*)-β-bisabolene (Fig. 3 and Supplementary Fig. S29).

Further, incubation of SaSQS1 with GPP resulted in the formation of β-pinene (**13**, 9.4%), myrcene (**14**, 13.4%), linalool (**15**, 34.1%) and α-terpineol (**16**, 28.6%), along with two uncharacterized monoterpenes (**11**, 6.4%, and **12**, 8.1%, respectively). GC and GC-MS analyses (Supplementary Table S4 program 3) of the assay extract of SaSQS1 with neryl diphosphate (NPP) indicated the formation of similar set of monoterpenes along with solvated product, nerol as one of the products. SaSQS2 also produced the same metabolites when incubated with GPP and NPP (Supplementary Fig. S31). On the other side, incubation of SaBS with GPP and NPP resulted in the formation of α-terpineol as a major metabolite, with traces of linalool (Supplementary Fig. S32). GC analyses of assay extracts of SaSQS1 and SaBS with GPP using HP-chiral (20% β-cyclodextrin, Supplementary Table S4 program 4) capillary column revealed that SaSQS1 produces a racemic mixture of linalool and α-terpineol (Supplementary Fig. S33), whereas SaBS forms a single isomer of α-terpineol (Supplementary Fig. S34).

For the functional characterization of SaSS, enzyme assay was performed using purified recombinant protein with (*E,E*)-FPP in the presence of Mg^2+^. GC and GC-MS analyses of the assay extracts indicated the presence of six compounds (Fig. 2c, R_t_: 16.1, 16.4, 16.7, 16.8, 17.0 and 17.6 min, Supplementary Table S4 program 1). GC and GC-MS profiles of product ratios were in the similar range as those of the earlier report on SaSS^25^. Four metabolites eluting at 16.1, 16.4, 16.7 and 17.0 min were identified as α-santalene (**1**, 41.2 ± 1.0%), β-santalene (**2**, 29.5 ± 0.4%), *epi*-β-santalene (**3**, 4.4 ± 0.0%) and *exo*-α-bergamotene (**4**, 21.6 ± 0.6%), respectively, by comparing the retention time, mass fragmentation pattern and co-injection studies with those of purified compounds (Fig. 3 and Supplementary Figs. S39-S45, S65)^17^. On comparing the mass fragmentation pattern of the compounds eluting at 16.8 min and 17.6 min with NIST/Wiley mass spectral library, they were found to match with farnesenes^25^. However, when the SaSS assay extract was co-injected with the synthesized (*E*)-β-farnesene (**6**) and farnesene mixture^40^, only one sesquiterpene at R_t_ 16.8 min co-eluted with synthesized (*E*)-β-farnesene, whereas the peak corresponding to R_t_17.6 min did not match with any of the synthesized farnesenes (Supplementary Fig. S65 H-I). Surprisingly, a mutant of SaSS (Y539W, NCBI: JQ690659) was able to cyclize (*E*,*E*)-FPP into the compound with R_t_ 17.6 min (Supplementary Table S4 program 1) as one of the major compounds (data not shown). This compound was purified and characterized as *exo*-β-bergamotene (**5**) by comparing the spectral data with that of the earlier report^41^. GC and GC-MS co-injection studies using authentic compound **5**, the SaSS catalysed reaction product eluting at 17.6 min was identified as *exo*-β-bergamotene (**5**) (Supplementary Fig. S65 J-K).

All these sesquiterpenes (**1**-**9**) (Fig. 3) are formed through carbocationic cascade reactions and intra-molecular cyclizations involving Wagner-Meerwein rearrangements of the farnesyl carbocation. Interestingly, when IPP and DMAPP/GPP were sequentially incubated with SaFDS and SaSS (methods section), the ratio of the sesquiterpenes formed (**1**-**6**) were in the same range as observed by incubating (*E,E*)-FPP with SaSS (Fig. 2c). Similarly, incubation of IPP and DMAPP/GPP in presence of SaFDS and SaSQS1/SaSQS2/SaBS, led to the formation of respective sesquiterpenes, sesquisabinene/ bisabolene (Supplementary Figs. S30 I-II). These results clearly indicate that the combined assay strategy utilizing sequential catalysis by SaFDS and SaSS/SaSQS1/SaBS along with appropriately deuterium labelled IPP and/or DMAPP/GPP can be utilized to gain insights into the mechanisms involved in the biosynthesis of corresponding sesquiterpenes.

### *In vivo* production of sesquisabinene in microbial host

**Figure 4:**
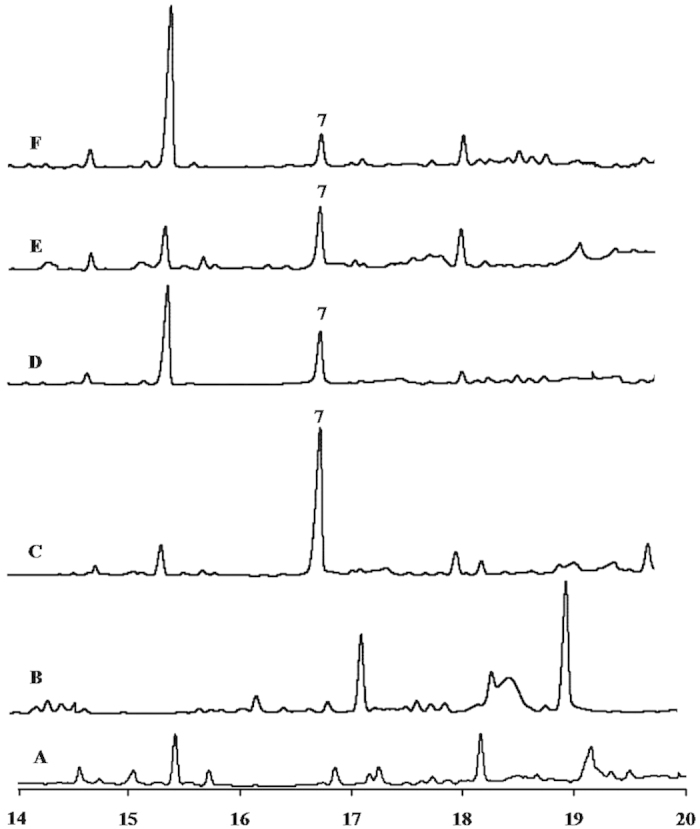
Total ion chromatograms of *in vivo* productions of SaSQS1 and SaSQS2, **A**) n-hexane extract of supernatant of empty vector control, **B**) n-hexane extract of pellet of empty vector, **C**) n-hexane extract of supernatant of SaSQS1, **D**) n-hexane extract of pellet of SaSQS1, **E**) n-hexane extract of supernatant of SaSQS2, **F**) n-hexane extract of pellet of SaSQS2.

Microbial production of sesquisabinene with both sesquiterpene synthases (*SaSQS1* and *SaSQS2*) using *in vivo* expression system: pETDuet-1:SaFDS:SaSQS1/SaSQS2 was performed in C41DE3 cells containing pRARE plasmid. GC and GC-MS analyses of both the cell pellet and broth extracts indicated the presence of sesquisabinene (**7**) in bacterial cells harbouring *SaSQS1* and *SaSQS2* where as **7** was not detected in bacterial culture containing empty vector. Using the standard curve drawn for sesquisabinene, GC-FID quantification of **7** was carried out under similar conditions for both pellet and broth extracts. Sesquisabinene production by SaSQS1 was found to be ~3 mg/L of bacterial culture, whereas yield of sesquisabinene was 1 mg/L for the bacterial culture of SaSQS2 (Fig. 4). Trace of **7** was detected in pellet extracts. The difference in the yields of sesquisabinene from both the synthases correlated well with the kinetic parameters of SaSQS1 and SaSQS2 (Table 2).

### Phylogenetic analysis

**Figure 5:**
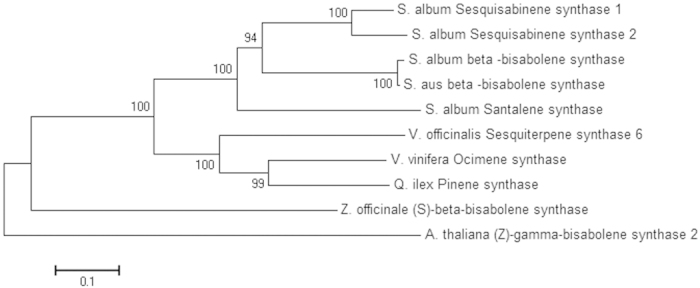
Phylogenetic analysis of terpene synthases isolated from *S. album*, Sequence used for phylogenetic tree construction are: *S. album* Sesquisabinene synthase 1 (SaSQS1, KJ665776), *S. album* Sesquisabinene synthase 2 (SaSQS2, KJ665777), *S. album* beta-bisabolene synthase (KJ665778), *S. aus* beta -bisabolene synthase (ADO87003), *S. album*, Santalene synthase (KF011938), *V. officinalis* Sesquiterpene synthase 6(AGB05615), *V. vinifera*, Ocimene synthase (ADR74206), *Q. ilex* Pinene synthase (CAK55186), *Z. officinale* (*S*)-beta-bisabolene synthase (BAI67934), *A. thaliana* (Z)-gamma-bisabolene synthase 2 (NP_193066).

A neighbour joining phylogenic tree placed all the full-length sequences of sesquiterpene synthases isolated from *S. album* in a separate clade. Santalene synthase (SaSS), a moderately promiscuous enzyme, branches out separately with the other sesquiterpene synthases (SaSQS1, SaSQS2 and SaBS), which demonstrate high fidelity, while forming dominant single products. The other branch containing SaSQS1, SaSQS2 and SaBS diverges into two nodes, one representing β-bisabolene synthase (SaBS) and the other separated into two clades representing sesquisabinene synthases (SaSQS1 and SaSQS2), respectively (Fig. 5). Phylogenetic analysis suggested that all the sesquiterpene synthases in *S. album* are evolved from a common ancestor more closely related to monoterpene synthases.

### Molecular regulation of sesquiterpene biosynthesis in *Santalum album*

**Figure 6:**
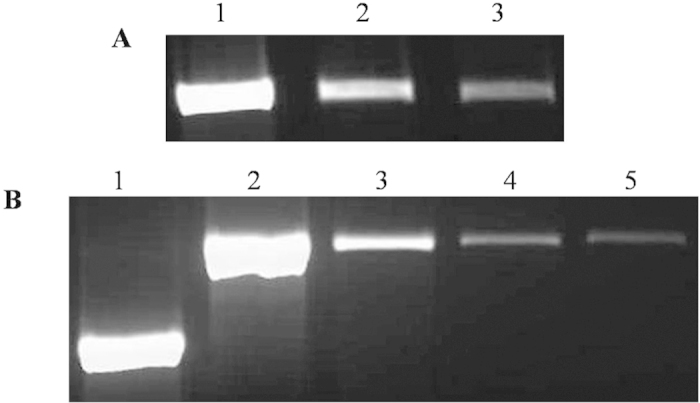
Semi-quantitative real time PCR gel image representing relative transcript level of sesquiterpene synthases present at interface of heartwood and sapwood, **A**) 18S rRNA semi-qPCR for 30 cycle, **Lane 1:** undiluted cDNA, **Lane 2:** 1:5 diluted cDNA (20 ng), **Lane 3:** 1:10 diluted cDNA (10 ng), **B**) *SaFDS*, *SaSS*, *SaSQS1*, *SaSQS2*, and *SaBS* semi- qPCR with 1:5 diluted cDNA (20 ng) for 32 cycle, **Lane 1:** semi-qPCR of *SaFDS*, **Lane 2:** semi-qPCR of *SaSS*, **Lane 3:** semi-qPCR of *SaSQS1*, **Lane 4:** semi-qPCR of *SaSQS2*, **Lane 5:** semi-qPCR of *SaBS*.

What exactly triggers the formation of santalene derivatives with extremely high levels as compared to other sesquiterpenes in sandalwood oil is unknown till date. Previous reports state higher expression of SaFDS and SaSS in matured wood, but no comparative data is available for expression levels of other sesquiterpene synthases present at the interface of heartwood and sapwood of *S. album*^42^. In order to establish the molecular regulation of sesquiterpene biosynthesis, kinetic parameters and expression levels of all the sesquiterpene synthases have been determined. Steady-state kinetic constants for sesquiterpene synthases were evaluated for FPP and observed that SaSS had very low K_m_ (0.6 μM) and an exceptionally high kinetic efficiency (>50–100 fold) in comparison to other sesquiterpene synthases (Table 2). Comparative expression level analysis of all the characterized sesquiterpene synthases by semi-quantitative real time PCR revealed substantially higher expression level of *SaSS* and their relative abundance was compared with *SaFDS* (Fig. 6). However, expression level of *SaSQS1* was found to be approximately 2-3 times higher than *SaSQS2* and that of *SaBS* was very low in comparison to other sesquiterpene synthases. The pattern of kinetic constants and expression levels of sesquiterpene synthases are in correlation to the sesquiterpene composition in sandalwood essential oil (Supplementary Fig. S70). These results suggest robust kinetic parameters and very high expression level of *SaSS* as compared to other sesquiterpene synthases (*SaSQS1*, *SaSQS2* and *SaBS*) leading to the biosynthesis of santalene mixtures in much higher amount even at lower cellular concentrations of (*E,E*)-FPP, which are subsequently hydroxylated by mono-oxygenase system/s to generate respective sesquiterpene alcohols.

## Concluding Remarks

Based on the transcriptome sequencing, we have isolated and functionally characterized one prenyltransferase (SaFDS) and two classes of sesquiterpene synthases. One class represents santalene synthase (SaSS), a multi product-forming enzyme, whereas the second class of sesquiterpene synthases represents, sesquisabinene synthases (SaSQS1 and SaSQS2) and β-bisabolene synthase (SaBS), which form the dominant single products. The products formed were characterized based on thorough spectral analysis, comparison of the mass fragmentation patterns and co-injection studies using authentic standards. The pattern of kinetic constants and expression levels of sesquiterpene synthases were found to be in correlation to the sesquiterpene composition in sandalwood essential oil^17^. Molecular and biochemical characterization of four sesquiterpene synthases revealed that robust kinetic parameters and very high expression level of SaSS as compared to SaSQS1, SaSQS2 and SaBS could lead to the formation of santalene mixtures in a much higher level, which are further hydroxylated by CYP450 system to generate respective sesquiterpene alcohols. Functional characterization of SaSQS1 and SaSQS2 revealed that both the enzymes, exhibiting different kinetic parameters, catalyzed an exclusive formation of sesquisabinene from (*E,E*)-FPP. Furthermore, production of sesquisabinene in heterologous bacterial system was validated by co-expressing SaFDS and SaSQS1 or SaSQS2. These results may pave the way for the large-scale production of these sesquiterpenes in metabolically tractable heterologous systems.

## Methods

### Plant material

Wood shavings from the interface of heartwood and sapwood were collected at a height of 30–40 cm from ground level from mature sandalwood trees at CSIR–NCL campus, Pune, using Hagloff wood borer, flash-frozen in liquid nitrogen and stored at −80 °C until further use.

### RNA isolation and transcriptome sequencing

Total RNA was isolated from the interface of heartwood and sapwood of Indian Sandalwood using a protocol initially reported for isolation of RNA from xylem tissue^43^ with extensive modifications (Supplementary Method S1.1). Transcriptome library for sequencing was constructed according to the Illumina TruSeq RNA library protocol outlined in “TruSeq RNA Sample Preparation Guide” at Genotypic Technology Bangalore, India. The library was amplified using 8 cycles of PCR for enrichment of adapter-ligated fragments. Primary QC check of the raw data was performed using the inbuilt tool SeqQC-V2.1.

### Transcriptome assembly and functional annotation

To obtain high quality clean read data for *De novo* assembly, the raw reads were filtered by discarding the reads containing adaptor sequence and poor quality reads (Phred score < 20). The clean reads were first assembled into contigs using the Velvet_1.1.05. Assembled contigs were given as input for Oasis_0.2.01 to generate transcripts. Redundancy in the transcripts was removed using CD-HIT. To assign molecular function, biological processes and cellular components of transcript, functional annotation of unigenes were performed using KEGG-KAAS analysis, Pfam domain analysis and megablastx search against NCBI Nr database, SwissProt/Uniprot database, Protein Data Bank (PDB) with an E-value ≤ 10^−5^.

### Isolation and cloning of terpene synthases in expression vector

Coding sequences of prenyltransferase (*SaFDS*) and sesquiterpene synthases (*SaSQS1*, *SaSQS2*, *SaSS* and *SaBS*) were amplified from cDNA using full-length ORF primers having RE site at both the ends (Supplementary Table S1 and S2). PCR was performed using Proof reading taq DNA polymerase (Sigma-Aldrich). PCR products purified from agarose gel were digested with respective restriction enzymes (NEB) incorporated at their ends and ligated with their expression vectors (pRSETB, pET32b and pET28a, respectively).

### Bacterial expression and purification of active protein of SaFDS,SaSS, SaSQS1, SaSQS2 and SaBS

For the expression of active protein, recombinant plasmids such as pRSETB harbouring *SaFDS* was introduced into BL21 DE3 competent cells, whereas, pET32b harbouring coding sequence of *SaSS* and pET28a harbouring coding sequence of *SaSQS1*, *SaSQS2* and *SaBS* were introduced in Rosetta 2 DE3 competent cells. Recombinant protein was expressed under the control of IPTG induction in respective cells and protein was purified to the homogeneity using Ni-affinity column chromatography (Supplementary Method S1.3). Protein concentrations were determined using Bradford method^44^ and all the fractions were analyzed on 10% SDS–PAGE.

### Product ratio studies of sesquiterpene synthases

The assay mixture contained purified recombinant protein (100 μg) in buffer (25 mM HEPES, 10% v/v glycerol, 5 mM dithiothreitol, 10 mM MgCl_2_, pH 7.4) with isoprenyl diphosphate as substrates (100 μM) in a final reaction volume of 400 μL. SaFDS was assayed using IPP and allylic diphosphate (GPP/DMAPP) as the substrates. The assay mixture was incubated at 30 °C on a rotary shaker for 1 h. After this incubation period, alkaline phosphatase (80 units dissolved in glycine buffer, pH 10.5) was added and incubation was continued at 37 °C on a rotary shaker (150 rpm). After 1 h of incubation, the assay mixture was cooled to 4 °C and extracted with n-hexane (3 × 500 μL). The pooled organic layer was dried over anhydrous Na_2_SO_4_, reduced to ~50 μL with a stream of dry nitrogen and analyzed by GC and GC-MS. Sesquiterpene synthases (SaSS, SaSQS1, SaSQS2, SaBS) were assayed using (*E,E*)-FPP as the substrate by incubating at 30 °C on a rotary shaker for 2 h^25^,^45^. After this incubation period, all the reaction mixtures were extracted with n-hexane (3 × 500 μL). The organic layers containing sesquiterpene products were dried over anhydrous Na_2_SO_4_ and reduced to ~50 μL with a stream of dry nitrogen. The extracts were analyzed by GC and GC-MS and the sesquiterpene products formed were identified by co-injection with purified or authentic compounds, by comparing the retention time and mass fragmentation. In the combined assays of SaFDS and the sesquiterpene synthases, the assay mixture containing SaFDS along with IPP and allylic diphosphates (DMAPP/GPP) (100 μM) were incubated for 1 h at 30 °C on a rotary shaker. After this incubation period, 100 μg of recombinant sesquiterpene synthases (SaSS/SaSQS1/SaSQS2/SaBS) were added and the incubation was continued for another 2 h. Further, the contents were extracted and products were analyzed using GC, GC-MS and GC-QTOF (Supplementary Table S4).

### Kinetic characterization of sesquiterpene synthases

Steady state kinetics of SaSS, SaSQS1, SaSQS2 and SaBS were performed using 5 μM proteins with varying substrate concentrations, ranging from 0.5 μM to 60 μM, in HEPES buffer in a reaction volume of 500 μL for 5 min at 30 °C and 150 rpm. Reactions were quenched by adding saturated Ba(OH)_2_ (250 μL) and 0.1 M ZnSO_4_ (250 μL) followed by vigorous vortexing. Reaction mixtures were extracted with n-hexane (3 × 1 mL). The extracts were supplemented with 100 ng of dodecane as an internal standard and concentrated to 50 μL with a stream of dry nitrogen gas. The product ratios were analyzed using GC and the calculated amount of products formed was used for the determination of apparent K_m_, K_cat_ and K_cat_/K_m_ values using Graph pad Prism software.

### Phylogenetic analysis

To determine the evolutionary origin of all the four sesquiterpene synthases, phylogenetic tree was constructed. Multiple sequence alignment was performed using ClustalX 2.1 software and phylogenetic tree was generated using the nearest neighbour joining method though ClustalX 2.1 and visualized using Mega6 software^46^.

### *In vivo* production of sesquisabinene

To validate the microbial production of sesquisabinene, *SaFDS*, *SaSQS1* and *SaSQS2* were sub-cloned in pETDuet-1 vector (supplemental method S1.5). Dual expression vector construct pETDuet-1-*SaFDS*:*SaSQS1*/*SaSQS2* as well as empty vector control were introduced into C41 DE3 cells containing pRARE plasmid. Individual colonies were inoculated in 5 mL of LB containing 100 μg/mL ampicillin and 34 μg/mL chloramphenicol and incubated overnight at 37 °C. Overnight grown cultures were transferred to 100 mL of Terrific Broth containing the same antibiotics and incubated at 37 °C till absorbance at 600 nm reached 0.8. Cultures were then equilibrated at 20 °C for 1 h and then induced with 0.5 mM IPTG and further incubated at same temperature for 14–16 h, after which the cultures were harvested by centrifugation at 10000 × *g* for 10 minutes. The supernatants were extracted twice with 100 mL of n-hexane each time, whereas the pellets were lysed by alkaline lysis (0.2 M NaOH) and extracted with n–hexane. Extracted samples were concentrated and analysed by GC/GC-MS using the conditions described earlier.

### Semi-quantitative Real time analysis

Two μg of total RNA was used for the synthesis of cDNA using Superscript III cDNA synthesis kit (Invitrogen) according to the manufacturer’s instructions. PCR was carried out in duplicates in a total volume of 10 μL reaction scale using Jump-start Taq DNA polymerase (Sigma-Aldrich). PCR was setup using 10 μM of forward and reverse primers of SaFDS, SaSS, SaSQS1, SaSQS2 and SaBS along with 18S rRNA forward and reverse primers (Supplementary Table S3) with 20 ng of cDNA at PCR program: 95 °C for 2 min, followed by 32 cycles at 95 °C for 20 sec, 59 °C for 20 sec, 72 °C for 1 min 50 sec. All the samples were analyzed on 1.5% agarose gel electrophoresis.

## Author Contributions

Isolation of genes and cloning was done by P.L.S.; Protein purification was done by P.L.S., R.K. and S.S.S.; A.P. and P.L.S have contributed in transcriptome analysis; enzymatic characterization and kinetics was performed by P.L.S., P.D. and R.K.; H.V.T. and P.L.S. analyzed the data; H.V.T. has conceived the project, designed the experiments and written the manuscript.

## Additional Information

**How to cite this article**: Srivastava, P. L. *et al*. Functional Characterization of Novel Sesquiterpene Synthases from Indian Sandalwood, *Santalum album*. *Sci. Rep.* **5**, 10095; doi: 10.1038/srep10095 (2015).

## Supplementary Material

Supplementary Information

## Figures and Tables

**Figure 1:**
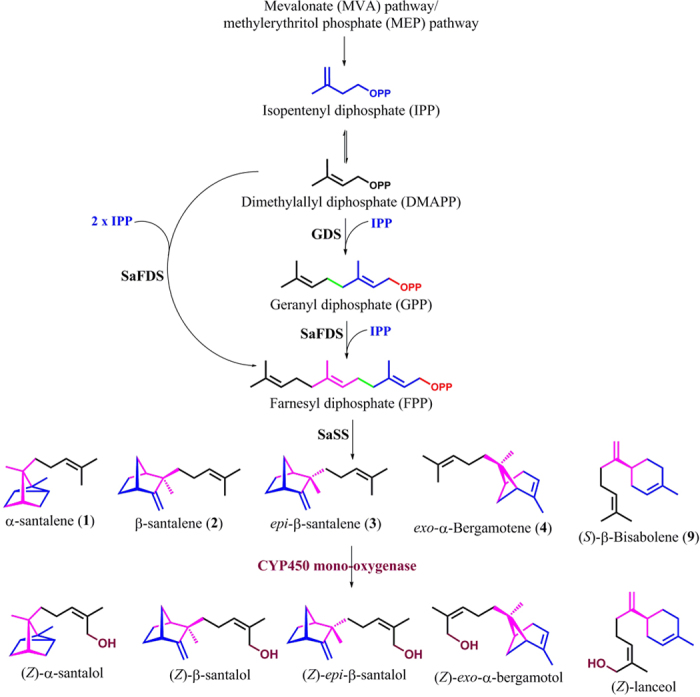
Schematic representation of proposed biosynthetic pathway for santalenes and santalols in Indian Sandalwood *S. album*; **GDS**: Geranyl diphosphate synthase, **SaFDS**: Farnesyl diphosphate synthase, **SaSS**: Santalene synthase, **CYP450**: Cytochrome P450.

**Table 1:** Transcripts’ assembly statistics.

**Velvet_1.1.05 (Oases 0.2.01) Transcripts Statistics**			
**Description**	**Contigs**	**Total transcript**	**Unigenes**
Hash length	59	59	55
Transcripts Generated	58221	90478	84094
Maximum Transcript Length	11726	12279	12279
Minimum Transcript Length	117	100	100
Average Transcript Length	571.671	474.18	494.17
Total Transcripts Length	33283265	42902769	41557227
Total Number of Non-ATGC Characters	20	120683	112096
Percentage of Non-ATGC Characters	6.00903e-07	0.00281294	0.00269739
Transcripts>100 bp	58221	90476	84092
Transcripts>500 bp	21371	25145	24912
Transcripts>1 Kb	8779	9187	9136
Transcripts>10 Kb	1	3	3
Transcripts>1 Mb	0	0	0
N50 value	863	695	717
Number of reads assembled	26730799	29886230	
Total number of reads	33323756	33323756	
Percentage of reads assembled	80.2154445	89.68445814	

**Table 2:** Kinetic parameters of sesquiterpene synthases isolated from Sandalwood.

**Enzymes**	**K**_**m**_ **(μM)**	**K**_**cat**_**(min**^**−1**^)	**K**_**cat**_**/K**_**m**_ **(M**^**−1**^ **min**^**−1**^)
SaSS	0.59 ± 0.24	0.50 ± 0.03	0.9×10^6^
SaSQS1	11.12 ± 2.80	0.35 ± 0.03	0.3×10^5^
SaSQS2	15.30 ± 3.83	0.14 ± 0.01	0.9×10^4^
SaBS	12.59 ± 1.40	0.04 ± 0.00	0.3×10^4^

## References

[b1] Chen M. *et al.* Mechanistic Insights from the Binding of Substrate and Carbocation Intermediate Analogues to Aristolochene Synthase. Biochemistry 52, 5441–5453 (2013). 23905850 10.1021/bi400691v PMC3755762

[b2] Rock C. D. & Zeevaart J. A. D. The aba mutant of Arabidopsis thaliana is impaired in epoxy-carotenoid biosynthesis. Proc. Natl. Acad. Sci. USA 88, 7496–7499 (1991). 11607209 10.1073/pnas.88.17.7496 PMC52327

[b3] Beyer P., Mayer M. & Kleinig H. Molecular oxygen and the state of geometric isomerism of intermediates are essential in the carotene desaturation and cyclization reactions in daffodil chromoplasts. Eur. J. Biochem. 184, 141–150 (1989). 2776764 10.1111/j.1432-1033.1989.tb15000.x

[b4] Trumpower Bl, Houser R. M. & Olson R. E. Studies on ubiquinone. Demonstration of the total biosynthesis of ubiquinone-9 in rat liver mitochondria. J. Biol. Chem. 249, 3041–3048 (1974). 4378394

[b5] Anderson M. S., Yarger J. G., Burck C. L. & Poulter C. D. Farnesyl diphosphate synthetase. Molecular cloning, sequence, and expression of an essential gene from Saccharomyces cerevisiae. J. Biol. Chem. 264, 19176–19184 (1989). 2681213

[b6] Ding V. D. H. *et al.* Purification and characterization of recombinant human farnesyl diphosphate synthase expressed in Escherichia coli. Biochem. J. 275, 61–65 (1991). 2018485 10.1042/bj2750061 PMC1150013

[b7] Lange B. M., Rujan T., Martin W. & Croteau R. Isoprenoid biosynthesis: The evolution of two ancient and distinct pathways across genomes. Proc. Natl. Acad. Sci. USA 97, 13172–13177 (2000). 11078528 10.1073/pnas.240454797 PMC27197

[b8] McCaskill D. & Croteau R. Prospects for the bioengineering of isoprenoid biosynthesis. Adv. Biochem. Eng. Biot. 55, 107–46 (1997). 10.1007/BFb0102064 9017926

[b9] Kellogg B. A. & Poulter C. D. Chain elongation in the isoprenoid biosynthetic pathway. Curr. Opin. Chem. Biol. 1, 570–578 (1997). 9667899 10.1016/s1367-5931(97)80054-3

[b10] Cane D. E. Isoprenoid biosynthesis. Stereochemistry of the cyclization of allylic pyrophosphates. Acc. Chem. Res. 18, 220–226 (1985).

[b11] Croteau R. Biosynthesis and catabolism of monoterpenoids. Chem. Rev. 87, 929–954 (1987).

[b12] Bohlmann J., Meyer-Gauen G. & Croteau R. Plant terpenoid synthases: Molecular biology and phylogenetic analysis. Proc. Natl. Acad. Sci. USA 95, 4126–4133 (1998). 9539701 10.1073/pnas.95.8.4126 PMC22453

[b13] Bommareddy A., Rule B., VanWert A. L., Santha S. & Dwivedi C. α-Santalol, a derivative of sandalwood oil, induces apoptosis in human prostate cancer cells by causing caspase-3 activation. Phytomedicine 19, 804–811 (2012). 22571975 10.1016/j.phymed.2012.04.003

[b14] Klayman D. L. Qinghaosu (artemisinin): an antimalarial drug from China. Science 228, 1049–1055 (1985). 3887571 10.1126/science.3887571

[b15] Rude M. A. & Schirmer A. New microbial fuels: a biotech perspective. Curr. Opin. Microbiol. 12, 274–281 (2009). 19447673 10.1016/j.mib.2009.04.004

[b16] Peralta-Yahya P. P. & Keasling J. D. Advanced biofuel production in microbes. Biotechnol. J. 5, 147–162 (2011). 20084640 10.1002/biot.200900220

[b17] Daramwar P. P., Srivastava P. L., Priyadarshini B. & Thulasiram H. V. Preparative separation of α- and β-santalenes and (*Z*)-α- and (*Z*)-β-santalols using silver nitrate-impregnated silica gel medium pressure liquid chromatography and analysis of sandalwood oil. Analyst 137, 4564–4570 (2012). 22900258 10.1039/c2an35575b

[b18] Baldovini N., Delasalle C. & Joulain D. Phytochemistry of the heartwood from fragrant Santalum species: a review. Flavour Frag. J. 26, 7–26 (2011).

[b19] Jones C. G., Ghisalberti E. L., Plummer J. A. & Barbour E. L. Quantitative co-occurrence of sesquiterpenes; a tool for elucidating their biosynthesis in Indian sandalwood, Santalum album. Phytochemistry 67, 2463–2468 (2006). 17045624 10.1016/j.phytochem.2006.09.013

[b20] Harbaugh D. T. & Baldwin B. G. Phylogeny and biogeography of the sandalwoods (Santalum, Santalaceae): repeated dispersals throughout the Pacific. Am. J. Bot. 94, 1028–1040 (2007). 21636472 10.3732/ajb.94.6.1028

[b21] Page T. *et al.* Geographic and Phenotypic Variation in Heartwood and Essential-Oil Characters in Natural Populations of Santalum austrocaledonicum in Vanuatu. Chem. Biodivers. 7, 1990–2006 (2010). 20730962 10.1002/cbdv.200900382

[b22] Jirovetz L. *et al.* Comparative study on the antimicrobial activities of different sandalwood essential oils of various origin. Flavour Frag. J. 21, 465–468 (2006).

[b23] Dwivedi C., Maydew E. R., Hora J. J., Ramaeker D. M. & Guan X. M. Chemopreventive effects of various concentrations of alpha-santalol on skin cancer development in CD-1 mice. Eur. J. Cancer Prev. 14, 473–476 (2005). 16175052 10.1097/01.cej.0000178075.20124.2a

[b24] Hongratanaworakit T. Stimulating Effect of Aromatherapy Massage with Jasmine Oil. Nat. Prod. Commun. 5, 157–162 (2010). 20184043

[b25] Jones C. G. *et al.* Sandalwood Fragrance Biosynthesis Involves Sesquiterpene Synthases of Both the Terpene Synthase (TPS)-a and TPS-b Subfamilies, including Santalene Synthases. J. Biol. Chem. 286, 17445–17454 (2011). 21454632 10.1074/jbc.M111.231787 PMC3093818

[b26] Radulovic N. S., Dordevic N. D., Zlatkovic B. K. & Palic R. M. Composition of the essential oil of Geocaryum cynapioides (Guss.) L. Engstrand. Chem. Pap. 62, 603–607 (2008).

[b27] Sonboli A. *et al.* Chemotaxonomic importance of the essential-oil composition in two subspecies of Teucrium stocksianum Boiss. from Iran. Chem. Biodivers. 10, 687–694 (2013). 23576354 10.1002/cbdv.201200088

[b28] Moriya Y., Itoh M., Okuda S., Yoshizawa A. C. & Kanehisa M. KAAS: an automatic genome annotation and pathway reconstruction server. Nucleic Acids Res. 35, W182–W185 (2007). 17526522 10.1093/nar/gkm321 PMC1933193

[b29] Marrero P. F., Poulter C. D. & Edwards P. A. Effects of site-directed mutagenesis of the highly conserved aspartate residues in domain II of farnesyl diphosphate synthase activity. J. Biol. Chem. 267, 21873–21878 (1992). 1400496

[b30] Thulasiram H. V., Erickson H. K. & Poulter C. D. Chimeras of two isoprenoid synthases catalyze all four coupling reactions in isoprenoid biosynthesis. Science 316, 73–76 (2007). 17412950 10.1126/science.1137786

[b31] Song L. S. & Poulter C. D. Yeast farnesyl-diphosphate synthase: site-directed mutagenesis of residues in highly conserved prenyltransferase domains I and II. Proc. Natl. Acad. Sci. USA 91, 3044–3048 (1994). 8159703 10.1073/pnas.91.8.3044 PMC43511

[b32] Jones C. G. *et al.* Isolation of cDNAs and functional characterisation of two multi-product terpene synthase enzymes from sandalwood, Santalum album L. Arch. Biochem. Biophys. 477, 121–130 (2008). 18541135 10.1016/j.abb.2008.05.008

[b33] Starks C. M., Back K., Chappell J. & Noel J. P. Structural basis for cyclic terpene biosynthesis by tobacco 5-*epi*-aristolochene synthase. Science 277, 1815–1820 (1997). 9295271 10.1126/science.277.5333.1815

[b34] Lesburg C. A., Zhai G., Cane D. E. & Christianson D. W. Crystal structure of pentalenene synthase: mechanistic insights on terpenoid cyclization reactions in biology. Science 277, 1820–1824 (1997). 9295272 10.1126/science.277.5333.1820

[b35] Cane D. E., Xue Q. & Fitzsimons B. C. Trichodiene synthase. Probing the role of the highly conserved aspartate-rich region by site-directed mutagenesis. Biochemistry 35, 12369–12376 (1996). 8823172 10.1021/bi961344y

[b36] Rynkiewicz M. J., Cane D. E. & Christianson D. W. Structure of trichodiene synthase from Fusarium sporotrichioides provides mechanistic inferences on the terpene cyclization cascade. Proc. Natl. Acad. Sci. USA 98, 13543–13548 (2001). 11698643 10.1073/pnas.231313098 PMC61077

[b37] Aubourg S., Lecharny A. & Bohlmann J. Genomic analysis of the terpenoid synthase (AtTPS) gene family of Arabidopsis thaliana. Mol. Genet. Genomics 267, 730–745 (2002). 12207221 10.1007/s00438-002-0709-y

[b38] Furstner A. & Schlecker A. A gold-catalyzed entry into the sesquisabinene and sesquithujene families of terpenoids and formal total syntheses of cedrene and cedrol. Chem. Eur. J. 14, 9181–9191 (2008). 18785666 10.1002/chem.200801382

[b39] Fujisawa M., Harada H., Kenmoku H., Mizutani S. & Misawa N. Cloning and characterization of a novel gene that encodes (S)-β-bisabolene synthase from ginger, Zingiber officinale. Planta 232, 121–130 (2010). 20229191 10.1007/s00425-010-1137-6

[b40] Faraldos J. A., Gonzalez V., Senske M. & Allemann R. K. Templating effects in aristolochene synthase catalysis: elimination versus cyclisation. Org. Biomol. Chem. 9, 6920–6923 (2011). 21870004 10.1039/c1ob06184d

[b41] Alizadeh B. H., Kuwahara S., Leal W. S. & Men H. C. Synthesis of the racemate of (*Z*)-*exo*-α-bergamotenal, a pheromone component of the white-spotted spined bug, Eysarcoris parvus uhler. Biosci. Biotechnol. Biochem. 66, 1415–1418 (2002). 12162572 10.1271/bbb.66.1415

[b42] Rani A., Ravikumar P., Reddy M. D. & Kush A. Molecular regulation of santalol biosynthesis in Santalum album L. Gene. 527, 642–648 (2013). 23860319 10.1016/j.gene.2013.06.080

[b43] Kolosova N. *et al.* Isolation of high-quality RNA from gymnosperm and angiosperm trees. Biotechniques 36, 821–824 (2004). 15152602 10.2144/04365ST06

[b44] Bradford M. M. A rapid and sensitive method for the quantitation of microgram quantities of protein utilizing the principle of protein-dye binding. Anal. Biochem. 72, 248–254 (1976). 942051 10.1016/0003-2697(76)90527-3

[b45] O’Maille P. E., Chappell J. & Noel J. P. A single-vial analytical and quantitative gas chromatography-mass spectrometry assay for terpene synthases. Anal. Biochem. 335, 210–217 (2004). 15556559 10.1016/j.ab.2004.09.011

[b46] Tamura K., Stecher G., Peterson D., Filipski A. & Kumar S. MEGA6: Molecular Evolutionary Genetics Analysis Version 6.0. Mol. Biol. Evol. 30, 2725–2729 (2013). 24132122 10.1093/molbev/mst197 PMC3840312

