# Supplementary material for: Functional Characterization of Novel Sesquiterpene Synthases from Indian Sandalwood, Santalum album
Source: Sci Rep. 2015 May 15;5:10095. doi: 10.1038/srep10095 (PMC4432371; doi:10.1038/srep10095)
Supplement: Supplementary Information [file srep10095-s1.pdf]

Supplementary Information

Functional characterization and delineation of molecular regulation of sesquiterpene synthases in Indian Sandalwood, *Santalum album*

Prabhakar Lal Srivastava[1], Pankaj P. Daramwar [1], Ramakrishnan Krithika[1], Avinash Pandreka[1,2], S. Shiva Shankar[1] and Hirekodathakallu V. Thulasiram[1,2]*

[1]Chemical Biology Unit, Division of Organic Chemistry, CSIR- National Chemical Laboratory, Dr. Homi Bhabha Road, Pune 411008.

[2]CSIR-Institute of Genomics and Integrative Biology, Mall Road, New Delhi 110007.

[*]To whom correspondence should be addressed: hv.thulasiram@ncl.res.in

Section I: Supporting Methods:

S1.1.Protocol for RNA isolation:

- 1g of plant tissue was ground in liquid nitrogen with 0.3 g of PVPP per gram of tissue

  Critical step: important for minimizing the oxidation of phenolics.

- Finely crushed powder was transferred to a tube and it was washed with acetone to remove phenolic contamination.

  Critical step: This step is very important to remove the phenolics (a yellow colored supernatant was seen due to the presence of phenolics).

- This was centrifuged at 3000 × *g* for 10 min at 4 ºC.
- Pellet was dried and extraction buffer (20 mL/g of tissue) was added to it.
- The mixture was vortexed at room temp for 10 min.
- This was centrifuged at 3000 × *g* for 20 min at 4 ºC.
- The supernatant was transferred to a fresh tube and incubated on ice.
- To this, 10 % CTAB solution (2 mL) was added at room temperature and incubated for 5 min at 60 ºC to remove the residual polysaccharides.

  Critical step: This step is required to remove the polysaccharides to avoid their co-precipitation, which later leads to the degradation of RNA.

- This mixture was extracted with chloroform: isoamyl alcohol (24:1) till a clear interface was observed and the supernatant was retrieved each time.

  Critical step: Extraction should be performed till a clear interface is observed to avoid contamination of the protein.

- The supernatant was transferred to another tube and $1/9^{th}$ volume of 3.3 M sodium acetate and 0.6 volume of ice-cold isopropanol were added.
- This was incubated at -20 ºC for 3 h and then centrifuged at 14000 × *g* for 20 min at 4 ºC and the supernatant was discarded.
- To the pellet, 1 mL of TE buffer (10 mM) and 1 mL of 5 M NaCl were added and the mixture was incubated on ice, with periodic vortexing.
- This mixture was extracted with chloroform: isoamyl alcohol (24:1) till a clear interface was observed and the supernatant was retrieved each time.

Critical step: Extraction should be performed till a clear interface is observed to avoid the contamination of protein.

- To the supernatant, 2.5 M final concentration of LiCl was added and was incubated overnight at -20 ºC.
- The RNA was pelleted down by centrifugation at 14,000 × *g* for 30 min at 4 ºC.
- The resultant pellet was washed with 70 % ethanol by centrifugation at 14,000 × *g* for 10 min at 4 ºC.
- The pellet was dried at room temperature and re-suspended in 50 µL DEPC treated water.
- The integrity of total RNA was evaluated from 1.5 % Agarose gel electrophoresis (Figure S1).

S1.2. Isolation and cloning of sesquiterpene synthases in expression vector:

Coding sequences of prenyl transferase (SaFDS) and sesquiterpene synthases (SaSQS1, SaSQS2, SaSS and SaBS) were amplified from cDNA using full-length ORF primers having RE site at both the ends (Table S2). PCR was performed using Proof reading Taq DNA polymerase (Invitrogen) and PCR products were purified from agarose gel and digested with respective restriction enzymes (NEB) incorporated at their ends and were ligated with respective expression vectors (Table S3).

S1.3. Bacterial expression and purification of active protein of SaFDS, SaSQS1, SaSQS2, SaBS and SaSS:

For the expression of recombinant active protein, recombinant plasmids, such as pRSETB harbouring SaFDS was introduced into BL21 DE3 competent cells, whereas, pET32b harbouring coding sequence of SaSS and pET28a harbouring coding sequence of SaSQS1, SaSQS2 and SaBS were introduced in Rosetta 2 DE3 competent cells. A single colony was inoculated in 5 mL LB media containing 100 µg/mL ampicillin for pRSETB construct, 34 µg/mL chloramphenicol and 100 µg/mL ampicillin (for pET32b construct) or 50 µg/mL of kanamycin for pET28a construct and were incubated at 37 ºC and 200 rpm overnight. Aliquots (1 mL) of these overnight grown cultures were transferred to 100 mL of Terrific broth (LB broth for pRSETB construct) and further incubated at 37 ºC and 200 rpm till an absorbance at 600nm reached 0.8. Cultures were induced with 0.5 mM IPTG and were incubated at 16 ºC overnight (pRSETB construct was incubated at 30 ºC for 6 h). The cultures were then harvested by

centrifugation at 4500 × *g* at 4 °C, flash frozen and stored at -80 °C till further use. Cell pellets were suspended in lysis buffer (50 mM NaH_2PO_4, 300 mM NaCl, 10 % glycerol, pH 7.4, containing lysozyme 1 mg/mL) and were incubated on ice for 30 min, after which they were sonicated using probe sonicator for 5 cycles (pulse on 30 sec, pulse off 30 sec, amplitude 70 %). Lysed suspensions were centrifuged at 10,000 × *g* for 10 min. Clear lysates were mixed with Ni-NTA slurry and incubated at 4 °C for 1 h on rocker and then loaded on a protein purification column of 1 cm diameter. After collecting the unbound fraction, column was washed with wash buffer (50 mM NaH_2PO_4, 300 mM NaCl, 30 mM imidazole, 10 % glycerol, pH 7.4), and finally it was eluted into eight fractions of 0.5 mL with elution buffer (50 mM NaH_2PO_4, 300 mM NaCl, 250 mM imidazole, 10 % glycerol, pH 7.4). Purified protein fractions were pooled together and desalted in HEPES buffer (25 mM HEPES, 100 mM KCl, 10 % glycerol, pH 7.4) on HiPrep™ 26/10 desalting column using ACTA (GE Healthcare). Protein concentration was determined using Bradford method [1] and all the fractions were analyzed on 10 % SDS-PAGE (Figs. S9, S12, S15, S18 and S21).

S1.4 Preparative scale enzyme assay

For the characterization of SaSQS1 product, preparative scale enzyme assay was set up using 80 mg of purified and desalted protein with 60 mg of (*E*,*E*)-FPP in HEPES buffer, pH 7.4 in a final reaction volume of 100 mL. Reaction mixture was overlaid by n-hexane and incubated at 30 °C for 24 h. After incubation, reaction mixture was extracted with n-hexane (2 × 100 mL) and was analyzed by GC/GC-MS. The crude extract was purified over silica layers to obtain the pure product that was analyzed by ^{1}H, ^{13}C NMR and GC-QToF. On analysis, the product was characterized as sesquisabinene (**7**) and the spectral data obtained was found to match with the literature reports [2].

S1.5: Cloning of SaFDS and SaSQS1/SaSQS2 in pETDuet-1 vector for *in-vivo* production of Sesquisabinene

SaFDS isolated from transcriptome screening was sub-cloned in pETDuet-1 vector frame using BamHI site in multiple cloning site 1. Similarly SaTPS1 was sub-cloned in multiple cloning site 2 of pETDuet-1:SaFDS using NdeI and KpnI, whereas SaTPS2 was sub-cloned in multiple cloning site 2 of pETDuet-1:SaFDS using KpnI restriction site.

S1.6 Spectral data for characterization of sesquiterpenes

Spectral data for **α-Santalene** and **(β + *epi*-β)-santalene** isolated from 'Terpene fraction' of sandalwood oil has been reported earlier by our group [3].

Sesquisabinene (7) [2]: $[\alpha]^{25}_{D}$ = -17.4 (*c*=0.91 in $CHCl_3$); ^{1}H NMR (400 MHz, $CDCl_3$): δ 5.12-5.09 (m, 1H), 4.82 (s, 1H), 4.63 (s, 1H), 2.16 (dd, *J* = 15.65, 9.05 Hz, 1H), 2.08-2.00 (m, 3H), 1.77-1.66 (m, 2 H), 1.70 (s, 3H), 1.62 (br s, 4H), 1.47-1.38 (m, 1H), 1.25-1.17 (m, 2H), 0.95 (d, *J* = 6.36 Hz, 3H), 0.75 (m, 1H), 0.67 (m, 1H); ^{13}C NMR (100 MHz, $CDCl_3$): δ 154.5 (=C), 131.2 (=C), 124.9 (=CH), 101.6 (=CH_2), 38.0 (-CH), 36.8 (-C), 35.2 (-CH_2), 29.7 (-CH), 28.9 (-CH_2), 26.2 (-CH_2), 26.1 (-C), 25.7 (-CH_3), 18.3 (-CH_2), 17.7 (-CH_3), 17.5 (-CH_3); GC-EI-MS (70 eV): *m*/*z*: 204.2 [M^+], 161.2, 133.1, 120.1, 105.1, 93.0, 79.1, 69.0 (100 %), 55.0; GC-QToF-EI-MS: *m*/*z*: calcd for $C_{15}H_{24}$ [M^+]: 204.1878, found: 204.1910.

β-Bisabolene (9): $[\alpha]^{25}_{D}$ = -40.9 (*c*=0.95 in $CHCl_3$) (reported $[\alpha]^{25}_{D}$ = -51.2, *c*=0.328 in EtOH) [4]; ^{1}H NMR (400 MHz, $CDCl_3$)[5]: δ 5.41 (m, 1H), 5.13 (tquin, *J* =1.25, 6.77 Hz, 1H), 4.75 (m, 2H), 1.88-2.15 (m, 8H), 1.79-1.85 (m, 1H), 1.69 (d, *J* = 0.76 Hz, 3H), 1.65 (s, 3H), 1.61 (s, 3H), 1.41-1.52 (m, 2H); ^{13}C NMR (100 MHz, $CDCl_3$) δ 154.3 (=C), 133.8 (=C), 131.5 (=C), 124.3 (=CH), 120.8 (=CH), 107.1 (=CH_2), 39.8 (-CH), 34.9 (-CH_2), 31.4 (-CH_2), 30.8 (-CH_2), 28.3 (-CH_2), 26.8 (-CH_2), 25.7 (-CH_3), 23.5 (CH_3), 17.7 (CH_3); GC-EI-MS (70 eV): *m*/*z*: 204.2 [M^+], 189.2, 161.1, 147.1, 134.1, 119.1, 107.1, 93.1, 69.0 (100 %), 53.1; GC-QToF-EI-MS : *m*/*z*: calcd for $C_{15}H_{24}$ [M^+]: 204.1878, found: 204.1902.

(*E*)-β-Farnesene (6): ^{1}H NMR (500 MHz, $CDCl_3$): δ 6.38 (dd, *J* = 10.83, 17.54 Hz, 1H), 5.25 (d, *J* = 17.60 Hz, 1H), 5.16 (tq, *J* = 1.22, 6.71 Hz, 1H), 5.10 (tquin, *J* = 1.52, 6.87 Hz, 1H), 5.06 (dq, *J* = 0.61, 10.68 Hz, 1H), 5.02 (bs, 1H), 5.00 (bs, 1H), 2.16-2.25 (m, 4H), 2.07 (m, 2H), 1.99 (m, 2H), 1.69 (q, *J* = 1.22 Hz, 3H), 1.61 (bs, 6H); ^{13}C NMR (125 MHz, $CDCl_3$) δ 146.1 (=C), 139.0 (=CH), 135.4 (=C), 131.3 (=C), 124.3 (=CH), 124.0 (=CH), 115.7 (=CH_2), 113.1 (=CH_2), 39.7 (-CH_2), 31.4 (-CH_2), 26.7 (-CH_2), 26.6 (-CH_2), 25.7 (-CH_3), 17.7 (-CH_3), 16.0 (-CH_3)[6]; GC-EI-MS (70 eV): *m*/*z*: 204.2 [M^+], 189.1, 161.1, 148.1, 133.0, 120.1, 107.1, 93.0, 79.0, 69.0 (100 %), 55.1; GC- QToF-EI-MS: *m*/*z*: calcd for $C_{15}H_{24}$ [M^+]: 204.1878, found: 204.1871.

***exo*-α-Bergamotene (4)**: $[\alpha]^{25}_{D}$ = -42.4 (c=2 in $CHCl_3$); ^1H NMR (500 MHz, $CDCl_3$) [7]: δ 5.20-5.22 (m, 1H), 5.17 (tquin, J = 1.22, 7.02 Hz, 1H), 2.30-2.34 (m, 1H), 2.22-2.27 (m, 1H), 2.11-2.17 (m, 2H), 1.94-2.01 (m, 3H), 1.70 (br s, 3H), 1.66 (q, J = 1.8 Hz, 3H), 1.63 (br s, 3H), 1.59-1.64 (m, 2H), 1.17 (d, J = 8.54 Hz, 1H), 0.83 (s, 3H); ^{13}C NMR (125 MHz, $CDCl_3$)[8]: δ 144.5 (=C), 130.9 (=C), 125.3 (=CH), 116.5 (=CH), 45.4 (-CH), 41.1 (-C-), 38.9 (-CH), 38.6 ($-CH_2$), 31.6 ($-CH_2$), 31.2 ($-CH_2$), 25.7 ($-CH_3$), 23.8 ($-CH_2$), 23.0 ($-CH_3$), 17.6 ($-CH_3$), 17.4 ($-CH_3$); GC-EI-MS (70 eV): m/z: 204.2 [M^+], 189.1, 161.1, 147.1, 133.1, 119.0 (100 %), 107.1, 93.0, 79.0, 69.1, 55.1; GC- QToF-EI-MS: m/z: calcd for $C_{15}H_{24}$ [M^+]: 204.1878, found: 204.1877.

***exo*-β-Bergamotene (5)**: ^1H NMR (500 MHz, $CDCl_3$)[7]: δ 5.15-5.18 (m, 1H), 4.63 (m, 1H), 4.56 (m, 1H), 2.52-2.54 (m, 2H), 2.26-2.32 (m, 2H), 2.04-2.08 (m, 1H), 1.93-1.98 (m, 2H), 1.81-1.85 (m, 2H), 1.70 (s, 3H), 1.62 (s, 3H), 1.60-1.64 (m, 2H), 1.42 (d, J = 9.77 Hz, 1H), 0.71 (s, 3H); ^{13}C NMR (125 MHz, $CDCl_3$) δ 152.2 (=C), 131.1 (=C), 125.1 (=CH), 106.0 ($=CH_2$), 50.2 (-CH), 43.8 (-C-), 38.6 (-CH), 38.2 ($-CH_2$), 31.9 ($-CH_2$), 29.4 ($-CH_2$), 27.1 ($-CH_3$), 25.7 ($-CH_2$), 23.5 ($-CH_3$), 18.6 ($-CH_3$), 14.1 ($-CH_3$); GC-EI-MS (70 eV): m/z: 204.1 [M^+], 189.1, 161.1, 133.1, 120.1, 107.1, 93.0, 79.0, 69.0 (100 %), 55.1. GC- QToF-EI-MS: m/z: calcd for $C_{15}H_{24}$ [M^+]: 204.1878, found: 204.1872.

References:

1. Bradford, M.M. A rapid and sensitive method for the quantitation of microgram quantities of protein utilizing the principle of protein-dye binding. *Anal. Biochem.* **72**, 248-254 (1976).
2. Furstner, A. & Schlecker, A. A gold-catalyzed entry into the sesquisabinene and sesquithujene families of terpenoids and formal total syntheses of cedrene and cedrol. *Chem. Eur. J.* **14**, 9181-9191 (2008).
3. Daramwar, P.P., Srivastava, P.L., Priyadarshini, B. & Thulasiram, H.V. Preparative separation of α- and β-santalenes and (Z)-α- and (Z)-β-santalols using silver nitrate-impregnated silica gel medium pressure liquid chromatography and analysis of sandalwood oil. *Analyst* **137**, 4564-4570 (2012).
4. Argenti, L., Bellina, F., Carpita, A. & Rossi, R. Asymmetric Synthesis of Highly Enantiomerically Enriched (S)(-)-β-Bisabolene. *Syn. Comm.* **25**, 2909-2921 (1995).
5. Argenti, L., Bellina, F., Carpita, A., Dell'Amico, N. & Rossi, R. Termite Trail Attractants: New Syntheses of Racemic (E)-α-, (Z)-α- and β-Bisabolenes. *Syn. Comm.* **24**, 3167-3188 (1994).

6. Arkoudis, E. & Stratakis, M. Synthesis of Cordiaquinones B, C, J, and K on the Basis of a Bioinspired Approach and the Revision of the Relative Stereochemistry of Cordiaquinone C. *J. Org. Chem.* **73**, 4484-4490 (2008).

7. Alizadeh, B.H., Kuwahara, S., Leal, W.S. & Men, H.C. Synthesis of the racemate of (*Z*)-*exo*-α-bergamotenal, a pheromone component of the white-spotted spined bug, *Eysarcoris parvus* uhler. *Biosci. Biotechnol. Biochem.* **66**, 1415-1418 (2002).

8. Snider, B.B. & Beal, R.B. Total synthesis of sesquiterpenes via intramolecular ketene cycloadditions. Isocomene. α-*cis*- and α-*trans*-bergamotenes. An approach to seychellene. *J. Org. Chem.* **53**, 4508-4515 (1988).

9. Fujisawa, M., Harada, H., Kenmoku, H., Mizutani, S. & Misawa, N. Cloning and characterization of a novel gene that encodes (S)-β-bisabolene synthase from ginger, *Zingiber officinale*. *Planta* **232**, 121-130 (2010).

Table S1. Primer sequence for RACE and isolation of full length ORF of SaFDS and terpene synthases

Primer name	5'-3' Primer sequence
3' RACE universal primer	GCTGTCAACGATACGCTACGTAACG
3' RACE nested universal primer	CGCTACGTAACGGCATGACAGTG
5' RACE SaFDS	TGCGATGGTGAATAGGCATTGAAT
3' RACE SaFDS	TTCTTGTTCTCGATGATATTATGGA
3' RACE SaSQS1	GGTTCCAGAACAAGGAAACTGGCAAAT
3' RACE SaSQS2	CACATATGAAGAAGAAGAAGACGTGAACC
5' RACE SaSS	CTCCACATATAGCTCTGCAGTAAATT
3' RACE SaSS	AGCGAATATGAACCCAACACTACTCA
3' RACE SaBS	GTTCCAGAACAAGGAAACTGGCAAAT
SaFDS_F	GAG**GGATCC**ATGGGCGATCGGAAAACCAAAT
SaFDS_R	GAG**CTGCAG**CTACTTCTGCCGCTTGTATATCT
SaSS_F	GAG**GATATC**ATGGATTCTTCCACCGCCACC
SaSS_R	GAG**GAGCTC**CTACTCCTCGCCGAGAGGAATAG
SaSQS1_F	GAG**GATATG**ATGGATTTGTGTCAGATCCCGCCCACCTCT
SaSQS1_R	GAG**GGATCC**TTACTCCTCATCTAGCGTAATTGGGTGAAT
SaSQS2_F	GAG**GGATCC**ATGGCCTCTGTGATTGTTGAACCCATTCGT
SaSQS2_R	GAG**CTCGAG**CTACTCTTCATTGAGTGGAATTGGATGGATC
SaBS_F	GAG**GGATCC**ATGGATGCCTTTGCCACTTCTCCGACCT
SaBS_R	GAG**GCGGCCGC**TCAATCCTCCTCGTTCAGTGGAATAGGG

Table S2. Restriction enzymes used for cloning of terpene synthases in expression vectors

Sr. No.	Enzyme	Restriction site used for cloning	Expression vector	Expression cells
1.	SaFDS	BamHI at 5' and PstI at 3'	pRSETB	BL21 (DE3)
2.	SaSQS1	Nde1 at 5' and BamH1 at 3'	pET28a	Rosetta 2 DE3
3.	SaSQS2	BamH1 at 5' and Xho1 at 3'	pET28a	
4.	SaBS	BamHI at 5' and NotI at 3'	pET28a	
5.	SaSS	EcoRV at 5' and SacI at 3'	pET32b	

Table S3. Primer sequence for semi-quantitative real time analysis

Primer name	5'-3' Primer sequence
Sa18S_F	TGACGGAGAATTAGGGTTCG
Sa18S_R	GTGCCAGCGGAGTCCTATAA
SaFDS_F	ATGGGCGATCGGAAAACCAAAT
SaFDS_R	CTACTTCTGCCGCTTGTATATCT
SaSS_F	ATGGATTCTTCCACCGCCACC
SaSS_R	CTACTCCTCGCCGAGAGGAATAG
SaSQS1_F	ATGGATTTGTGTCAGATCCCGCCCACCTCT
SaSQS1_R	TTACTCCTCATCTAGCGTAATTGGGTGAAT
SaSQS2_F	ATGGCCTCTGTGATTGTTGAACCCATTCGT
SaSQS2_R	CTACTCTTCATTGAGTGGAATTGGATGGATC
SaBS_F	ATGGATGCCTTTGCCACTTCTCCGACCT
SaBS_R	TCAATCCTCCTCGTTCAGTGGAATAGGG

Table S4: GC/GC-MS program

**Program 1**<br>HP-5 column	A temperature gradient from 70 °C to 170 °C at 5 °C/min, followed by a second temperature gradient of 15 °C/min from 170 °C to 180 °C and a final hold at 180 ºC for 5 min.
**Program 2**<br>Astec CHIRAL DEX™ B-DA Capillary Column	A temperature gradient from 70 °C to 100 °C at 5 °C/min, followed by a second temperature gradient of 1 °C/min from 100 °C to 220 °C and a final hold at 220 ºC for 5 min.
**Program 3** for analysis of assay sample with GPP and NPP<br>HP-5 column	A temperature gradient from 50 °C to 100 °C at 2 °C/min, followed by a second temperature gradient of 10 °C/min from 100 °C to 180 °C and a final hold at 180 ºC for 2 min.
**Program 4** for characterization of bisabolene<br>HP-Chiral column	A temperature gradient from 50 °C to 95 °C at 1 °C/min and hold for 15 minutes, followed by a second temperature gradient of 10 °C/min from 95 °C to 180 °C and a final hold at 180 ºC for 2 min.

Section II: Molecular images for transcriptome screening, cloning and expression

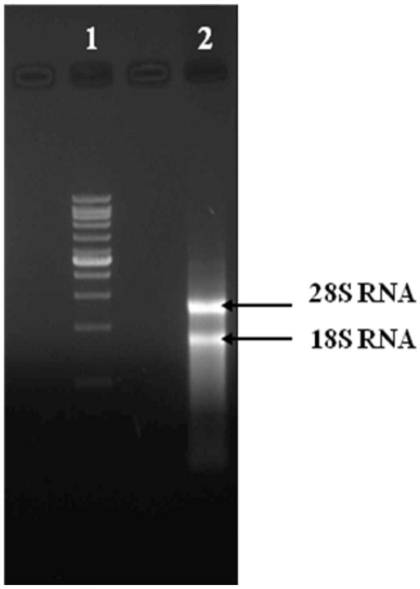

Figure S1: RNA gel image, **Lane 1:** DNA ladder, **Lane 2:** Total RNA $A_{260/280}$ ratio = 2.14, $A_{260/230}$ ratio = 2.35.

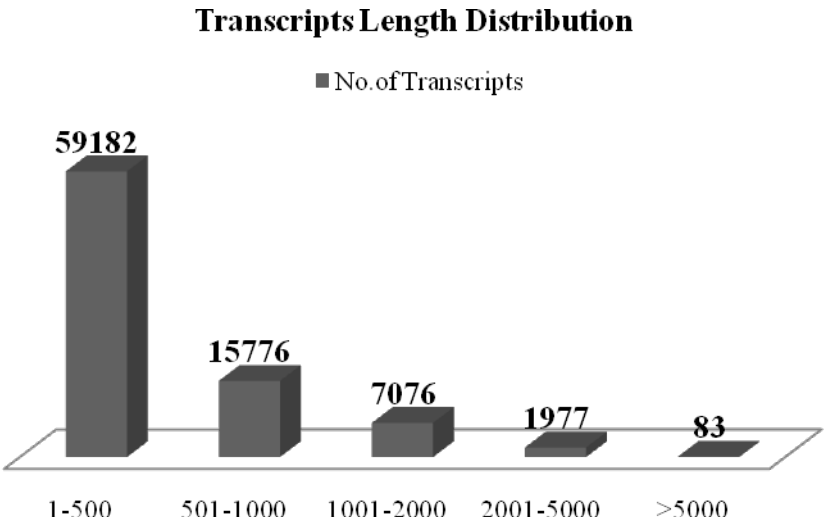

Figure S2: Transcript length distribution graph.

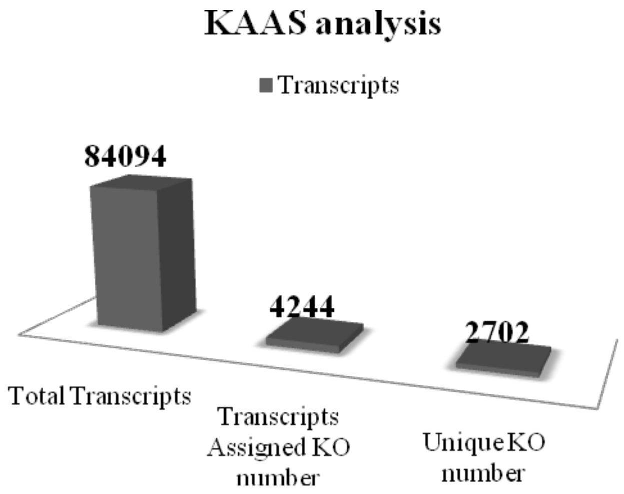

Figure S3: KAAS analysis of unigenes.

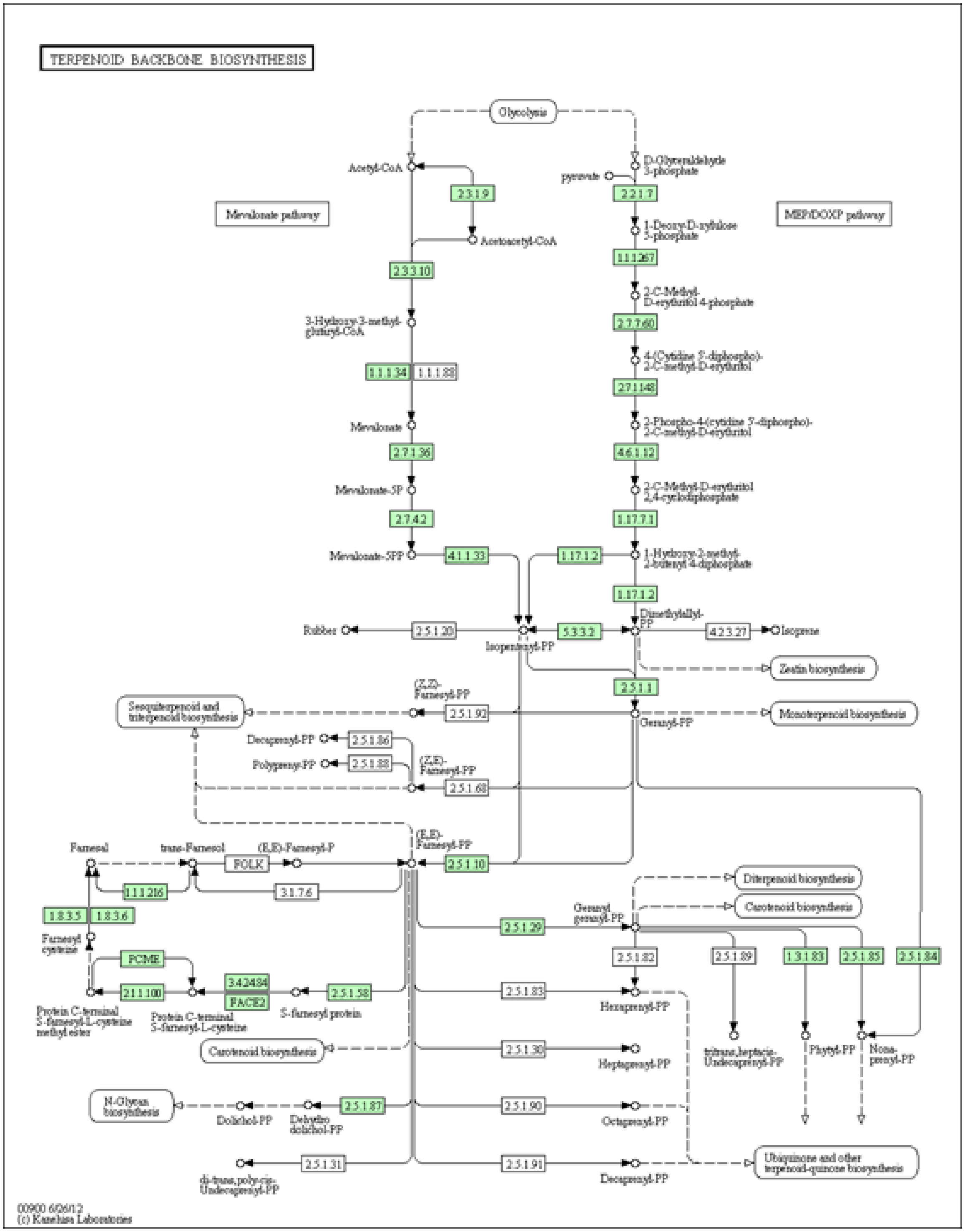

Figure S4: KEGG pathways map for terpenoid biosynthesis, green colour highlighted ID represents the presence of these pathway enzymes in our unigenes.

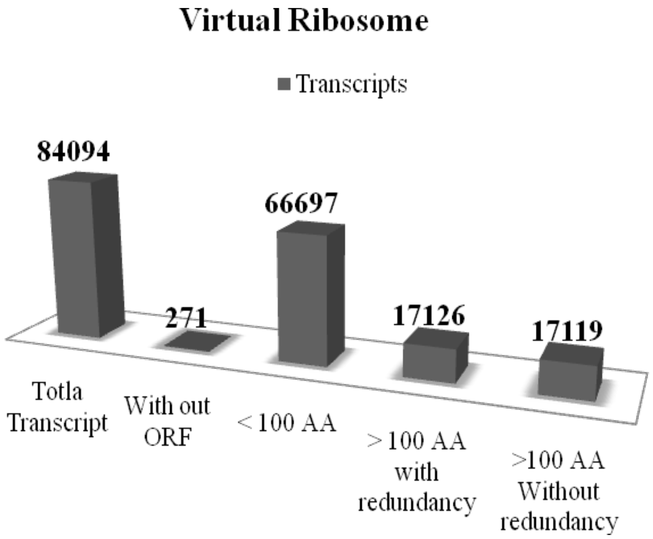

Figure S5: ORF prediction using Virtual Ribosome-V-1.1.

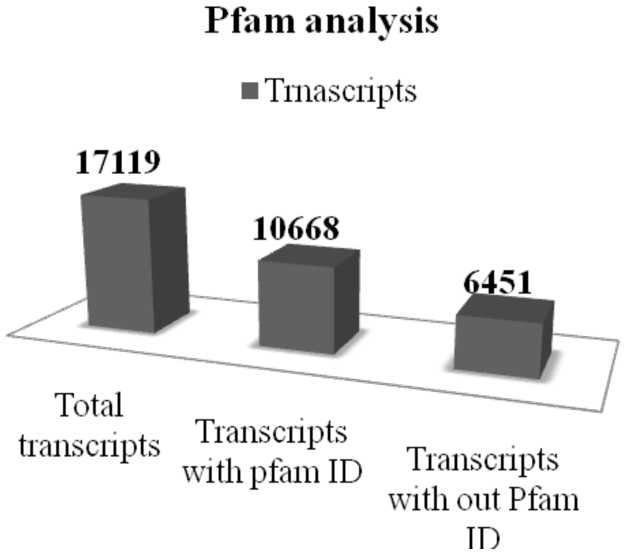

Figure S6: Pfam analysis of transcript having ORF ≥100 amino acid.

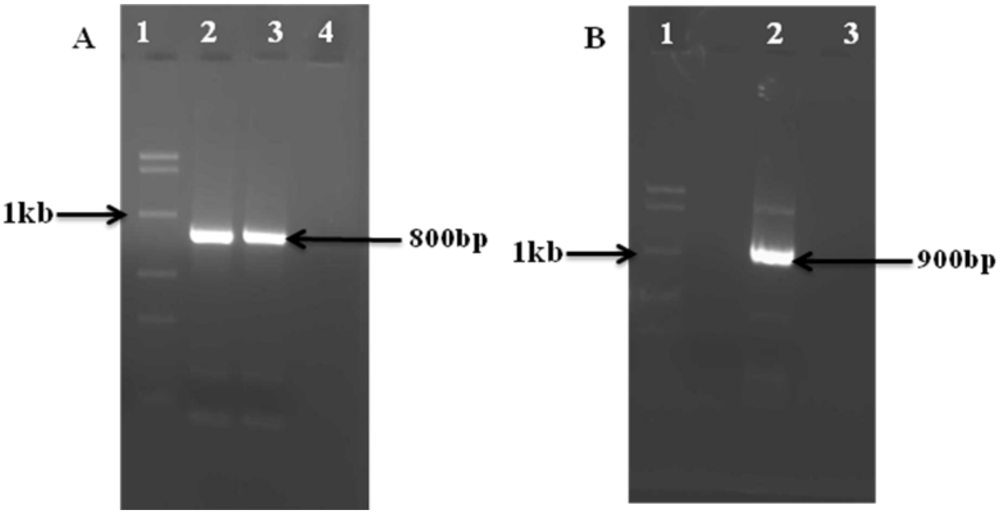

Figure S7: A) SaFDS 3' RACE product, **Lane 1:** 1kb DNA ladder, **Lane 2 and 3:** SaFDS 3' RACE amplicon, **Lane 4:** Negative control, **B)** SaFDS 5' RACE amplicon, **Lane 1:** 1kb DNA ladder, **Lane 2:** SaFPP 5' RACE product, **Lane 3:** Negative control.

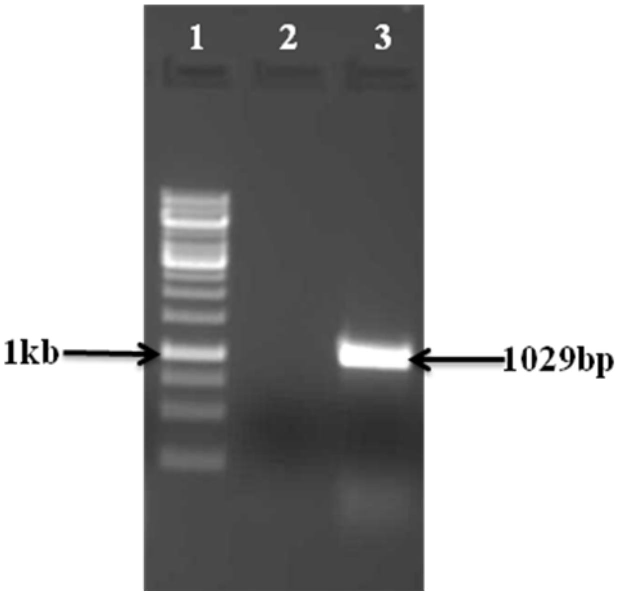

Figure S8: Amplification of SaFDS ORF, **Lane 1:** 1kb DNA ladder, **Lane 2:** Negative control, **Lane 3:** 1029 bp amplicon of SaFDS ORF.

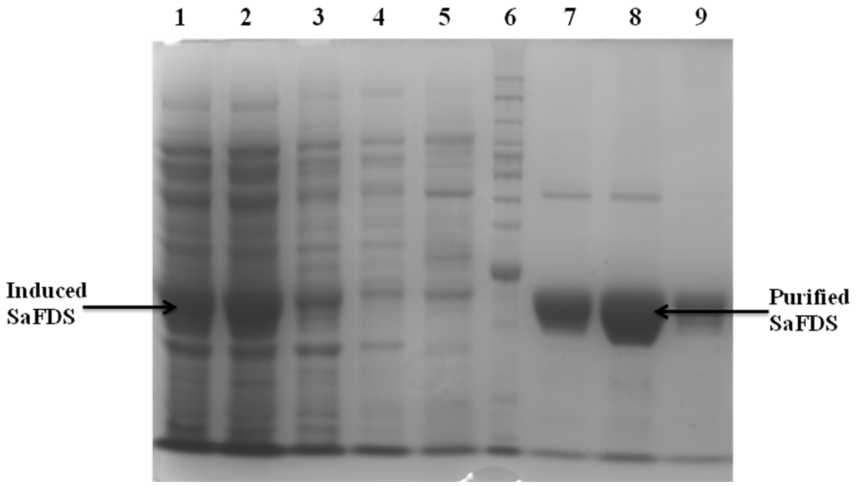

Figure S9: SDS-PAGE for SaFDS protein purification, **Lane 1:** Crude lysate pellet fraction, **Lane 2:** Crude lysate supernatant, **Lane 3:** Unbound fraction, **Lane 4-5:** Wash fraction, **Lane 6:** Protein marker, **Lanes 7-9:** Elution fractions.

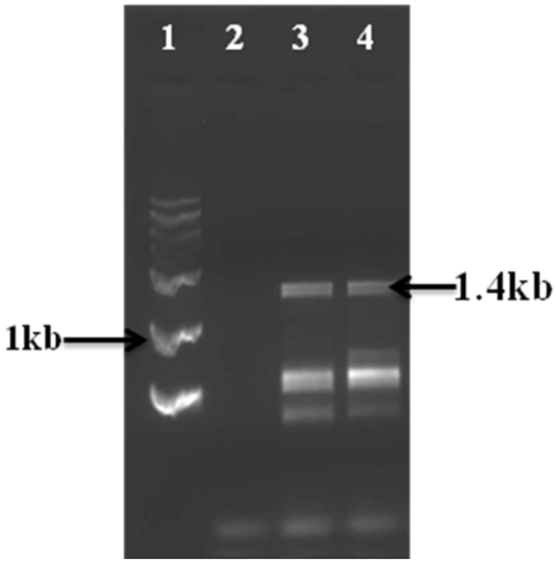

Figure S10: 3' RACE amplification of SaSQS1, **Lane 1:** DNA Ladder, **Lane 2:** Negative control, **Lane 3-4:** 3' RACE amplicon of SaSQS1.

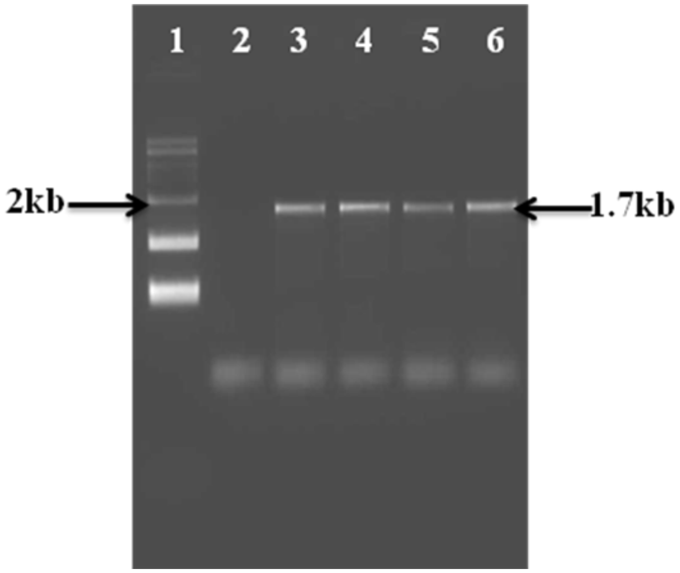

Figure S11: Amplification of SaSQS1 ORF, **Lane 1:** DNA ladder, **Lane 2:** Negative control, **Lane 3-6:** 1701 bp amplicon of SaSQS1 ORF.

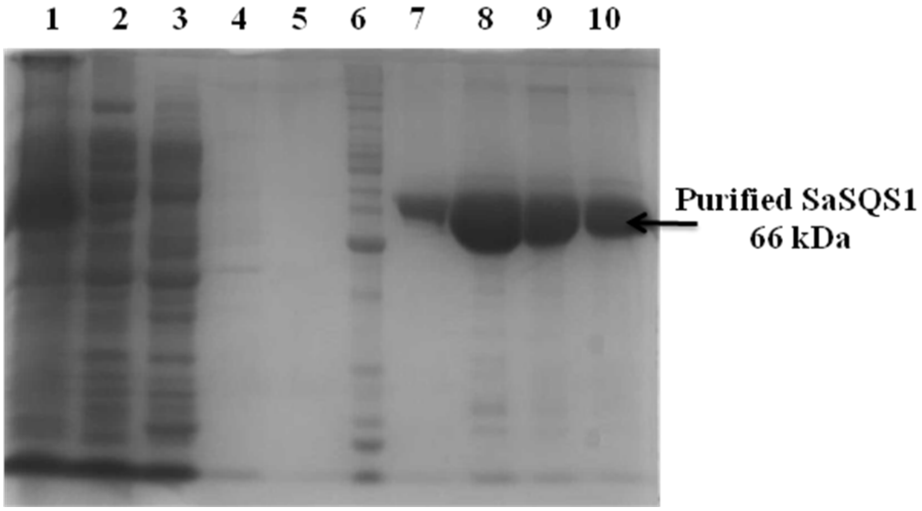

Figure S12: SDS-PAGE of SaSQS1 protein purification, **Lane 1:** Crude lysate, **Lane 2:** Unbound fraction, **Lane 3:** Wash 1, **Lane 4:** Wash 2**: Lane 5:** Wash 3, **Lane 6:** Protein marker, **Lane 7-9:** Elution fraction 1-4.

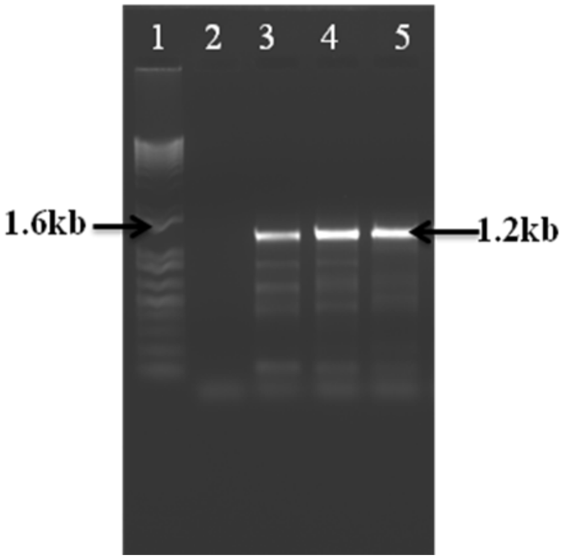

Figure S13: 3' RACE amplification of SaSQS2, **Lane 1:** DNA Ladder, **Lane 2:** Negative control, **Lane 3-5:** 3' RACE amplicon of SaSQS2.

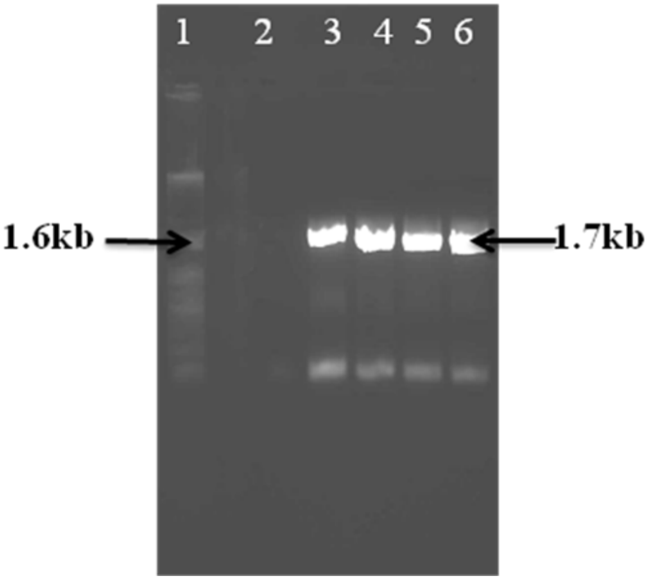

Figure S14: Amplification of ORF of SaSQS2 (Re-PCR of first round PCR product of SaSQS2 with ORF primer), **Lane 1:** DNA ladder, **Lane 2:** Negative control, **Lane 3-6:** 1701 bp amplicon of SaSQS2 ORF.

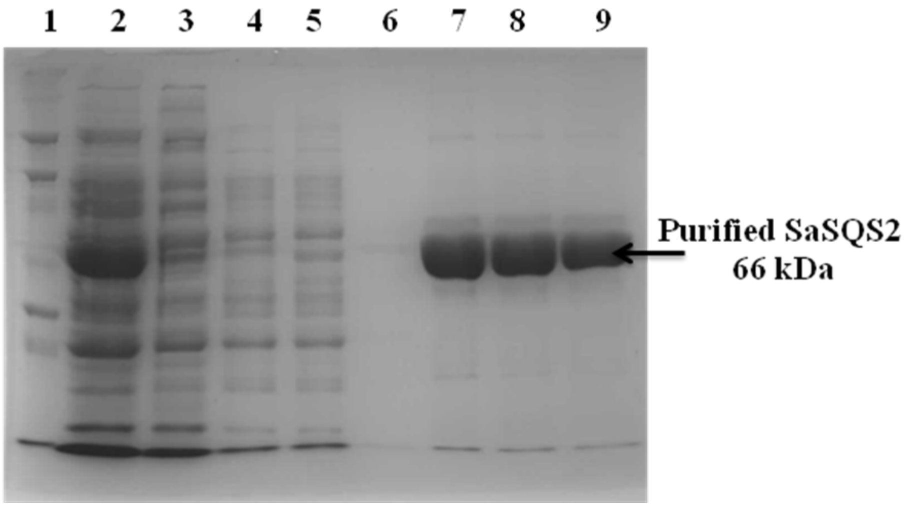

Figure S15: SDS-PAGE of SaSQS2 protein purification, **Lane 1:** Protein marker, **Lane2:** Crude lysate, **Lane 3:** Unbound fraction, **Lane 4:** Wash 1, **Lane 5:** Wash 2, **Lane 6:** Wash 3, **Lane7-9:** Elution fraction 1-3.

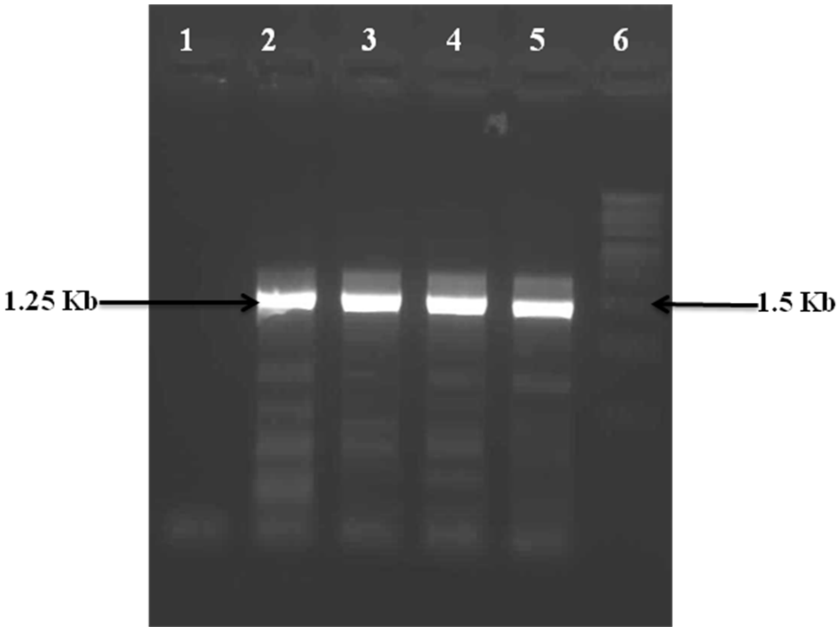

Figure S16: SaBS 3′ RACE amplification, **Lane 1:** Negative control, **Lanes 2-5:** 3′ RACE amplicon of SaBS, **Lane 6:** 1 Kb DNA ladder.

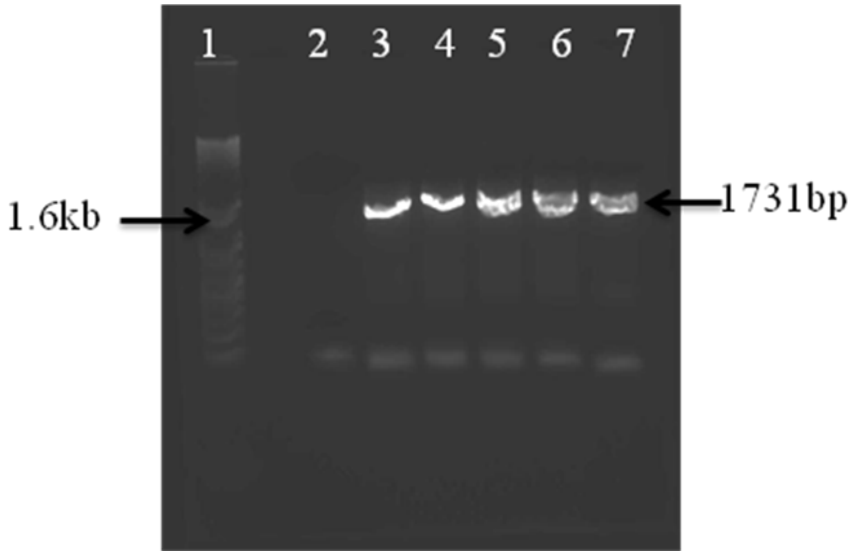

Figure S17: SaBS ORF amplification, **Lane 1:** DNA ladder, **Lane 2:** Negative control, **Lanes 3-6:** 1731 bp amplicon of SaBS ORF.

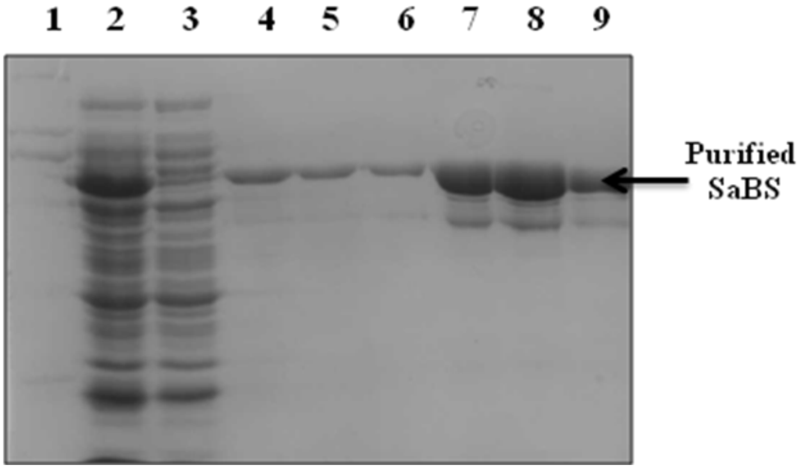

Figure S18: SDS-PAGE of SaBS protein purification, **Lane 1:** Protein marker, **Lane 2:** Crude lysate, **Lane 3:** Unbound fraction, **Lane 4:** Wash 1, **Lane 5:** Wash 2, **Lane 6:** Wash 3, **Lane 7-9:** Elution fractions 1-3.

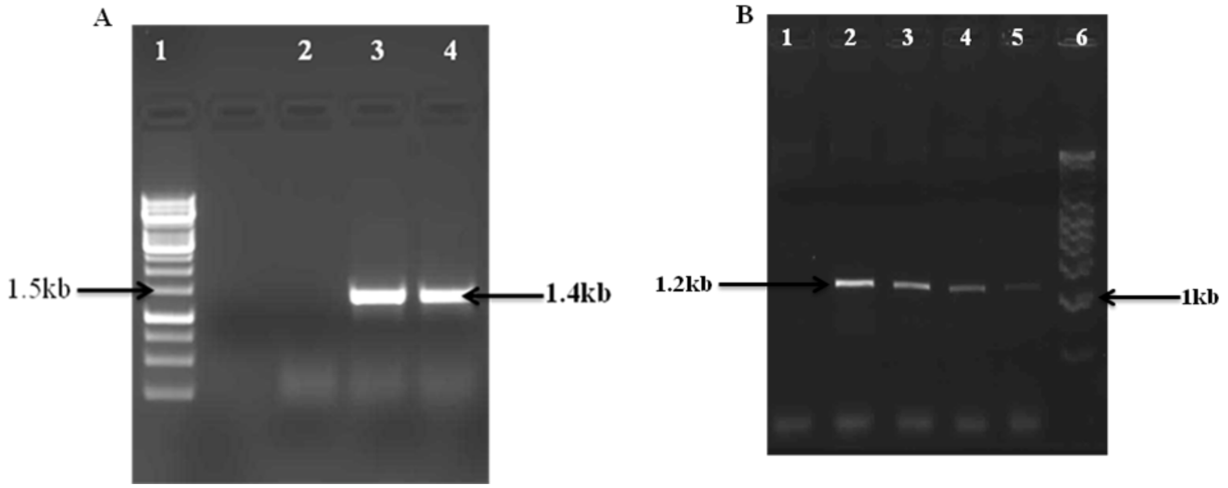

Figure S19 A): SaSS 3' RACE gel image, **Lane 1:** 1 Kb DNA ladder, **Lane 2:** Negative control, **Lanes 3-4:** 3' RACE amplicon of SaSS, **B)** SaSS 5' RACE gel image, **Lane 1:** Negative control, **Lanes 2-5:** 5' RACE amplicon of SaSS **Lane 6:** 1 Kb DNA ladder.

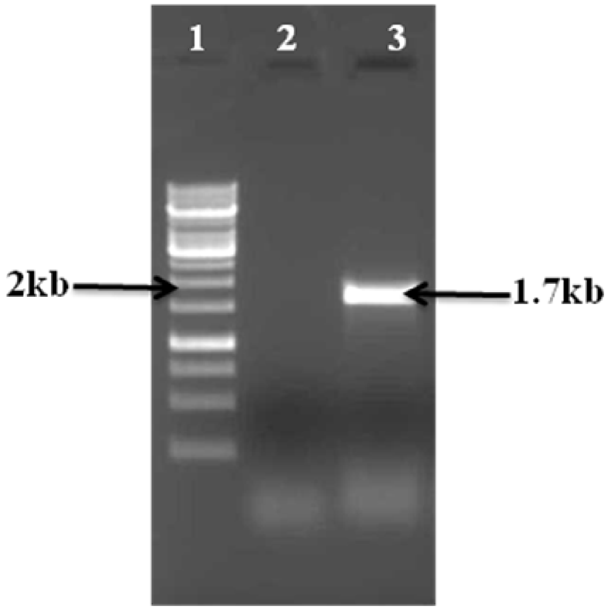

Figure S20: SaSS ORF amplification, **Lane 1:** 1 Kb DNA ladder, **Lane 2:** Negative control, **Lane 3:** 1710 bp amplicon of SaSS ORF.

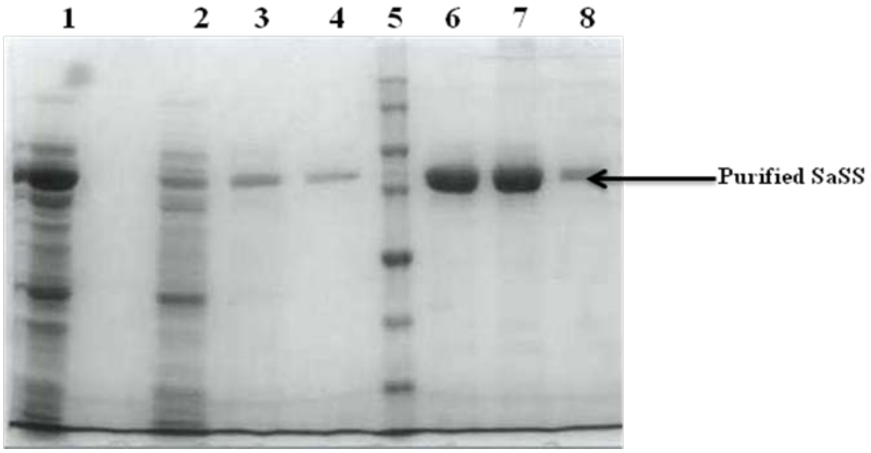

Figure S21: SDS-PAGE gel image of SaSS protein purification, **Lane 1:** Crude lysate of induced SaSS, **Lane 2:** Unbound proteins, **Lanes 3-4:** Wash fractions, **Lane 5:** Protein marker, **Lanes 6 -8:** Elution fraction 1-3.

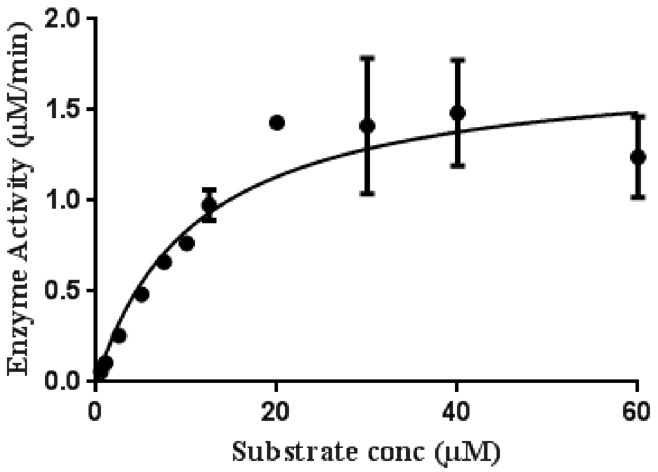

Figure S22: Michaelis Menten plot for SaSQS1 kinetics with varying substrate concentration.

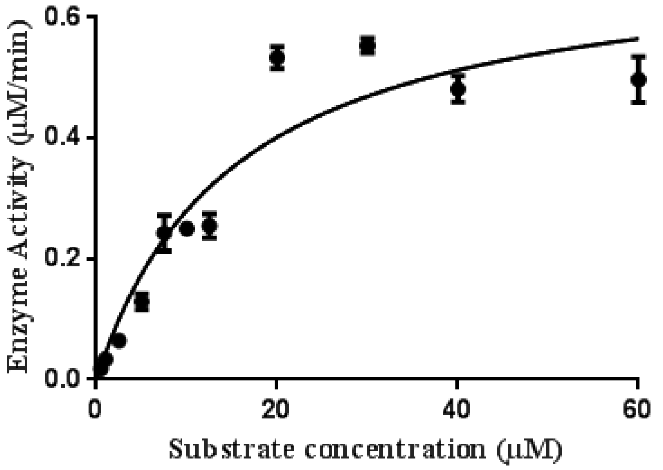

Figure S23: Michaelis Menten plot for SaSQS2 kinetics with varying substrate concentration.

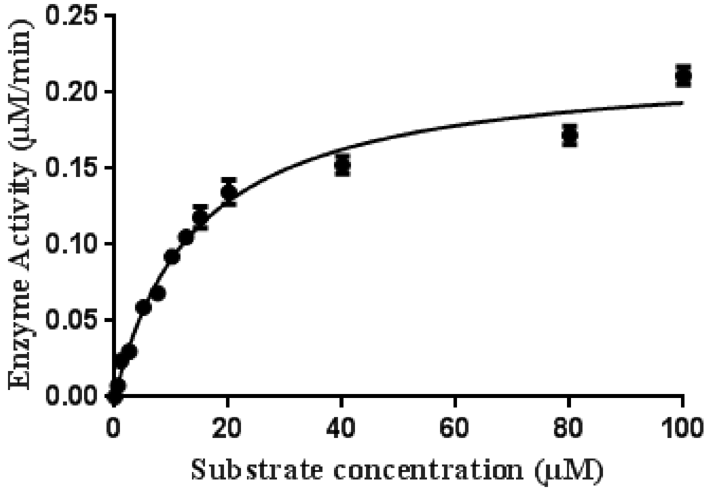

Figure S24: Michaelis Menten plot for SaBS kinetics with varying substrate concentration.

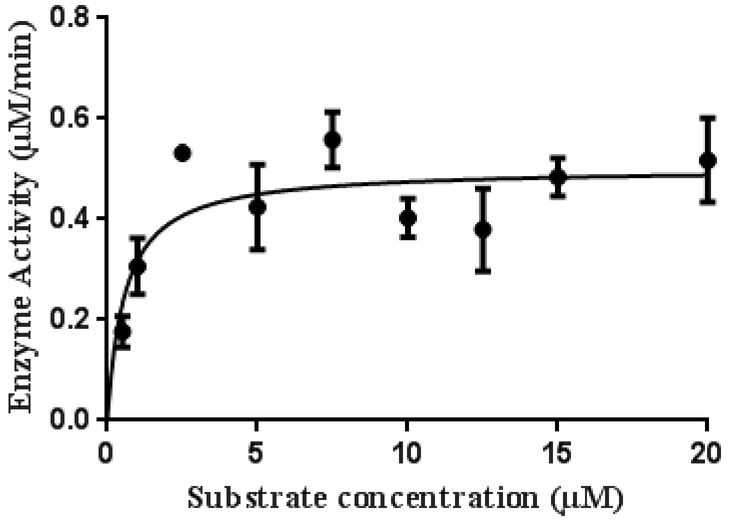

Figure S25: Michaelis Menten plot for SaSS kinetics with varying substrate concentration.

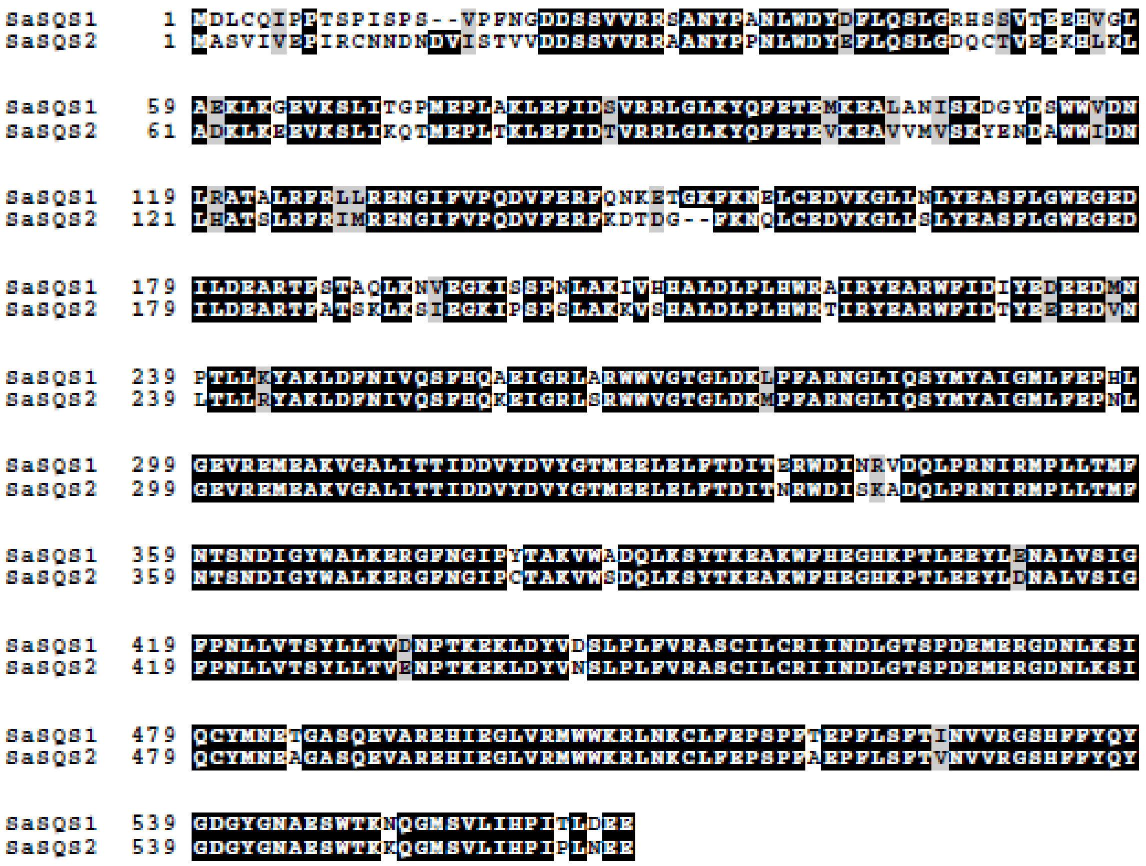

Figure S26: Protein sequence alignment of SaSQS 1 and SaSQS 2.

Section III: GC analysis for characterization of metabolites:

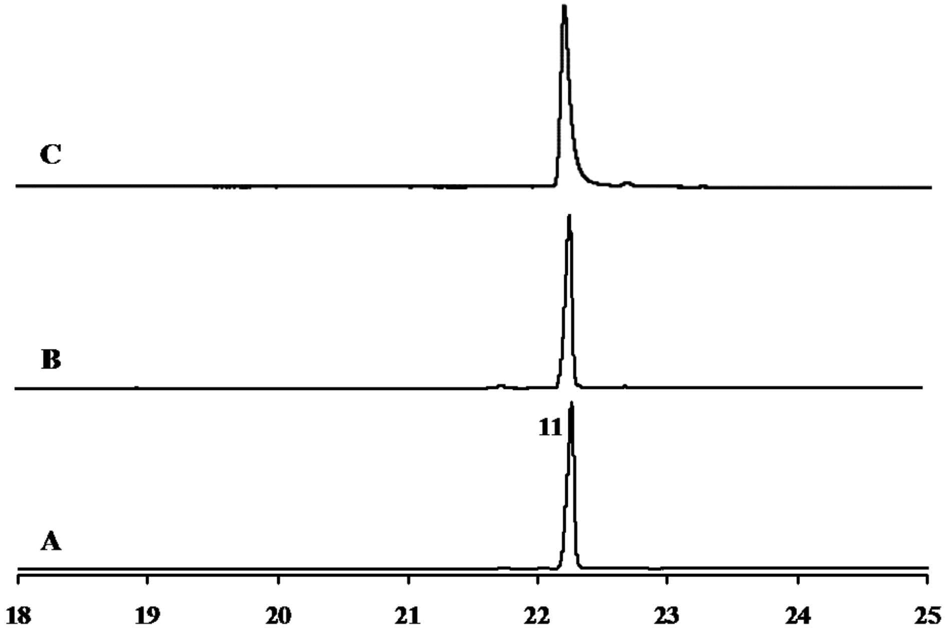

Figure S27. GC traces of SaFDS enzymatic assay product with, (**A**) DMAPP and IPP (1:2 molar ratio), (**B**) GPP and IPP (1:1 molar ratio), (**C**) (*E,E*)-farnesol standard (**11**); GC analysis was performed with HP-5 capillary column (30m × 0.32mm × 0.25µm), Table S5, program 1.

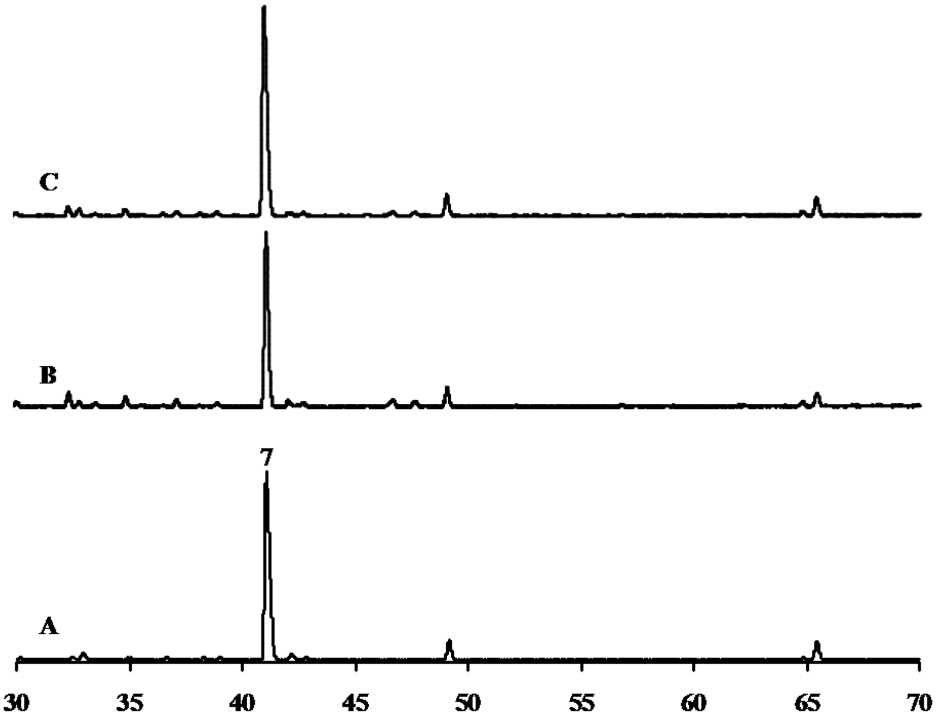

Figure S28: Chiral GC traces of SaSQS1 and SaSQS2 assay sample on Astec CHIRAL DEX^{TM} B-DA Capillary Column (30m × 0.25mm × 0.25um), A) SaSQS1 assay sample, B) SaSQS2 assay sample, C) Co-injection of SaSQS1 and SaSQS2 assay sample, Table S5 program 2.

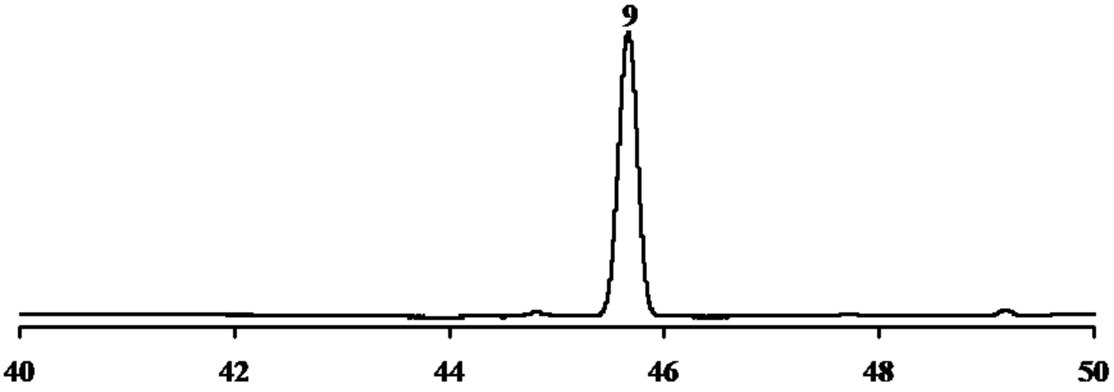

Figure S29: GC analysis of SaBS assay sample using HP-Chiral column, (30m × 0.32mm × 0.25um), (*S*)-β-bisabolene[9] (**9**), Table S5 program 4.

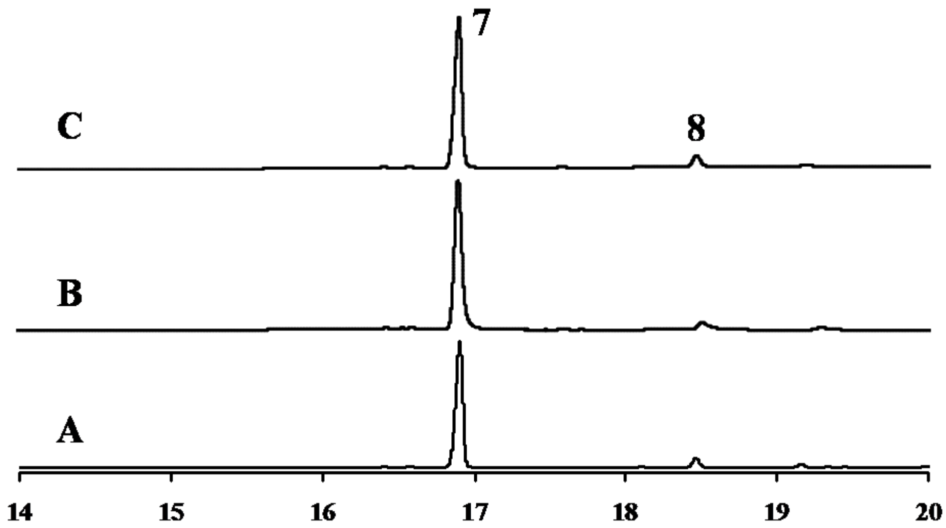

Figure S30-I. GC traces of SaSQS1 enzymatic assay product with (**A**) FPP, (**B**) GPP and IPP (1:1 molar ratio) pre-incubated with SaFDS, (**C**) DMAPP and IPP (1:2 molar ratio) pre-incubated with SaFDS; Peak identification: Sesquisabinene (**7**), and β-sesquiphellandrene (**8**). GC analysis was performed with HP-5 capillary column (30m × 0.32mm × 0.25µm), Table S5 program 1.

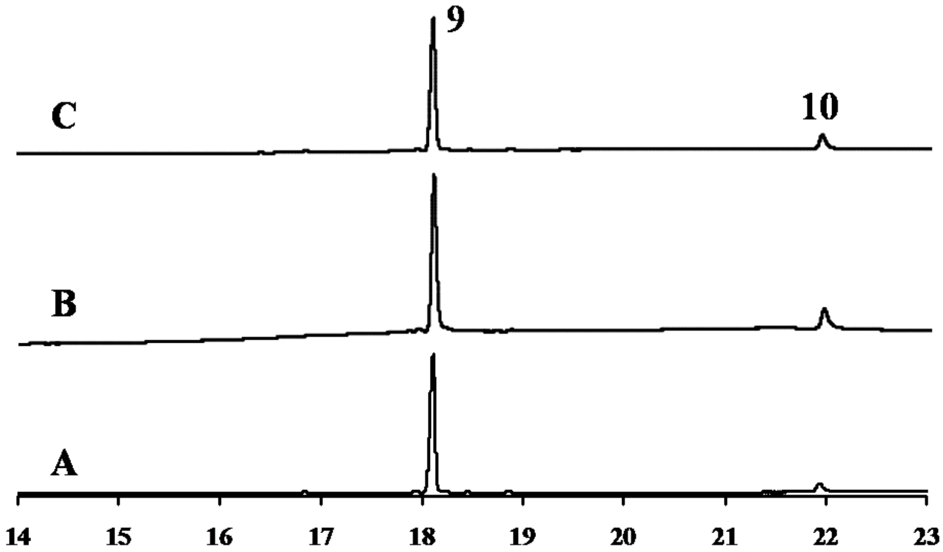

Figure S30-II. GC traces of SaBS enzymatic assay product with, **A**) FPP, **B**) GPP and IPP (1:1 molar ratio) pre-incubated with SaFDS, **C**) DMAPP and IPP (1:2 molar ratio) pre-incubated with SaFDS; Peak identification: (*S*)-β-bisabolene (**9**), and α-bisabolol (**10**). GC analysis was performed with HP-5 capillary column (30m × 0.32mm × 0.25µm), Table S5 program 1.

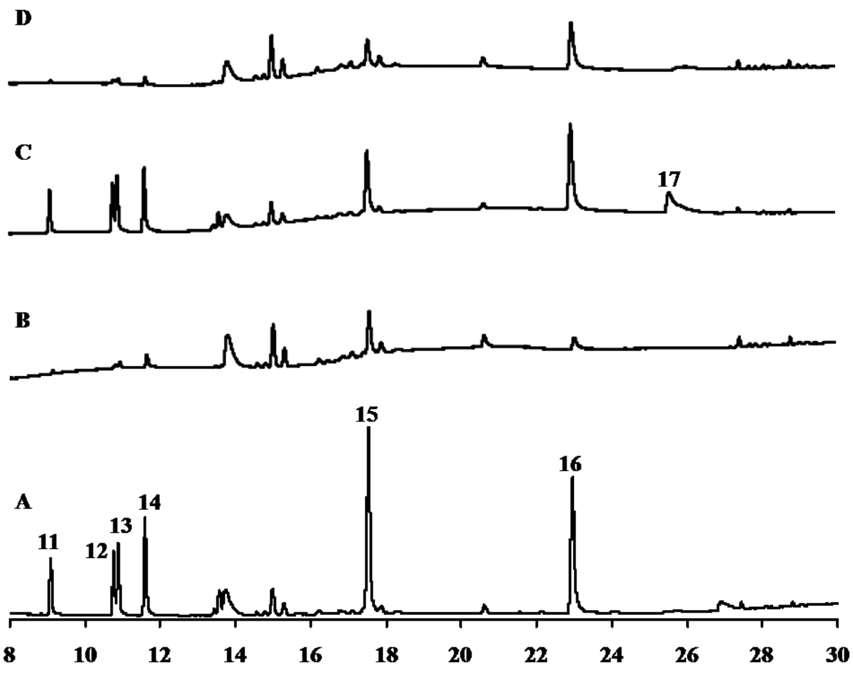

Figure S31: GC analysis of products of, **A**) Product profile of SaSQS1 assay with GPP, **B**) Product profile of SaSQS2 assay with GPP, **C**) Product profile of SaSQS1 assay with NPP, **D**) Product profile of SaSQS2 assay with NPP.

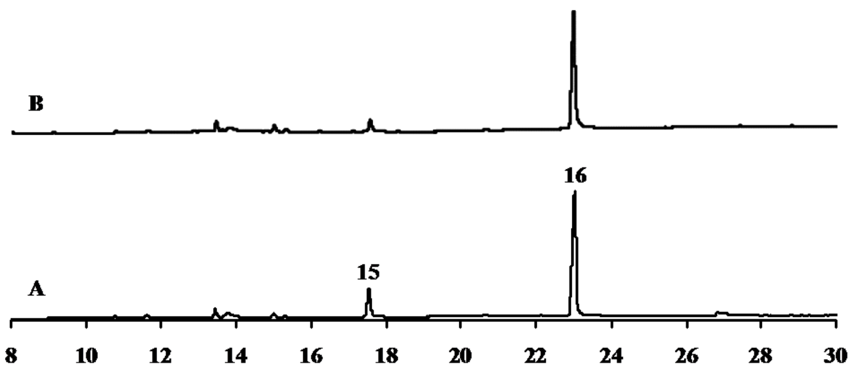

Figure S32: **A**) GC analysis of product profile of SaBS assay with GPP, **B**) Product profile of SaBS assay with NPP.

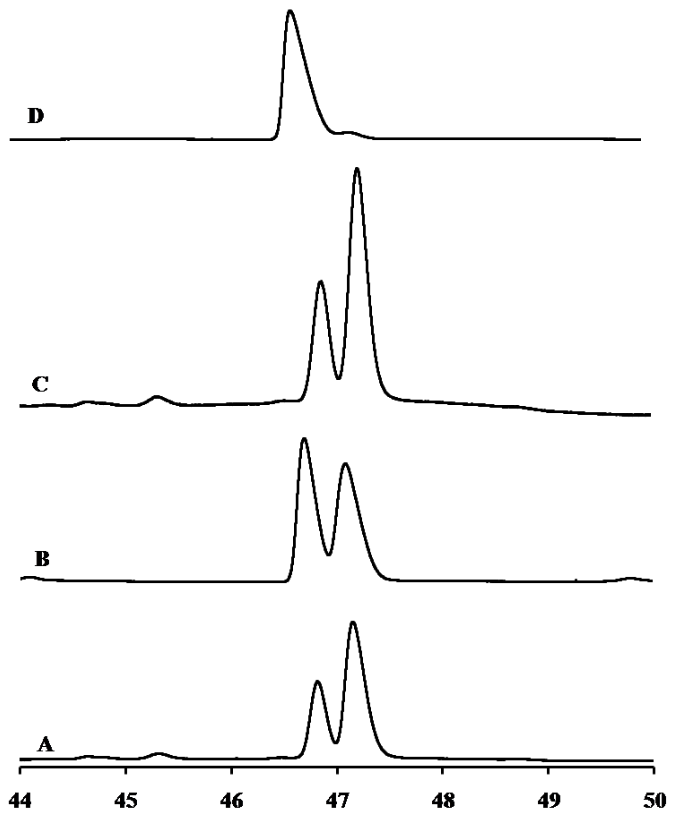

Figure S33: GC analysis of SaSQS1 assay sample with GPP using HP-Chiral column, (30m × 0.32mm × 0.25um), **A**) Product profile of SaSQS1 assay with GPP on HP5 chiral column, **B**) Linalool standard (racemic mixture), **C)** Co-injection of SaSQS1 assay sample with racemic mixture of linalool, **D**) (-)-linalool.

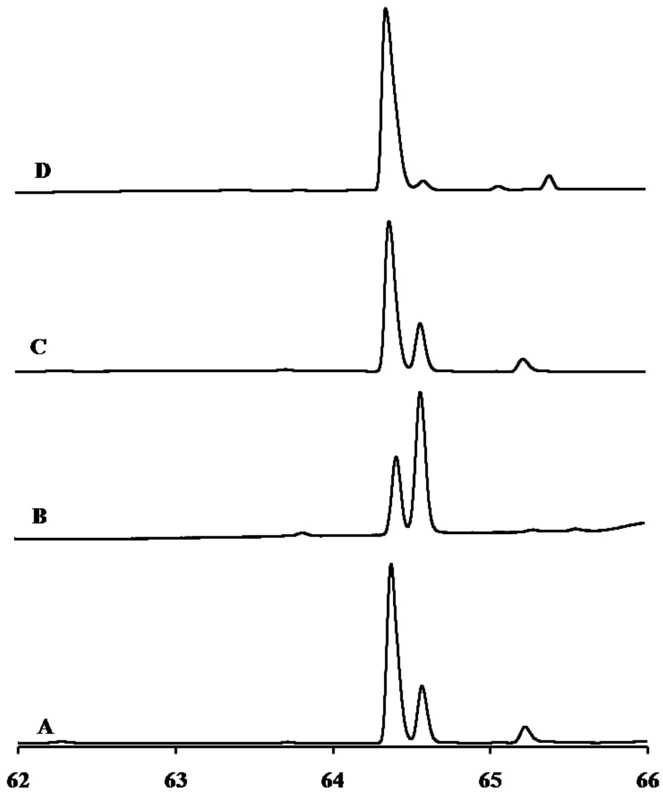

Figure S34: GC analysis of SaSQS1 and SaBS assay sample with GPP using HP-Chiral column, (30m × 0.32mm × 0.25μm), **A**) Product profile of SaSQS1 assay with GPP on HP chiral column, **B**) α-terpineol standard, **C)** Co-injection of SaSQS1 assay sample with α-terpineol, **D**) Product profile of SaBS assay with GPP on HP chiral column.

Section IV: GC-MS fragmentation of identified terpenes:

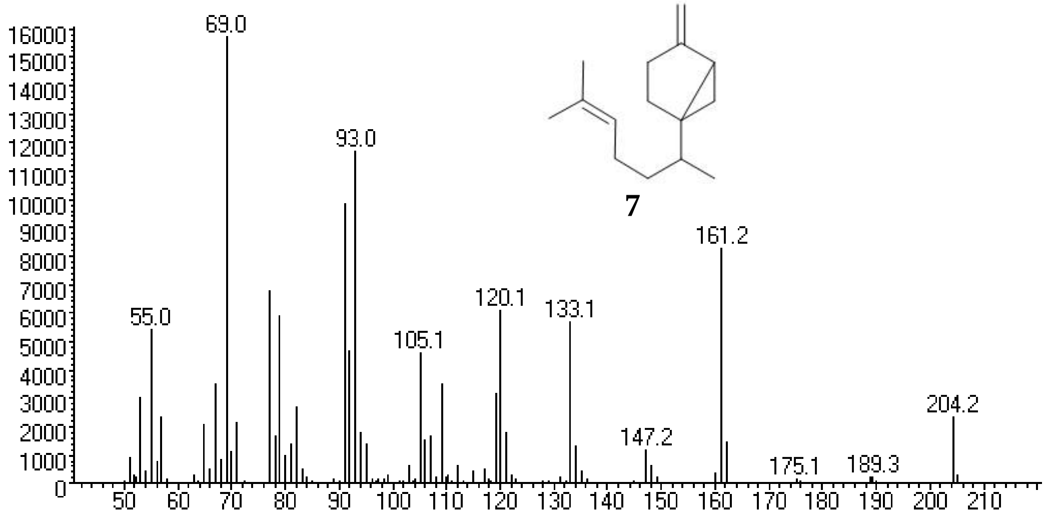

Figure S35: EI Mass Spectrum of Sesquisabinene (**7**) produced by incubation of SaSQS1 with (*E*,*E*)-FPP.

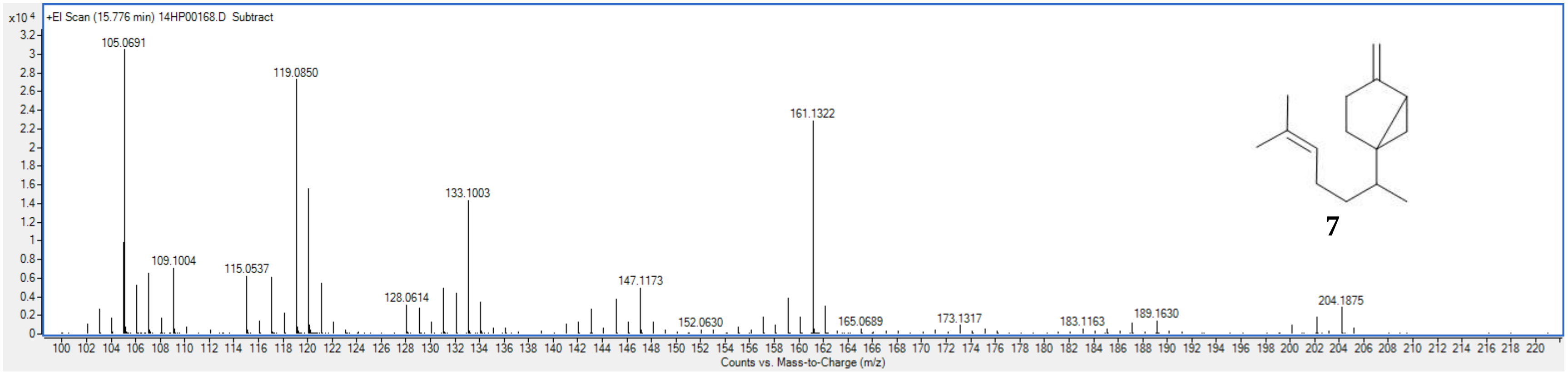

Figure S36: GC-HR-EI Mass Spectrum of Sesquisabinene (**7**) produced by incubation of SaSQS1 with (*E*,*E*)-FPP.

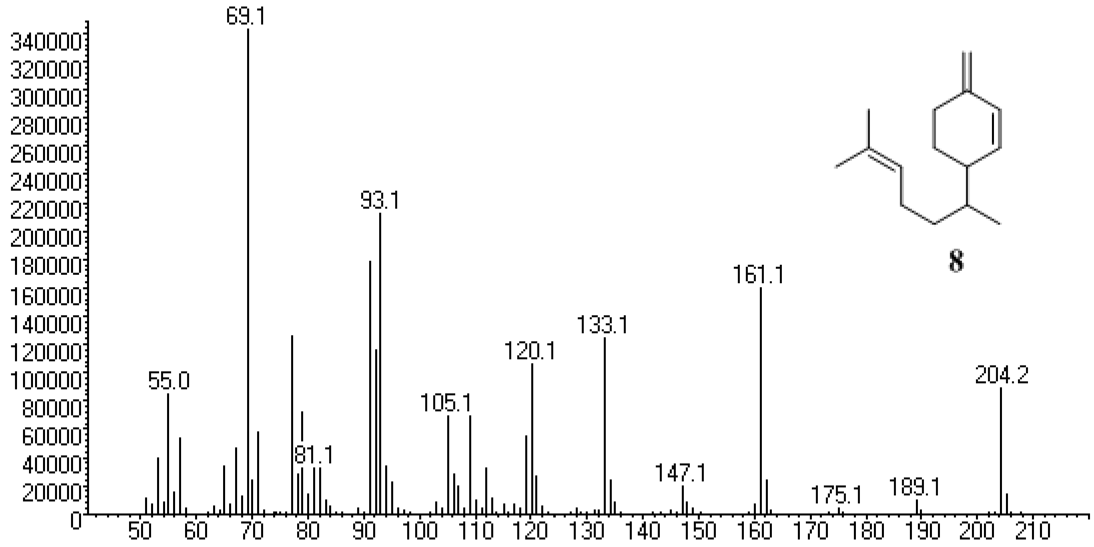

Figure S37: EI Mass spectrum of Sesquiphellandrene (**8**) produced by incubation of SaSQS1 with (*E*,*E*)-FPP.

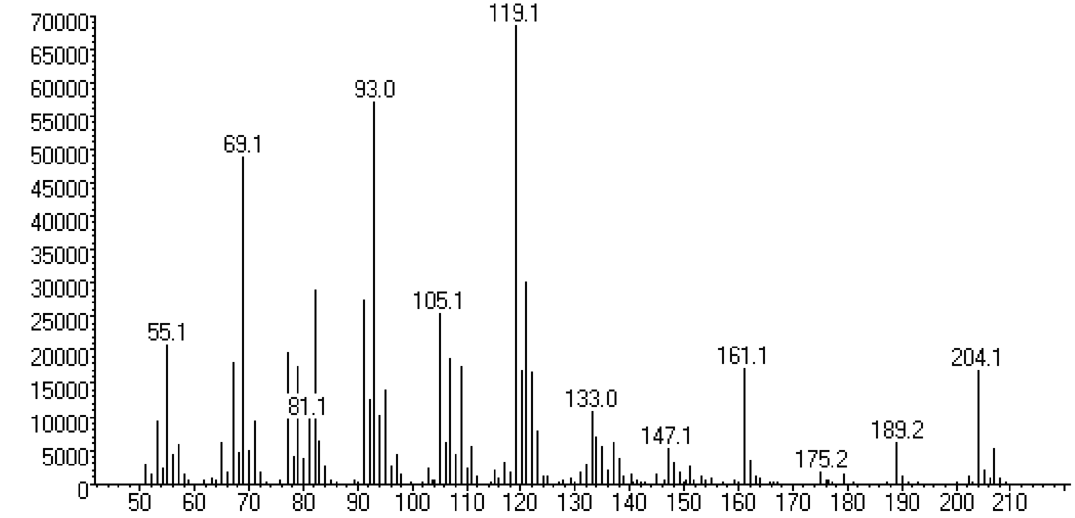

Figure S38: EI Mass spectrum of unknown peak at RT 19.25 min produced by incubation of SaSQS1 with (*E*,*E*)-FPP.

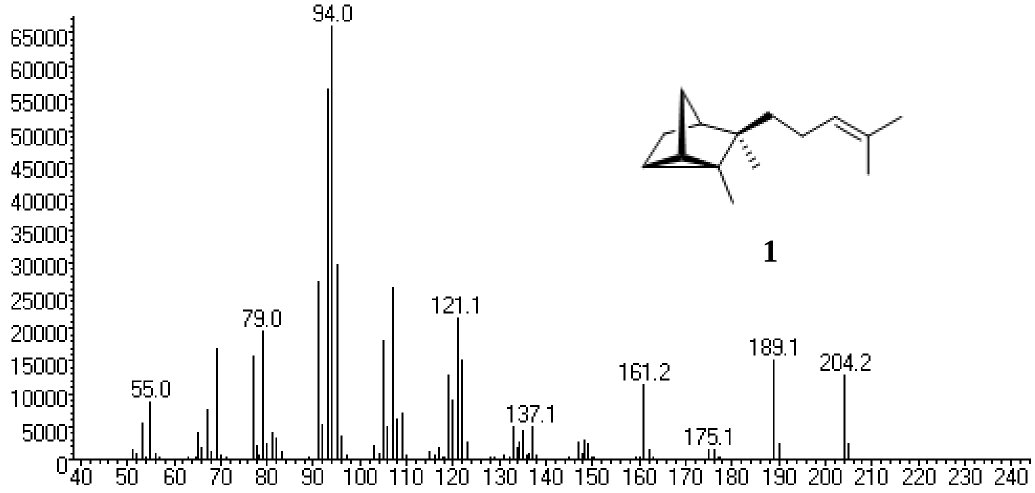

Figure S39: EI Mass Spectrum of α-Santalene (**1**) produced by incubation of SaSS with (*E*,*E*)-FPP.

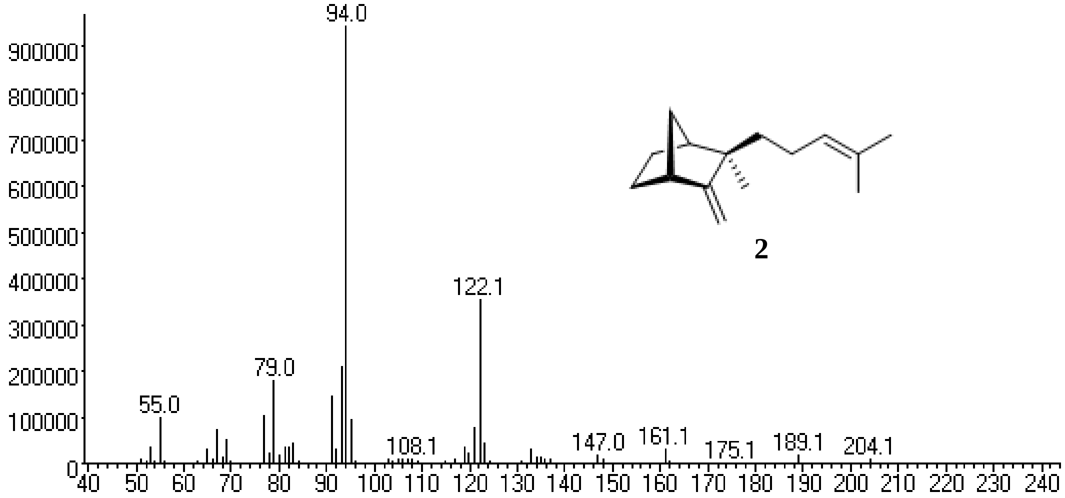

Figure S40: EI Mass Spectrum of β-Santalene (**2**) produced by incubation of SaSS with (*E*,*E*)-FPP.

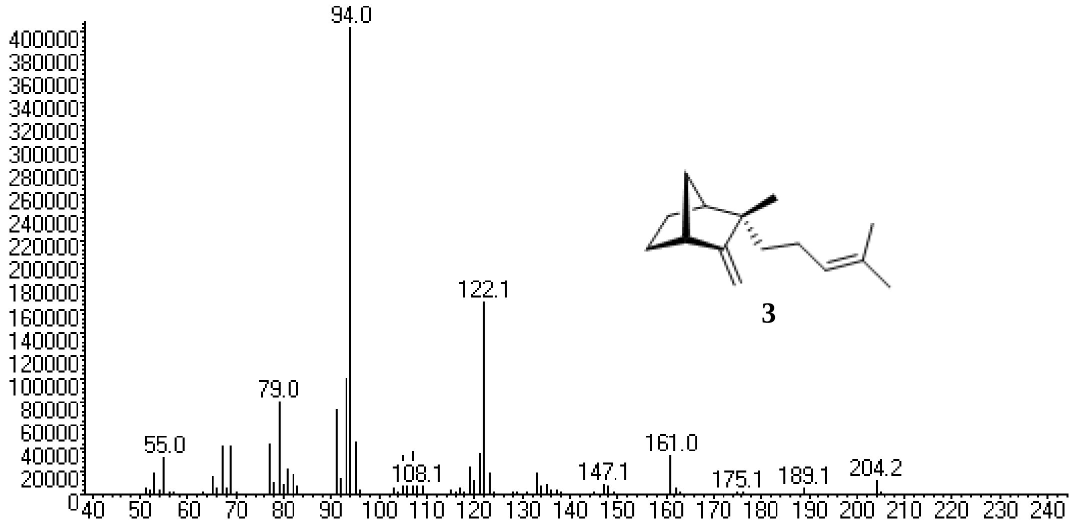

Figure S41: EI Mass Spectrum of *epi*-β-Santalene (**3**) produced by incubation of SaSS with (*E*,*E*)-FPP.

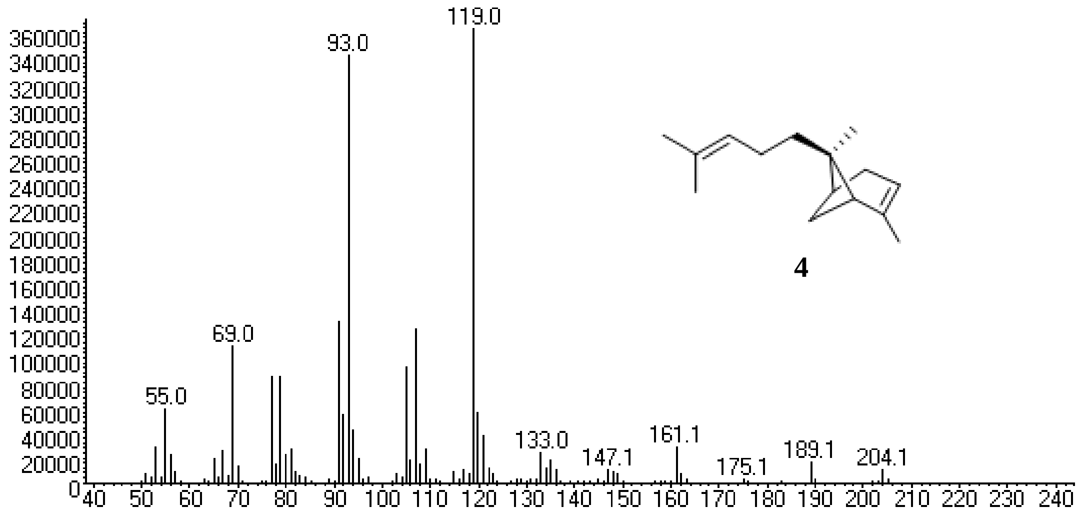

Figure S42: EI Mass Spectrum of *exo*-α-Bergamotene (**4**) produced by incubation of SaSS with (*E*,*E*)-FPP.

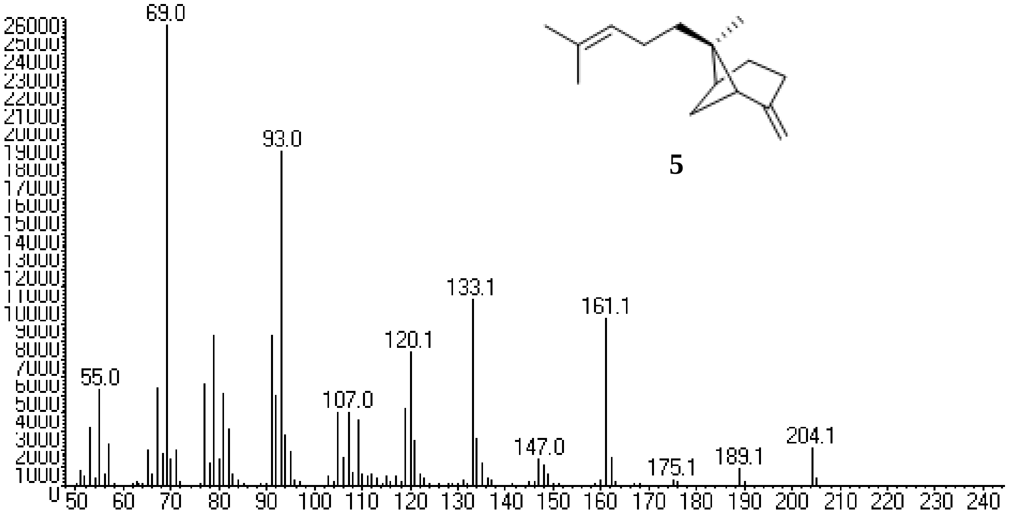

Figure S43-A: EI Mass Spectrum of *exo*-β-Bergamotene (**5**) produced by incubation of SaSS with (*E*,*E*)-FPP.

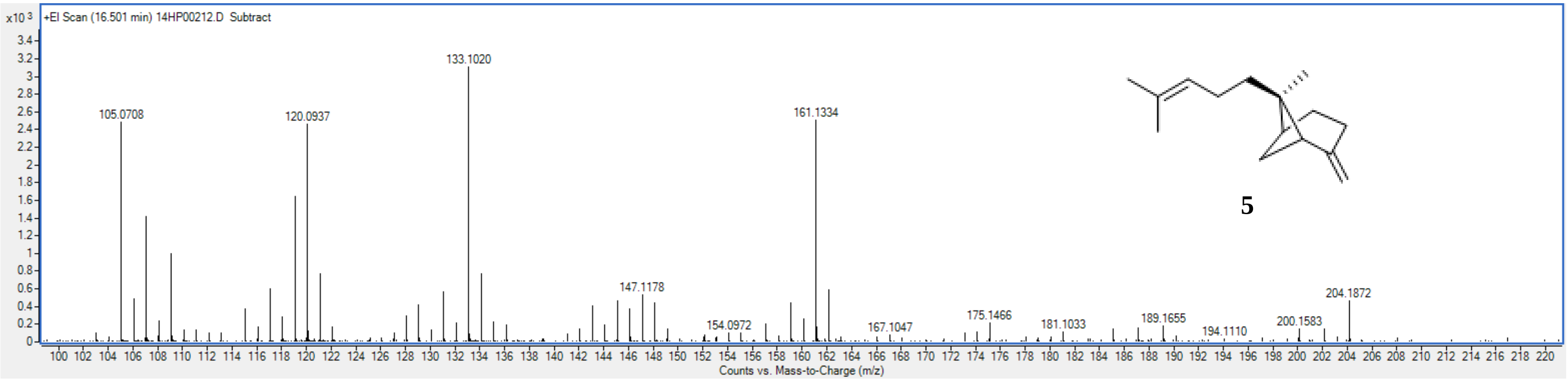

Figure S43-B: GC-HR-EI Mass Spectrum of *exo*-β-bergamotene (**5**) produced by incubation of SaSS with (*E*,*E*)-FPP.

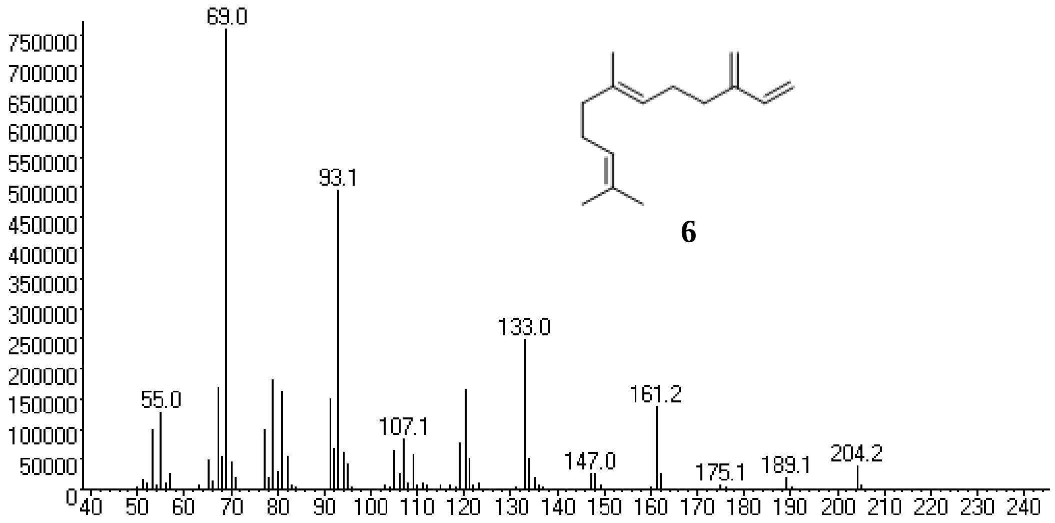

Figure S44: EI Mass Spectrum of (*E*)-β-Farnesene (**6**) produced by incubation of SaSS with (*E*,*E*)-FPP.

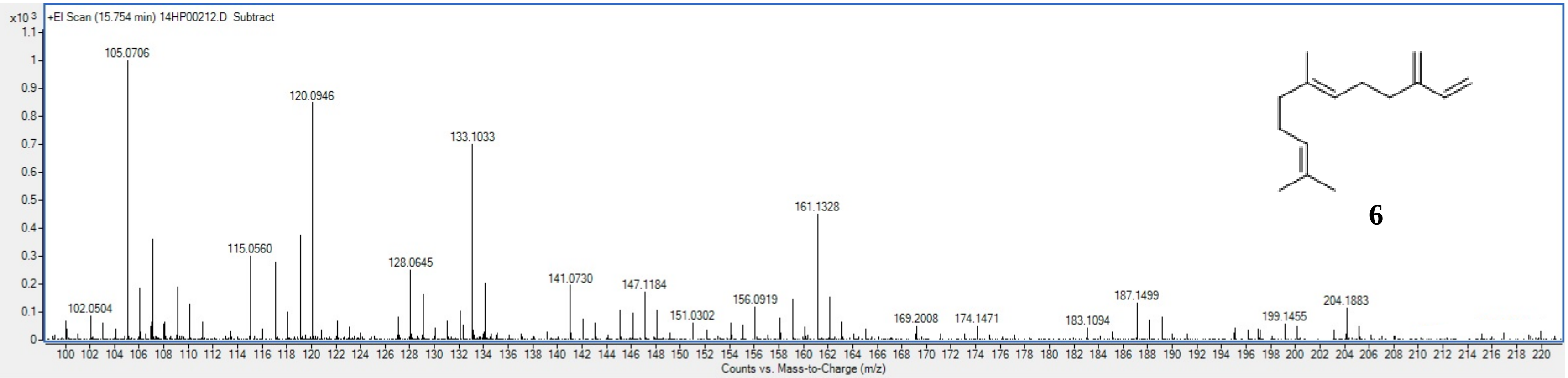

Figure S45: GC-HR-EI mass spectrum of (*E*)-β-Farnesene (**6**) produced by incubation of SaSS with (*E*,*E*)-FPP.

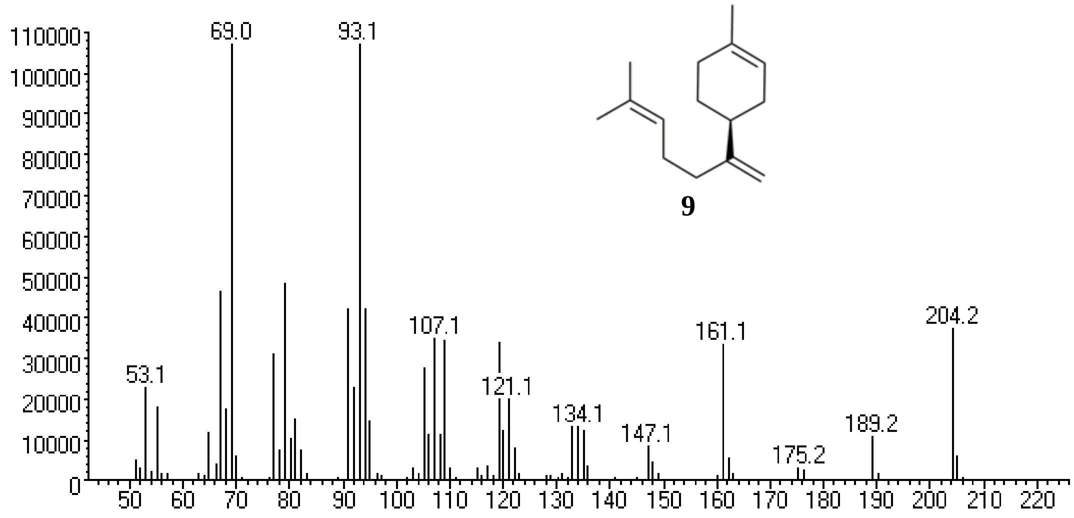

Figure S46: EI Mass spectrum of (*S*)-β-Bisabolene (**9**) produced by incubation of SaBS with (*E*,*E*)-FPP.

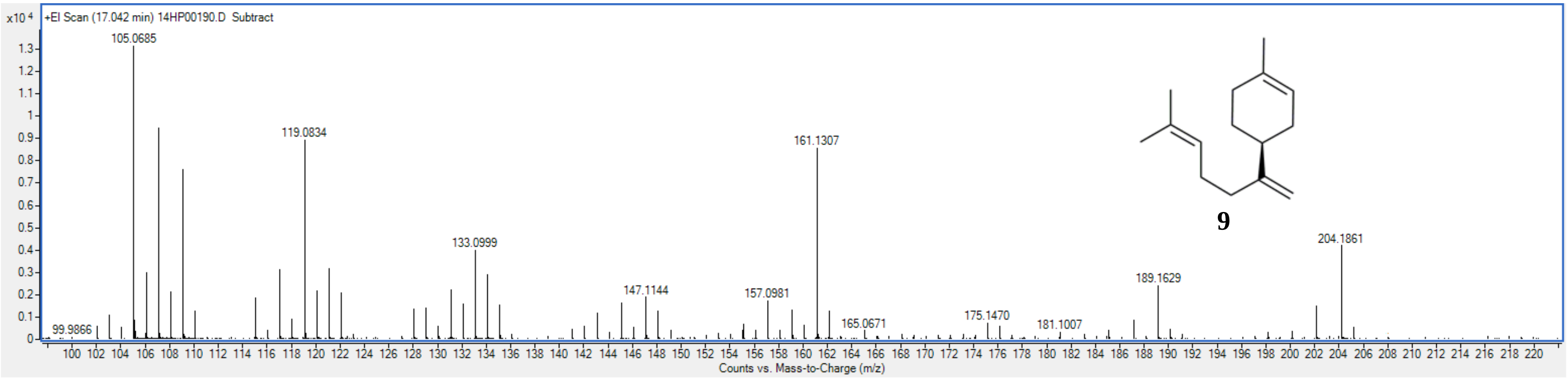

Figure S47: GC-HR-EI mass spectrum of (*S*)-β-Bisabolene (**9**) produced by incubation of SaBS with (*E*,*E*)-FPP.

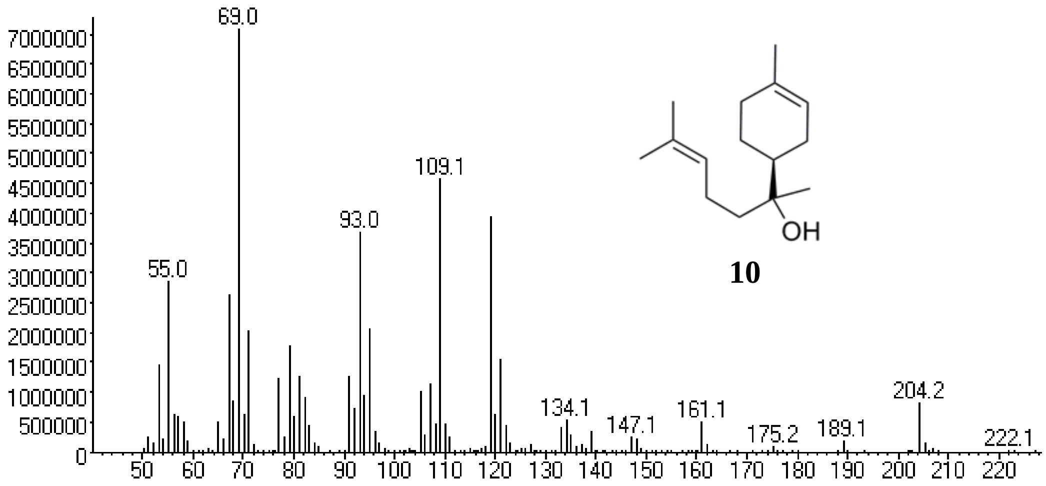

Figure S48: EI Mass spectrum of α-Bisabolol (**10**) produced by incubation of SaBS with (*E*,*E*)-FPP.

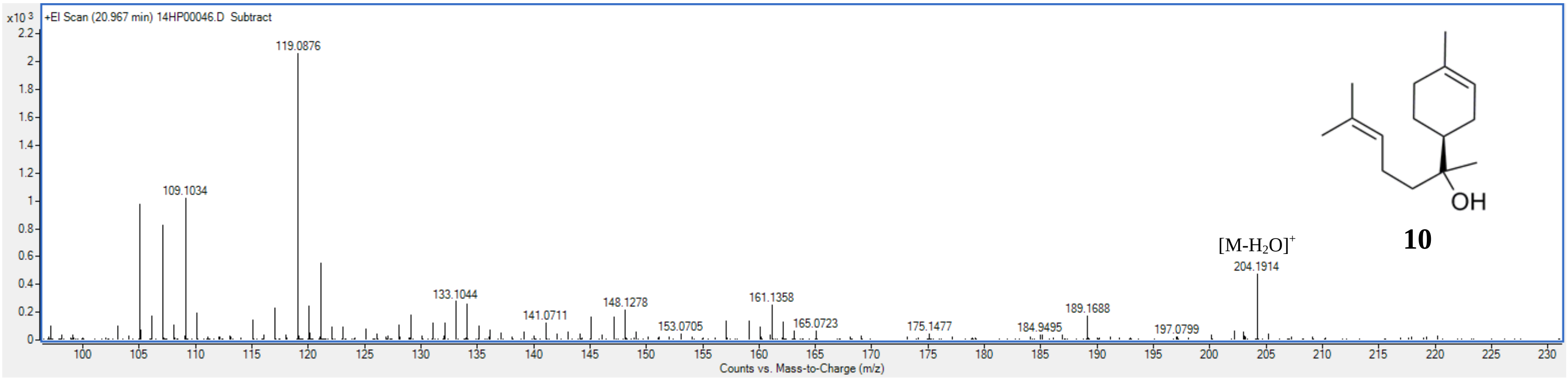

Figure S49: GC-HR-EI mass spectrum of α-Bisabolol (**10**) produced by incubation of SaBS with (*E*,*E*)-FPP.

Section V: Characterization of isolated sesquiterpenes by NMR:

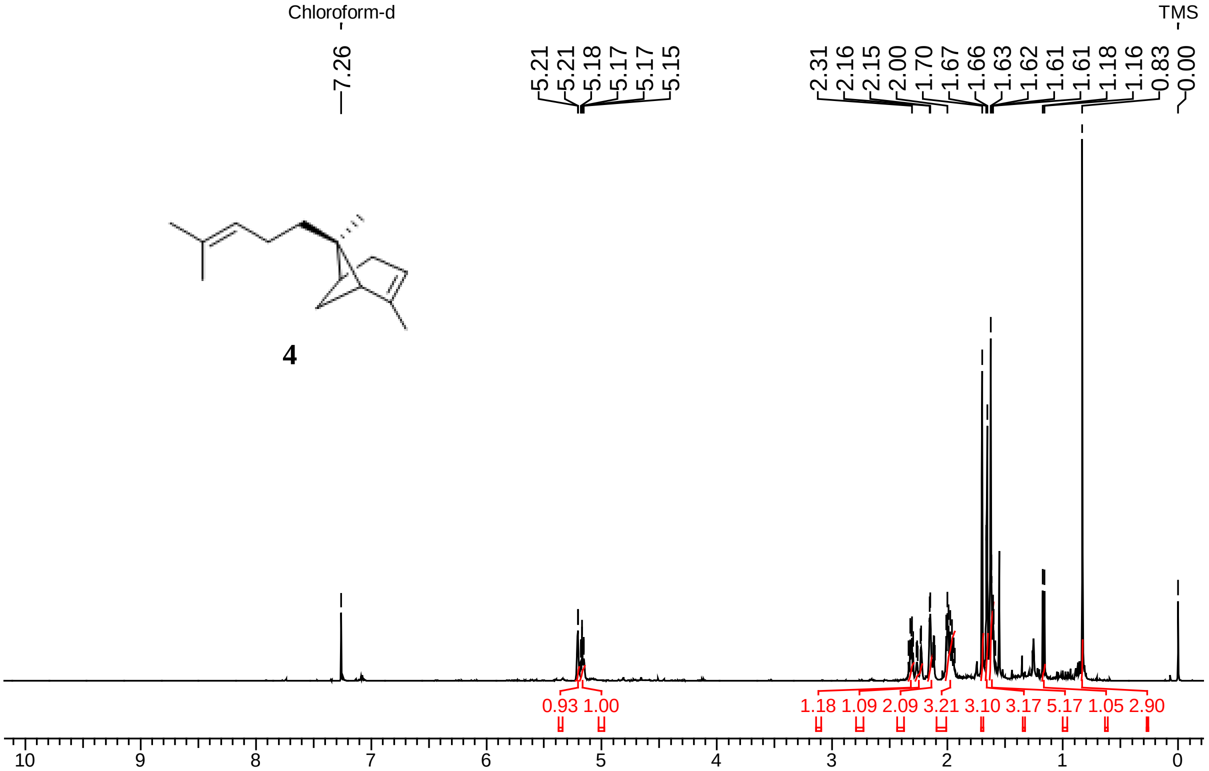

Figure S50: ^{1}H NMR of *exo*-α-Bergamotene (**4**) in $CDCl_3$ at 500 MHz.

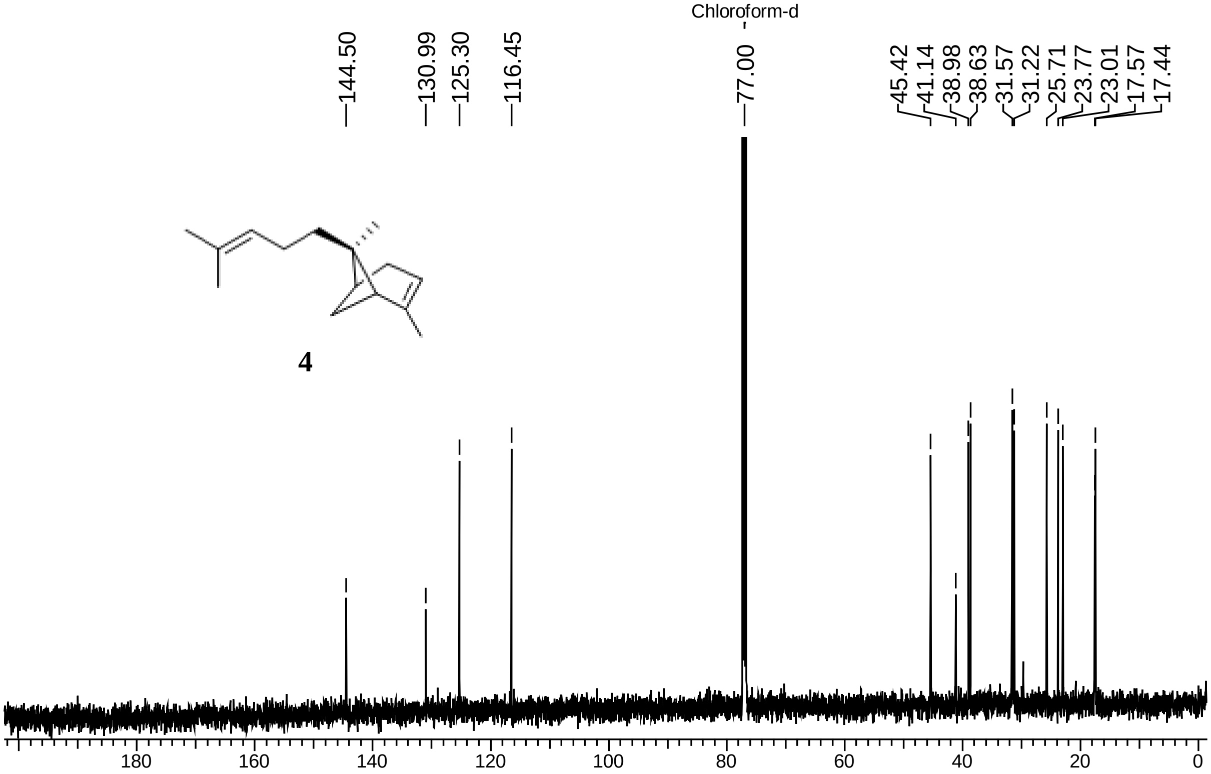

Figure S51: ^{13}C NMR of *exo*-α-Bergamotene (**4**) in $CDCl_3$ at 125 MHz.

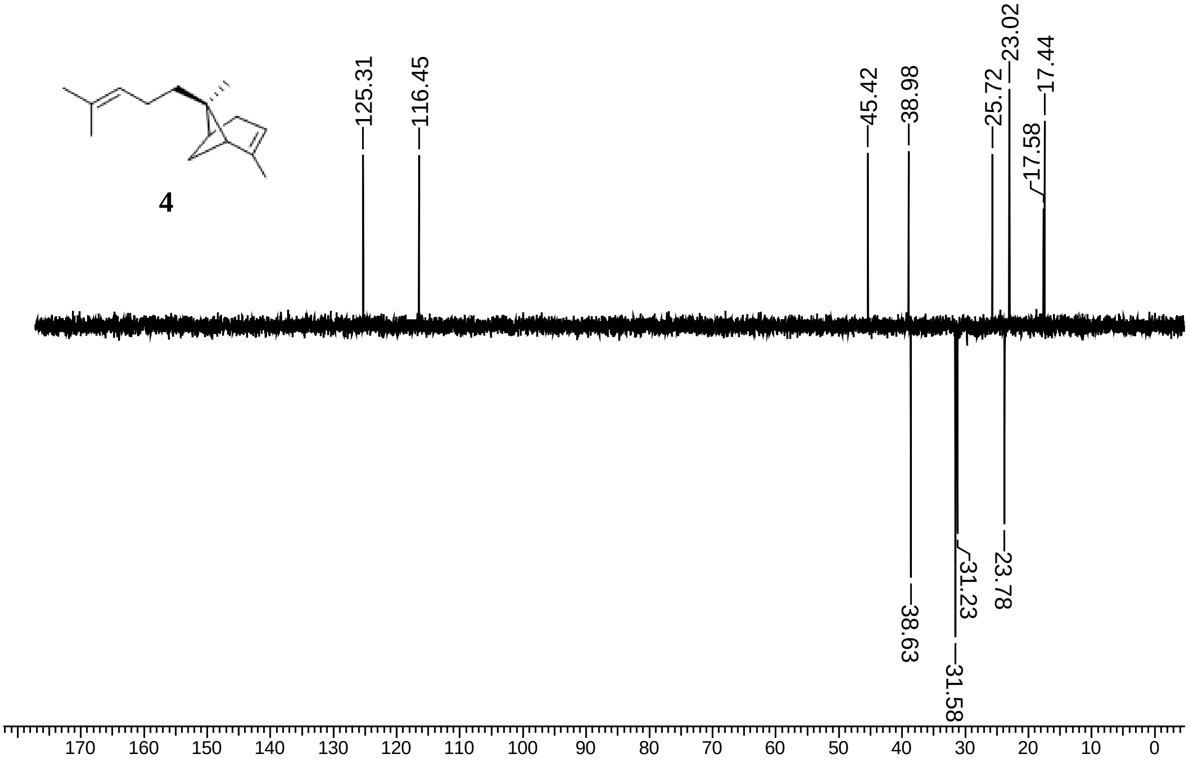

Figure S52: DEPT NMR of *exo*-α-Bergamotene (**4**) in $CDCl_3$ at 125 MHz.

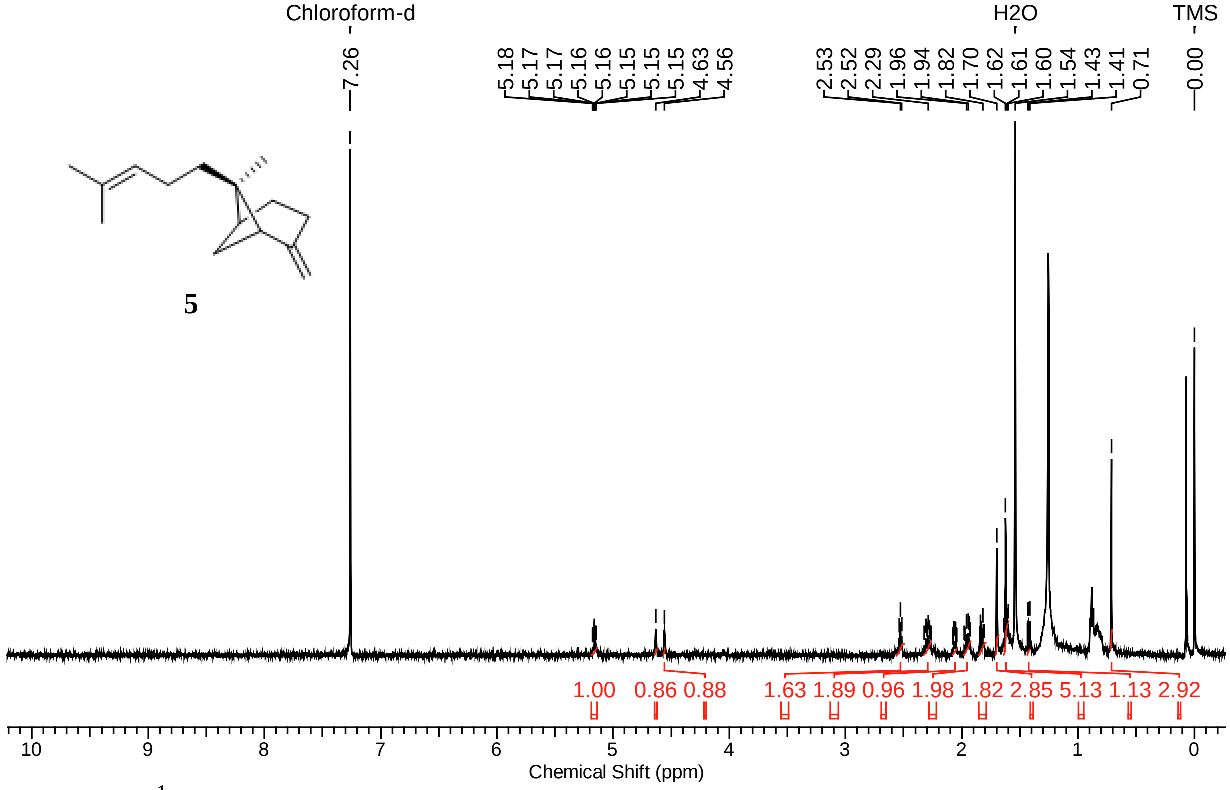

Figure S53: ^{1}H NMR of *exo*-β-Bergamotene (**5**) in $CDCl_3$ at 500 MHz.

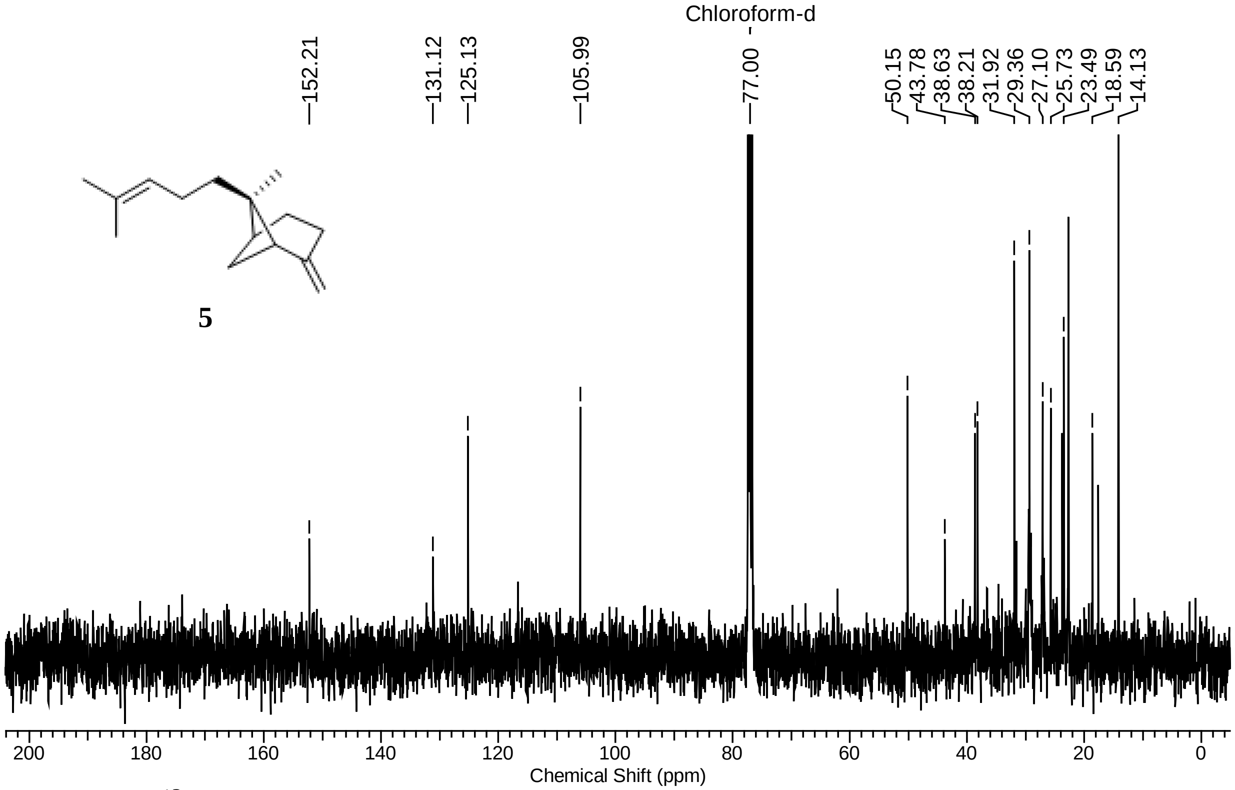

Figure S54: ^{13}C NMR of *exo*-β-Bergamotene (**5**) in $CDCl_3$ at 125 MHz.

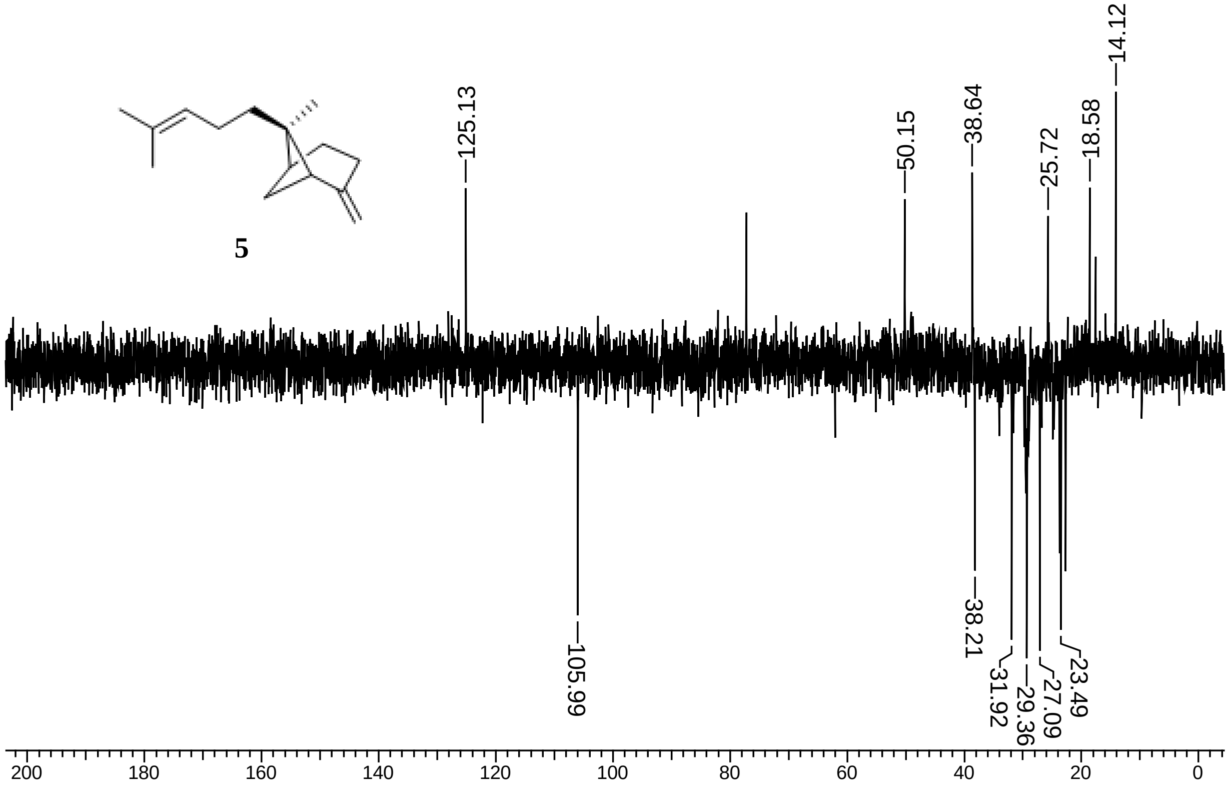

Figure S55: DEPT NMR of *exo*-β-Bergamotene (**5**) in $CDCl_3$ at 125 MHz.

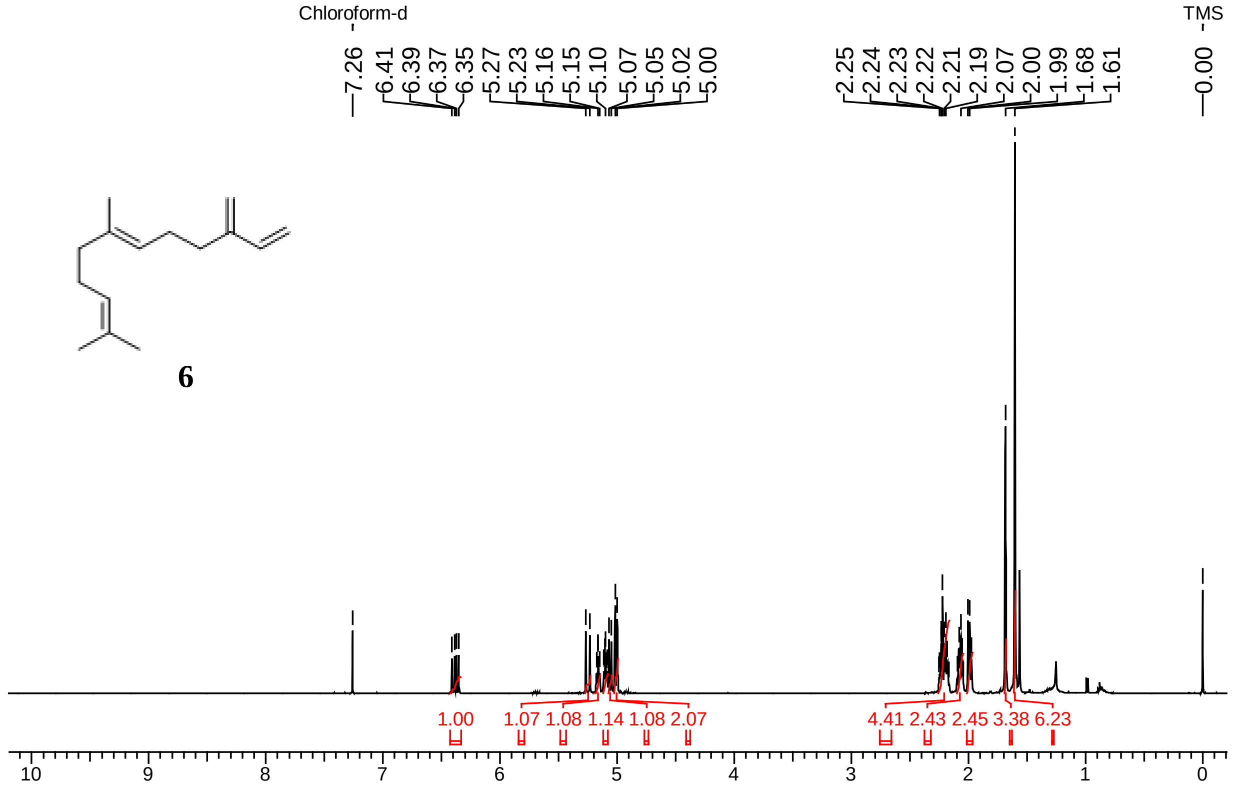

Figure S56: ^{1}H NMR of (*E*)-β-Farnesene (**6**) in $CDCl_3$ at 500 MHz.

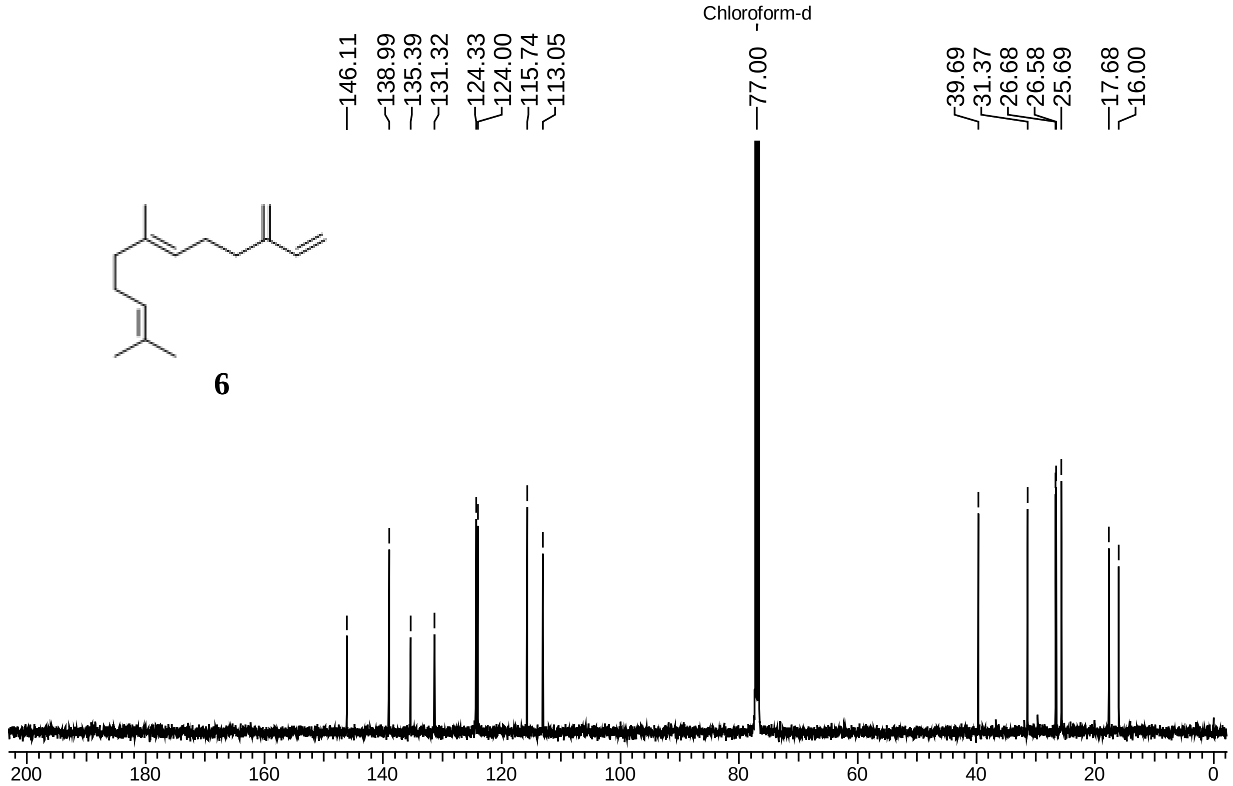

Figure S57: ^{13}C NMR of (*E*)-β-Farnesene (**6**) in $CDCl_3$ at 125 MHz.

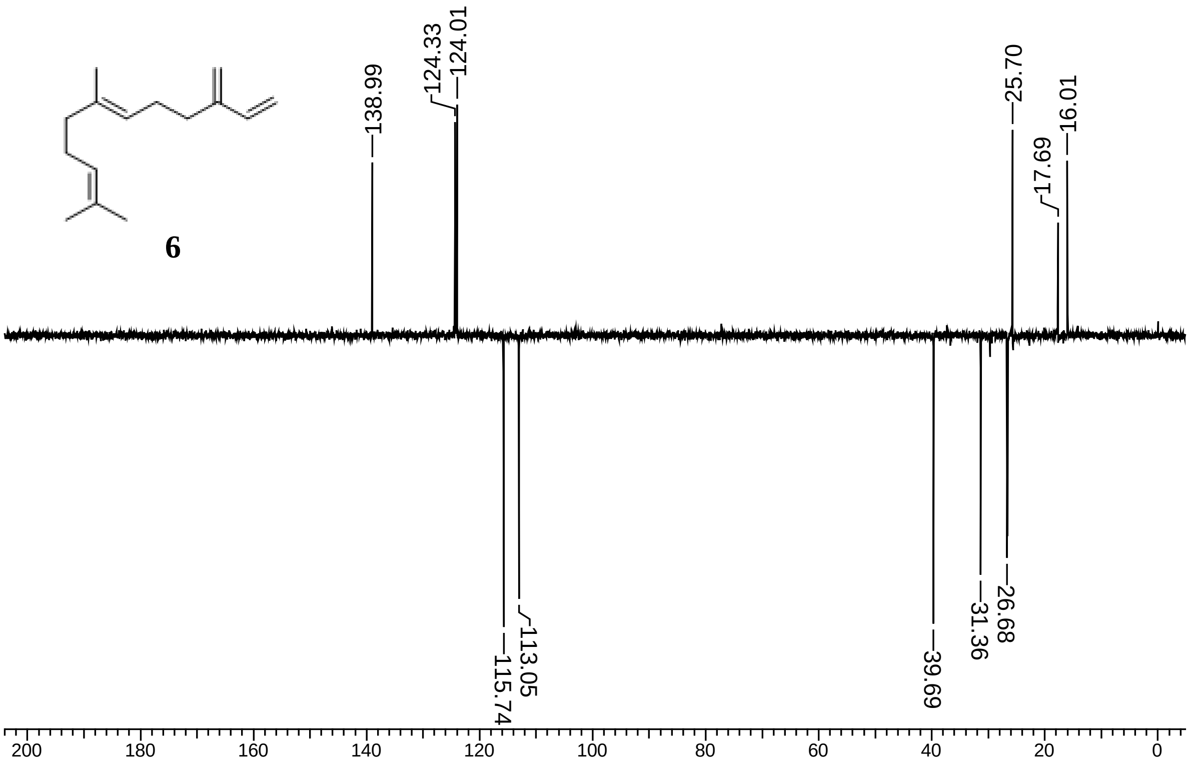

Figure S58: DEPT NMR of (*E*)-β-Farnesene (**6**) in $CDCl_3$ at 125 MHz.

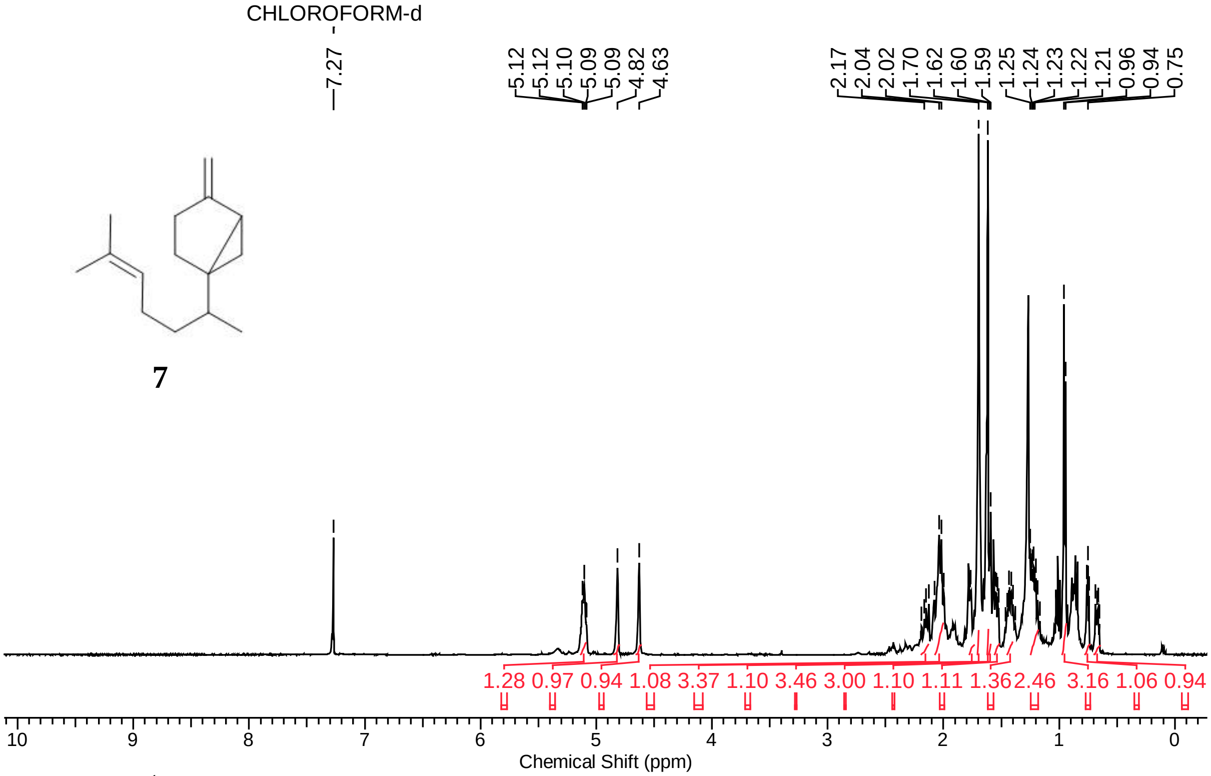

Figure S59: ^{1}H NMR of Sesquisabinene (**7**) in $CDCl_3$ at 400 MHz.

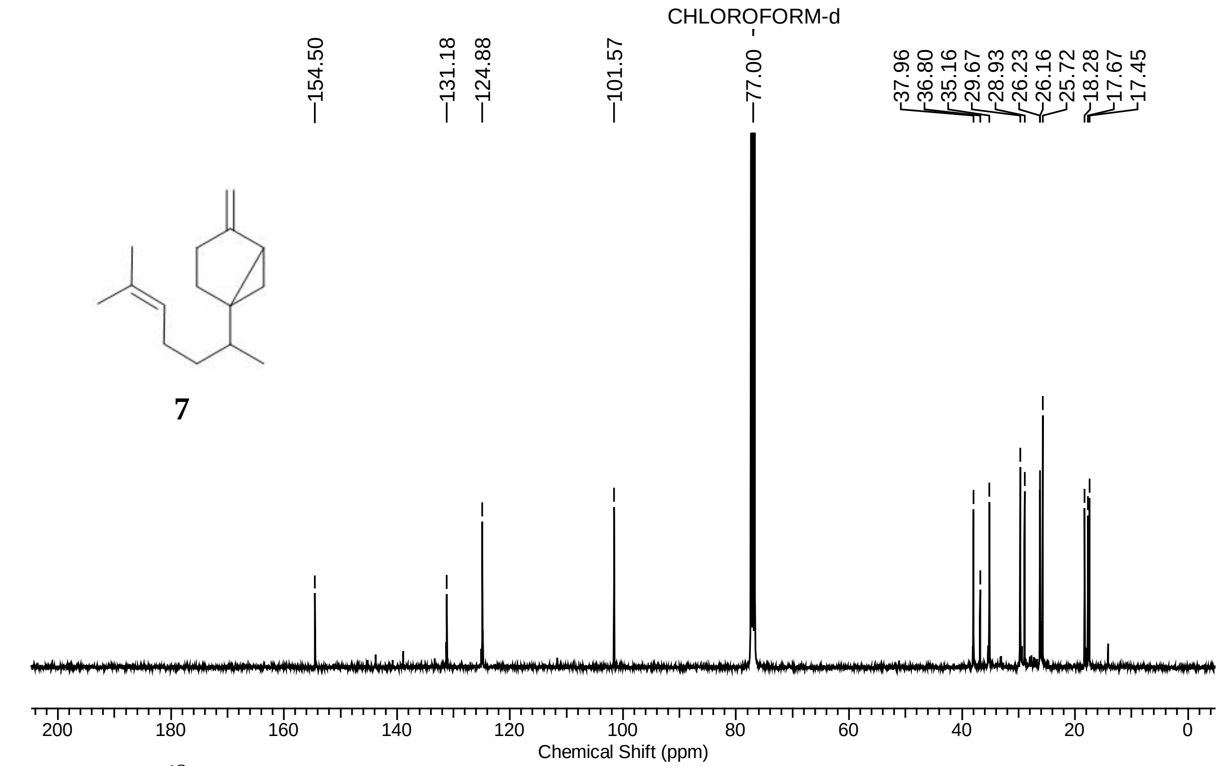

Figure S60: ^{13}C NMR of Sesquisabinene (**7**) in $CDCl_3$ at 100 MHz.

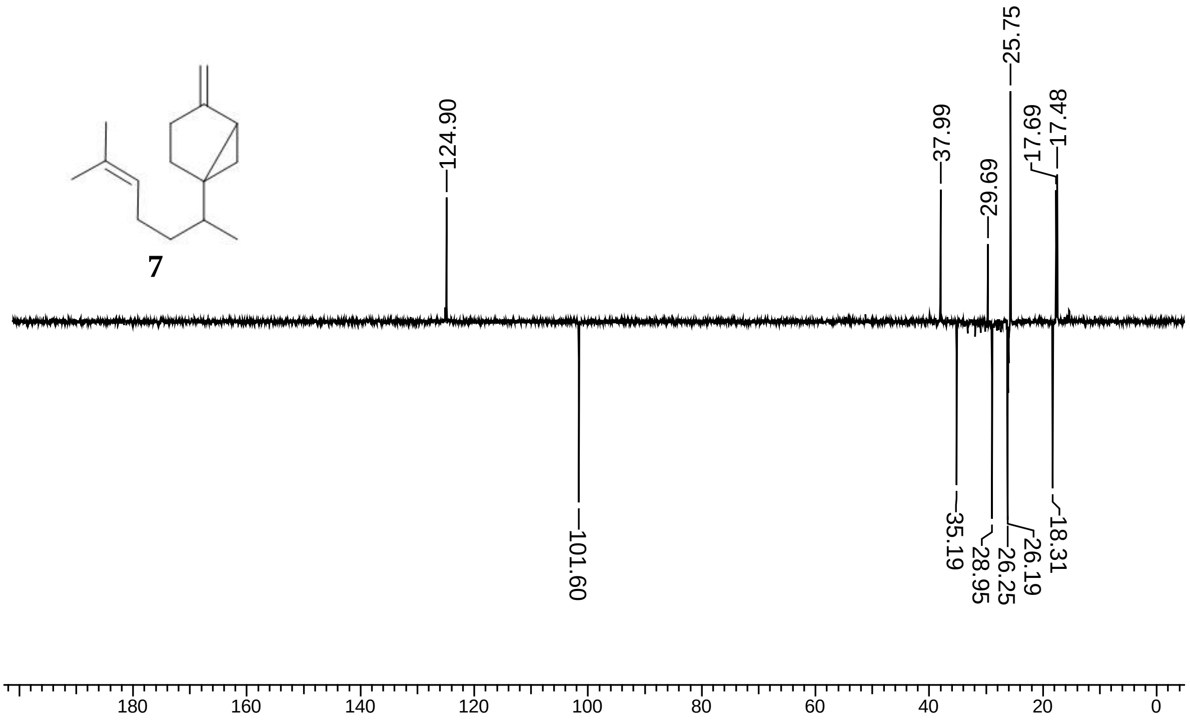

Figure S61: DEPT NMR of Sesquisabinene (**7**) in $CDCl_3$ at 100 MHz.

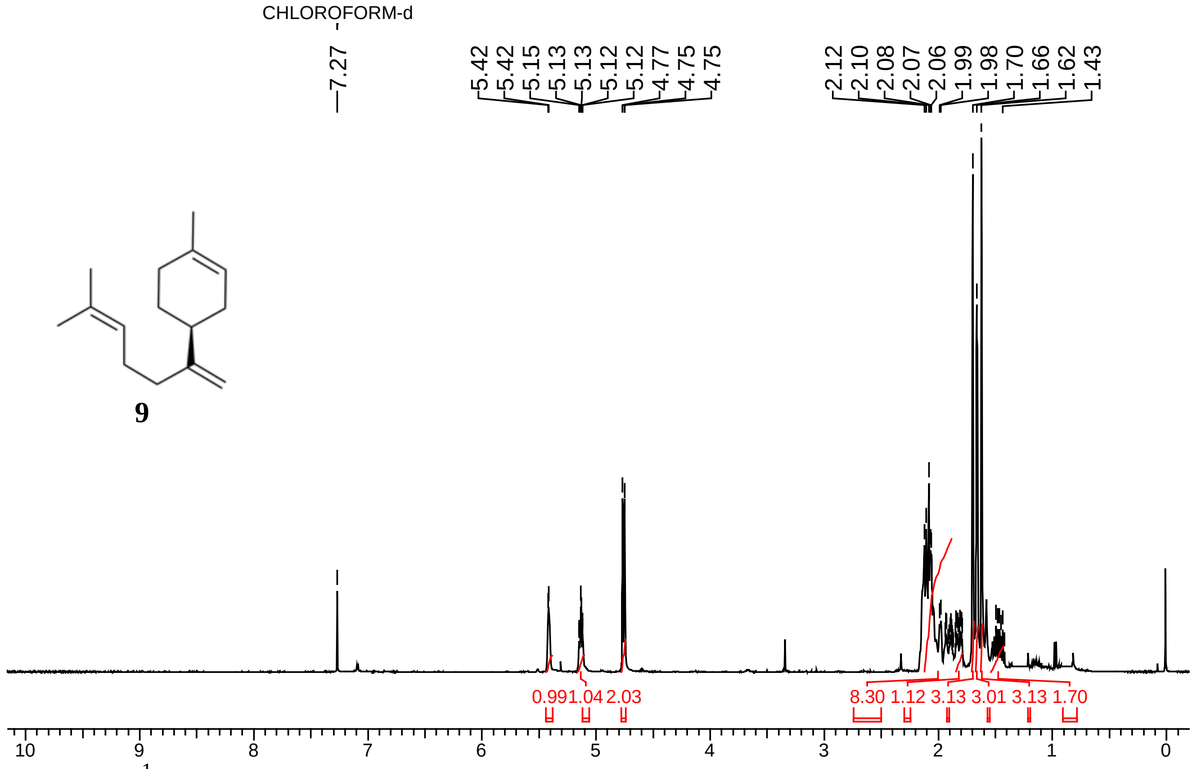

Figure S62: ^{1}H NMR of (*S*)-β-Bisabolene (**9**) in $CDCl_3$ at 400 MHz.

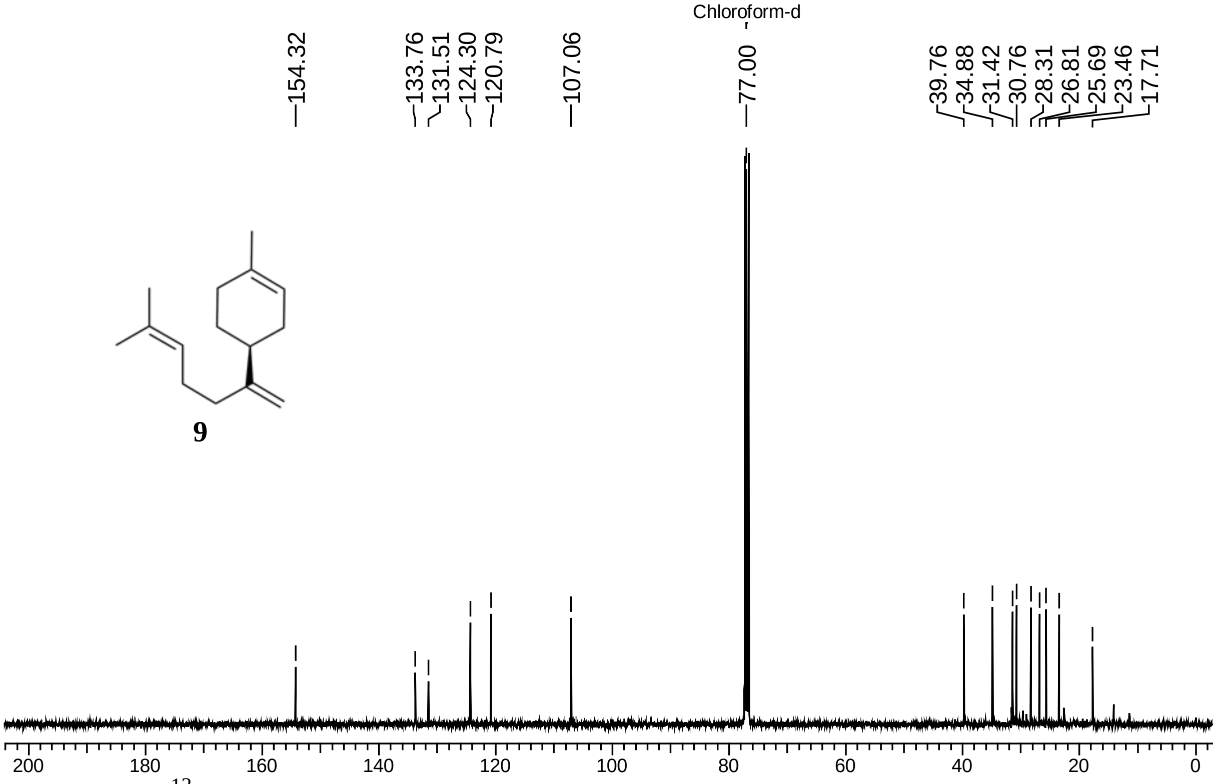

Figure S63: ^{13}C NMR of (*S*)-β-Bisabolene (**9**) in $CDCl_3$ at 100 MHz.

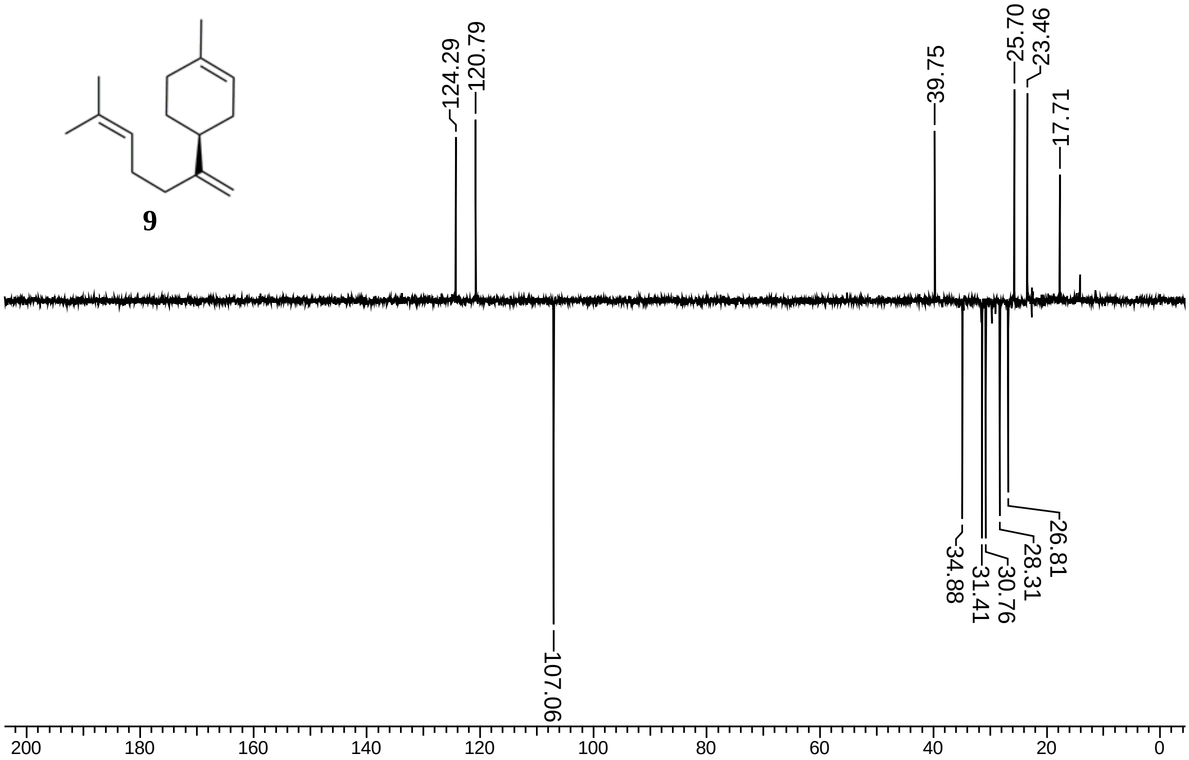

Figure S64: DEPT NMR of (*S*)-β-Bisabolene (**9**) in $CDCl_3$ at 100 MHz.

Section VI: Co-injection of standard terpenes with assay mixture

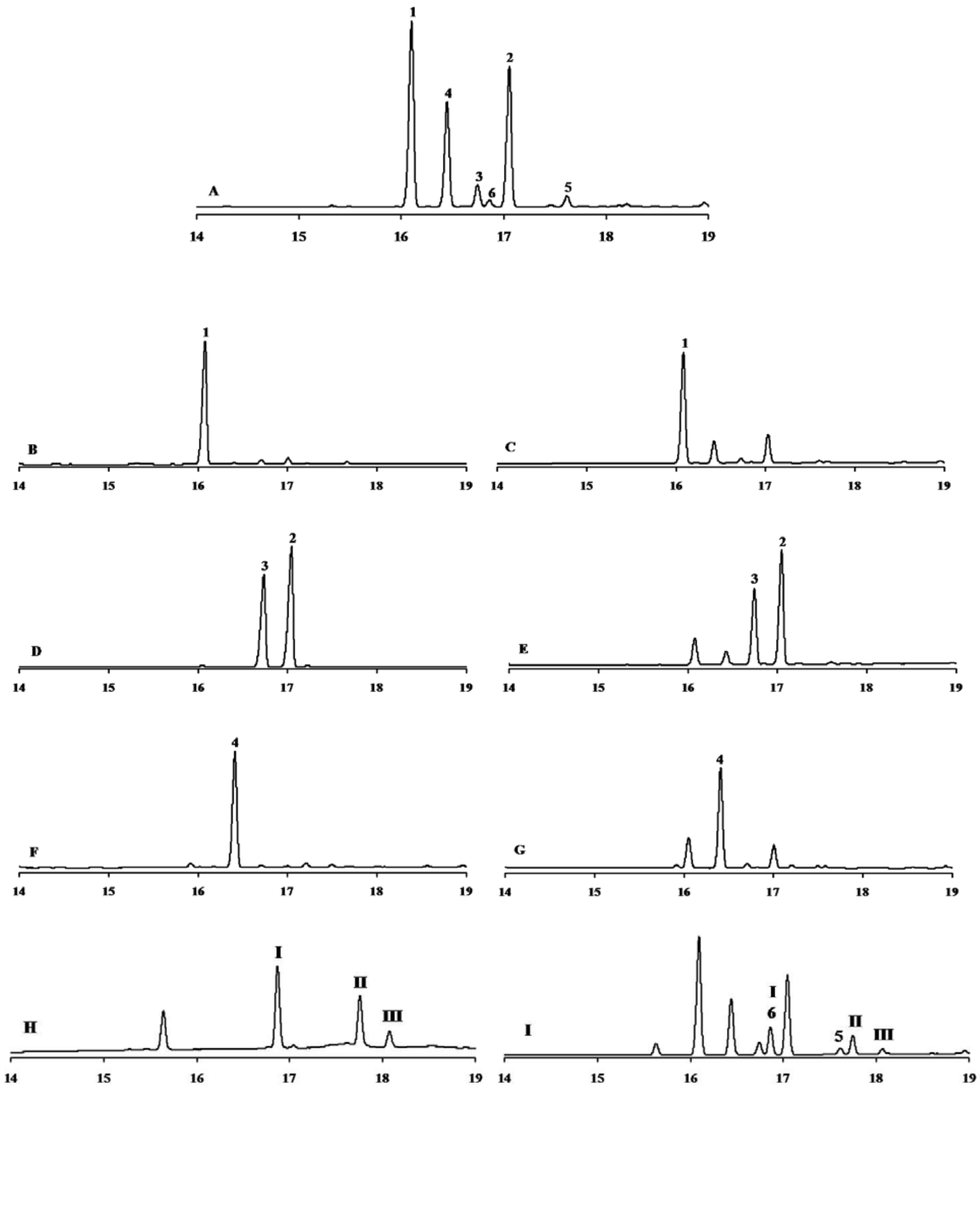

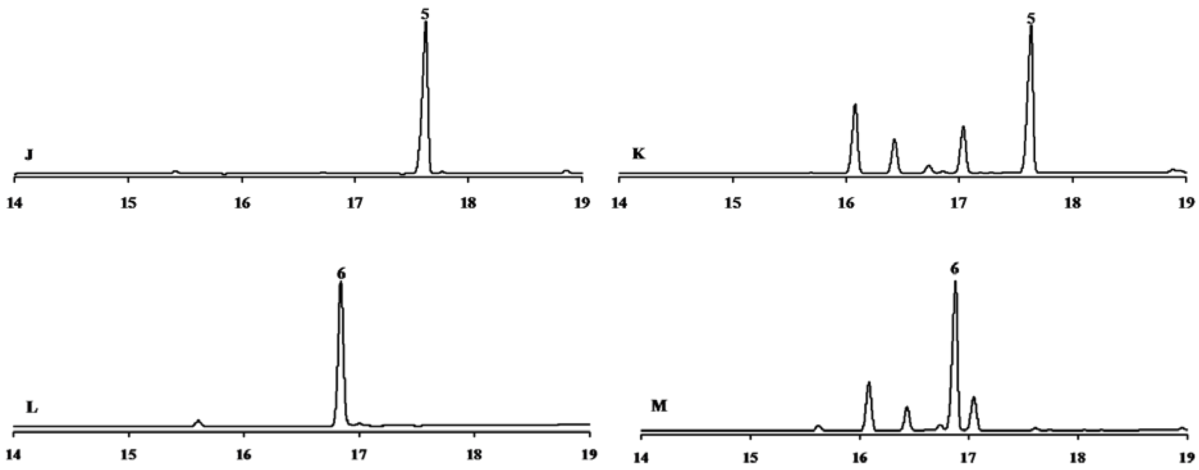

Figure S65: Co-injection of standard terpenes with SaSS assay mixture for the characterization of metabolites, **A)** Product profile of SaSS assay with (*E*,*E*)-FPP, **B)** α-Santalene (**1**) standard, **C)** Co-injection of SaSS assay mixture with α-Santalene, **D)** β-Santalene (**2**) and *epi*-β-Santalene (**3**), **E)** Co-injection of SaSS assay mixture with β-Santalene and *epi*-β-Santalene, **F)** *exo*-α-Bergamotene (**4**) standard, **G)** Co-injection of SaSS assay mixture with *exo*-α-Bergamotene, **H)** Synthesized mixture of (6*E*)-Farnesenes, **I)** Co-injection of SaSS assay mixture with mixture of (*E*)-farnesene for the characterization of product 6, **J)** *exo*-β-Bergamotene (**5**), **K)** Co-injection of SaSS assay mixture with *exo*-β-Bergamotene, **L)** (*E*)-β-Farnesene (**6**), **M)** Co-injection of SaSS assay mixture with (*E*)-β-Farnesene, α-Santalene (**1**), β-Santalene (**2**), *epi*-β-Santalene (**3**), *exo*-α-Bergamotene (**4**), *exo*-β-Bergamotene (**5**), (*E*)-β-Farnesene (**6**), synthesized mixture of (6*E*)-Farnesenes (**I**, **II**, **III**).

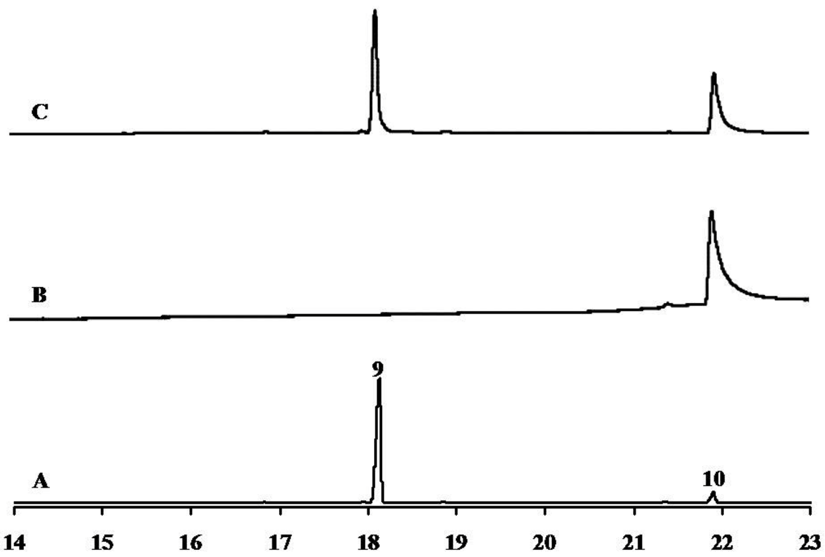

Figure S66: Co-injection of α-bisabolol with SaBS assay mixture, **A)** GC profile of SaBS assay with (*E*,*E*)-FPP, **B)** α-bisabolol (**10**) standard, **C)** Co-injection of SaBS assay mixture with α-bisabolol.

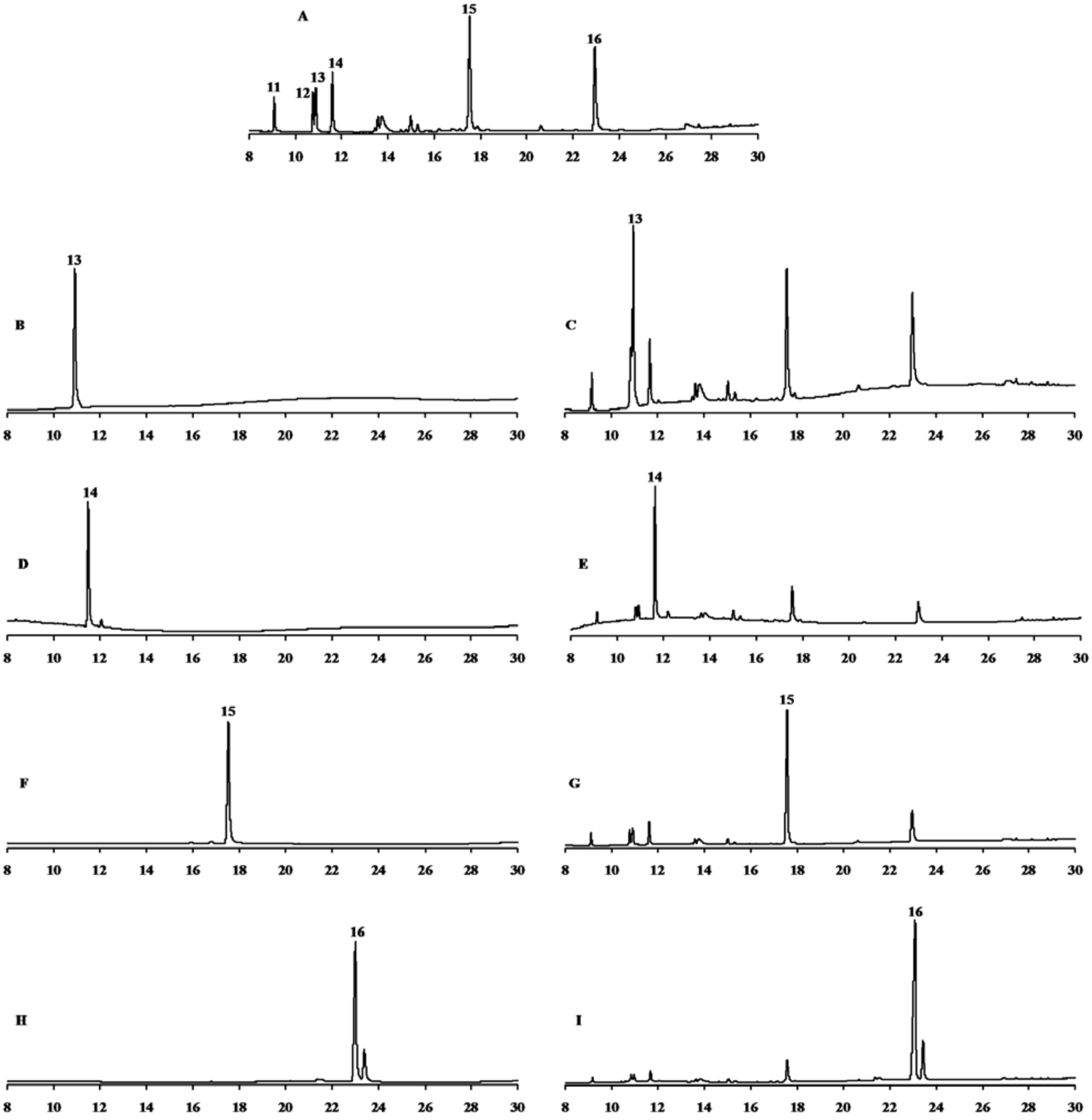

Figure S67: Co-injection standard terpenes with SaSQS1 assay mixture with GPP for the characterization of metabolites, **A)** Product profile of SaSQS1 assay with GPP, **B)** β-Pinene (**13**) standard, **C)** Co-injection of SaSQS1 assay mixture with β-pinene, **D)** Myrcene (**14**) standard, **E)** Co-injection of SaSQS1 assay mixture with myrcene, **F)** Linalool mixture of isomer (**15**), **G)** Co-injection of SaSQS1 assay mixture with linalool mixture of isomer, **H)** α-Terpineol (**16**), **I)** Co-injection of SaSQS1 assay mixture with α-terpineol.

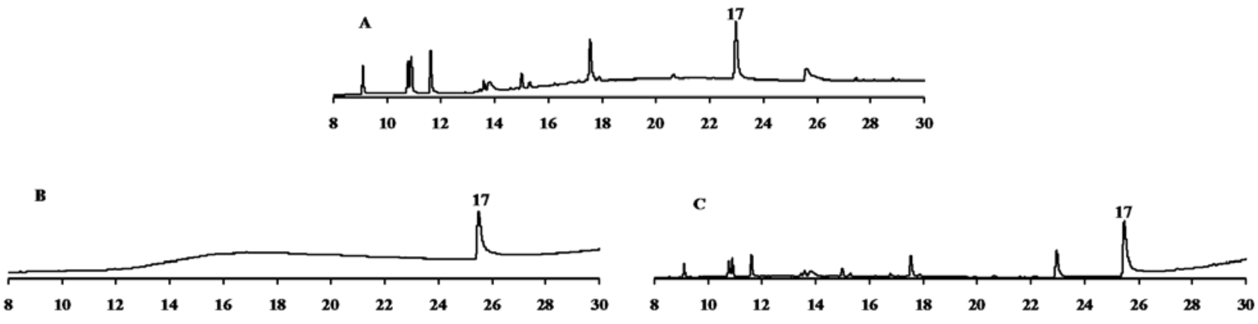

Figure S68: Co-injection standard terpenes with SaSQS1 assay mixture with NPP for the characterization of metabolites, **A)** Product profile of SaSQS1 assay with NPP, **B)** nerol (**17**) standard, **C)** Co-injection of SaSQS1 assay mixture with nerol.

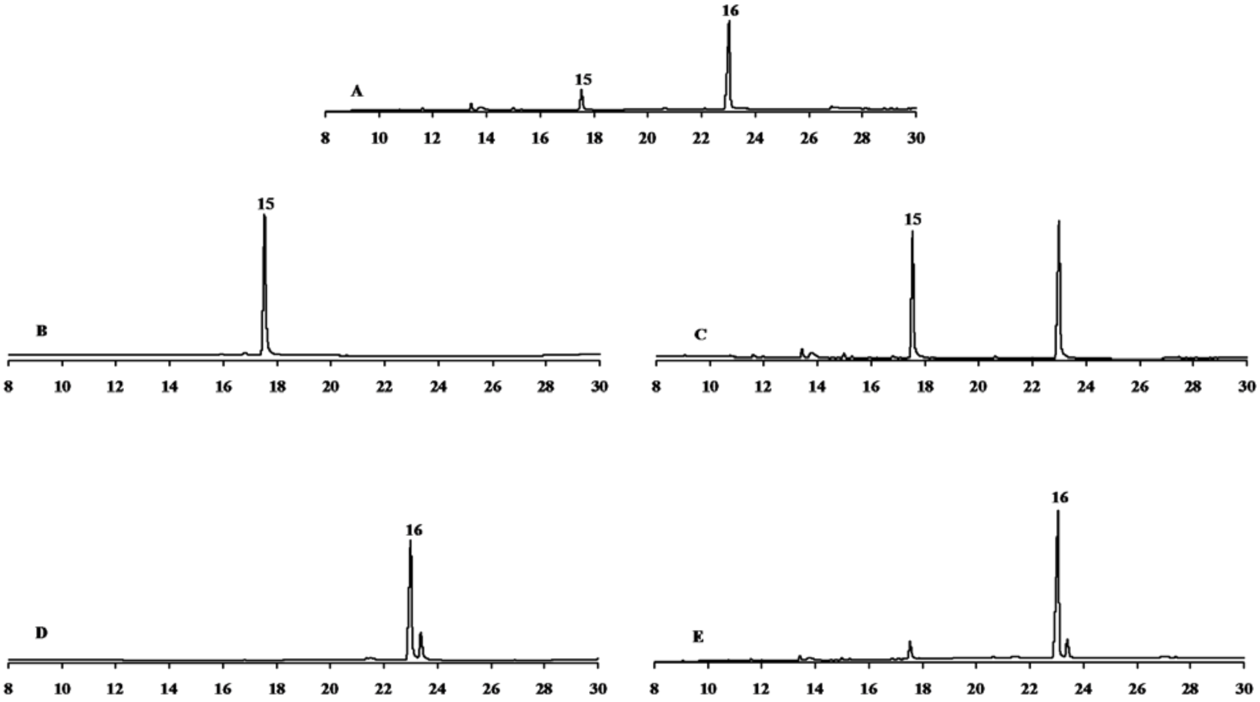

Figure S69: Co-injection of standard terpenes with products from incubation of SaBS with GPP, **A)** Product profile of SaBS assay with GPP, **B)** linalool (**15**) standard, **C)** Co-injection of SaBS assay mixture with linalool, **D)** α-terpineol (**16**) standard, **E)** Co-injection of SaBS assay mixture with α-terpineol.

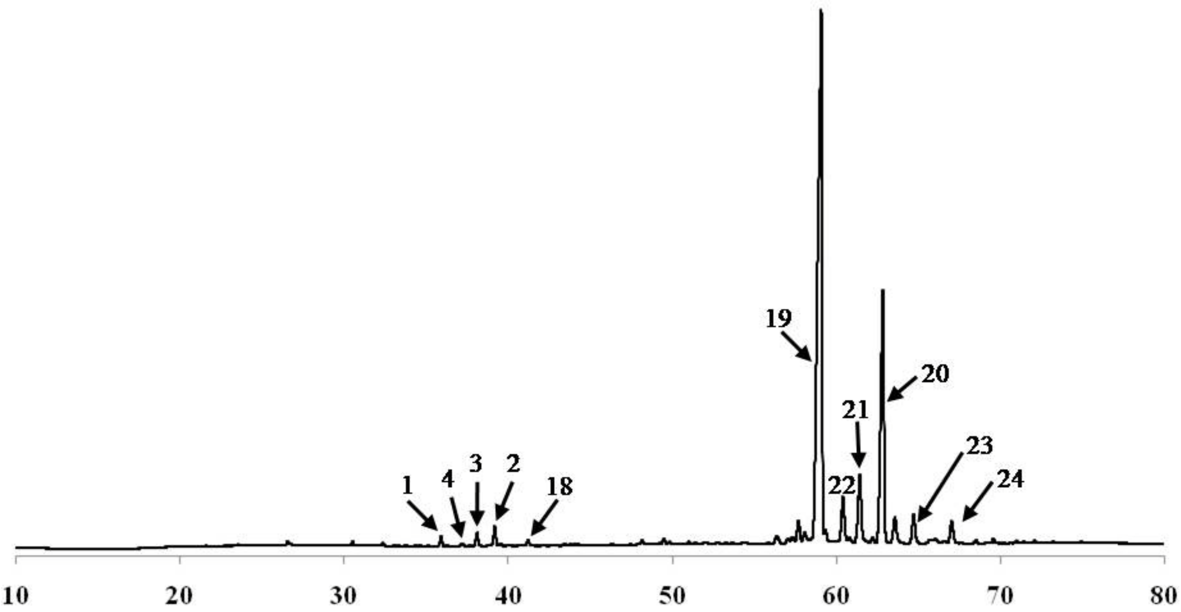

Figure S70: GC-FID chromatogram of heartwood oil isolated from *S. album* using a HP-5 capillary column (30 m × 0.32 mm × 0.25 µm, J & W Scientific). Peak identification: **1**: α-santalene, **2**: β-santalene, **3**: *epi*-β-santalene, **4**: *exo*-α-bergamotene and **18**: α-curcumene, **19**: (*Z*)-α-santalol, **20**: (*Z*)-β-santalol, **21**: (*Z*)-*epi*-β-santalol, **22**: (*Z*)-*exo*-α-bergamotol, **23**: (*E*)-nuciferol and **24**: (*Z*)-lanceol.
